# Relationship between loss of desiccation tolerance and programmed cell death (PCD) in mung bean (*Vigna radiata*) seeds

**DOI:** 10.1371/journal.pone.0218513

**Published:** 2019-07-02

**Authors:** Xiangrong Tian, Sidi Li, Qing Zeng, Wei Huang, Xuanming Liu, Songquan Song

**Affiliations:** 1 Hunan Provincial University Key Laboratory of Plant Resources Protection and Utilization of Wuling Mountain Area, Jishou University, Jishou, Hunan, China; 2 Hunan Provincial Key Laboratory of Medicinal Plants and Plant Medicine, Huaihua University, Huaihua, Hunan, China; 3 College of Biology, Hunan University, Changsha, Hunan, China; 4 Southern China Botanical Garden, Chinese Academy of Sciences, Guangzhou, Guangdong, China; 5 Institute of Plant Research, Chinese Academy of Sciences, Beijing, China; National Institute of Technology, Rourkela, INDIA

## Abstract

Mung bean (*Vigna radiata*), an important legume crop, has the property of desiccation tolerance (DT), which is lost in the final stage of germination (preimbibition, 18 h-24 h). We compared parameters related to the programmed cell death (PCD) of mung bean seeds before and after dehydration at different imbibition stages through various detection methods. The results of Evans blue and TTC staining methods showed that the dehydration process could lead to cell death. The results of optical and subcellular morphology showed that PCD occurred after dehydration. The destruction of DNA integrity and the activity changes in caspase and total nuclease in mung bean seeds after dehydration treatment indicated that the loss of desiccation tolerance was related to PCD. Dehydration resulted in the destruction of the mitochondrial structure, reversal of the membrane potential, and the entrance of cytochrome C into the cytoplasm. These processes all indicate that the mitochondrial apoptosis pathway was the main form of dehydration-induced PCD. The results of cytoplasmic Ca^2+^ concentration showed that Ca^2+^ signaling also played a role in inducing PCD, with the upstream signal being dehydration-induced changes in water potential and the downstream signal being the ROS and mitochondrial PT channel, according to the order in which these signals happened. The mitochondrial apoptosis pathway can be considered the main mechanism of dehydration-induced PCD based on our analysis of the sequence of major events in PCD. The main processes include dehydration induction, changes in Ca^2+^ and mitochondrial respiratory electron transport, the reversal of mitochondrial membrane potential induced by ROS and Ca^2+^, and the transmission and execution of PCD downstream signals induced by cytochrome C release.

## Introduction

Desiccation tolerance (DT) refers to the ability of plant tissues to survive at very low water levels (below 5%) [1]. DT is widespread in microorganisms, animals and plants for coping with stress environments and protecting their own tissues from dehydration damage. Mung bean (*Vigna radiata*) is a fast-growing leguminous plant that also has desiccation tolerance. However, we found that the desiccation tolerance of mung bean seeds was lost at the telophase of germination (preimbibition, 18 h-24 h).

Programmed cell death (PCD) in plants is a spontaneous and programmed cell death process regulated by certain gene and protein pathways that has been a hot topic in plant physiology and molecular biology research in recent years. At present, it can be divided into three types, as follows: a) apoptosis; b) autophagy or cytoplasmic degenerative PCD; and c) lysosomal degenerative PCD. Among these, apoptosis is the most common form. In apoptosis, DNA is marginalized on the nuclear membrane (condensation) and is divided into lengths of the nucleosome size, the nucleus and cytoplasm are decomposed into vesicles, and macrophages take away bodies in vivo [2]. Apoptotic signals are divided into endogenous and exogenous signals according to their source [3]. The endogenous pathway begins in mitochondria, which bind cytochrome C released by mitochondria to the activator of apoptosis, protease 1, thereby initiating apoptosis. Based on the study of cell death during seed development by Golovina *et al*. [4], it is suggested that the loss of seed desiccation tolerance may be related to PCD, but there is no direct evidence of PCD or the pathway of its occurrence. We speculate that desiccation-sensitive seeds (or hypocotyls) may damage the structure and function of mitochondria under water stress. Apoptogenic factors such as cytochrome C are released from mitochondria into cytosols, and cysteine proteases are activated, leading to PCD. In this study, mung bean seeds (hypocotyls) with different levels of desiccation tolerance/sensitivity during preimbibition were used as materials to establish an experimental model of desiccation tolerance loss/desiccation sensitivity. By analyzing the morphological changes and physiological and biochemical indexes of PCD events, it was confirmed that the loss of desiccation tolerance might be caused by the death of hypocotyl cells by PCD via the mitochondrial pathway.

## Materials and methods

### Plant materials and treatment

*Vigna radiata* (Linn.) Wilczek. Cv. Zhonglv No. 1 as the test material was purchased at the Chinese Academy of Agricultural Sciences. The seeds ripened in the same year, and the time of purchase was approximately 60 days after harvest. The purchased seeds, with full grains and of similar sizes, were stored in an environment at 15°C and 50% RH for further use. The mung bean seeds wrapped in filter paper were placed in a culture dish (Φ = 12 cm) after being sterilized with 1% (W/V) NaC1O and adding 25 ml deionized water. In a dark environment at 20°C, the preimbibition process occurred for 0, 3, 6, 18 and 24 h, and dehydration was conducted in dry air (silica gel) for 25 h. The hypocotyls of the mung beans were removed before and after dehydration.

### Viability stain

The mung bean hypocotyls in different preimbibition and dehydration states were used to make transverse sections near the root end and median longitudinal sections between the two cotyledons. Evans blue staining and TTC staining were used to stain the hypocotyls, and the staining results were recorded under a stereomicroscope.

### Observation of optical microscopy

Mung bean hypocotyls in different preimbibition and dehydration states were cut, and 3–5 mm sections were taken at the root end. The hypocotyls were then fixed with FAA solution for 72 h, dehydrated in gradient alcohol, embedded in paraffin, sliced, dewaxed with xylene solution, stained with 0.5% (W/V) toluidine blue O, and recorded by optical microscopy.

### Observations of the ultrastructure of histiocytic cells

Mung bean hypocotyls in different preimbibition and dehydration conditions were cut, and 2–4 mm parts were taken at the root end and then fixed with 5% (V/V) glutaraldehyde solution at 4°C for 120 h. After being rinsed with sodium phosphate buffer solution (0.1 mol ▪ L^-1^, pH 7.2), the sections were fixed with 1% (W/V) osmium acid for 4 h, dehydrated with gradient alcohol, embedded in Spurr resin, and sliced, and the subcellular structure was observed by electron microscopy.

### DNA Integrity checking

#### Electrophoresis of total DNA

The total DNA was extracted according to Kang et al., Young and Qiao Aimin’s methods [5, 6]. Gel electrophoresis was used based on Young’s and other methods [7, 5], and the gel imaging system recorded the results.

#### TdT-mediated dUTP-biotin nick end labeling (TUNEL)

The terminal deoxynucleotidyl transferase (TdT)-mediated dUTP-biotin nick end labeling (TUNEL) method has been employed widely to demonstrate the occurrence of PCD in cells in routinely prepared paraffin sections. Cells undergoing PCD have large numbers of DNA fragments with free 3’-OH terminals. Biotin-labeled dUTP can be incorporated into the 3'-OH terminal of DNA fragments in a reaction catalyzed by the TdT enzyme and specifically binds to streptavidin-linked peroxidase. Then, this antigen-antibody complex can be detected by peroxidase reaction with H_2_O_2_ and 3,3’-diaminobenzidine (DAB) as a chromogen. In our experiment, mung bean hypocotyls in different preimbibition and dehydration states were taken, and paraffin sections were made according to the method of observation on the ultrastructure of histiocytic cells. The results were recorded according to the TdT-FragEL DNA fragmentation detection kit (No. QIA33) specifications of Merck Company (formerly Oncogene) [8].

#### Nucleosome ELISA

Mung bean hypocotyls with different preimbibition and dehydration conditions were obtained. The CalBiochem kit Nucleosome ELISA (No. QIA25) [9], as provided by Boubriak, was used to calculate the number of nucleosomes per unit U/mg protein.

#### Detection of total nuclease activity

The activity of total nuclease was determined in accordance with the method of He and Kermode [10]. Mung bean hypocotyls were ground into a uniform powder in liquid nitrogen and homogenized on ice in the extract buffer solution (0.15 mol ▪ L^-1^ Tris-HCl buffer solution, pH 6.8; 1 mmol ▪ L^-1^ DTT; 0.5 mmol ▪ L^-1^ benzyl sulfonyl fluoride; 20 μmol ▪ L^-1^ leucine inhibitory peptide). After centrifugation at 15 000 g for 10 min, the supernatant was removed for determination of the total nuclease activit*y*.

### Detection of caspase activity

Mung bean hypocotyls at different preimbibition and dehydration states were ground into homogeneous powders in liquid nitrogen. The activities of caspase-3 and caspase-9 were determined based on the specifications of the caspase-3 activity assay kit (C1116) and the caspase-9 activity assay kit (C1158).

### Detection of key events in the mitochondrial apoptotic pathway

#### Mitochondrial membrane potential detection

The protoplast was prepared according to Feng Haitao’s method [11] and stained according to the instructions of Beyotime Biotechnology Company’s mitochondrial membrane potential (Δ ψm) JC-1 Detection Kit (No. C2006). The results were observed by fluorescence microscopy and analyzed by flow cytometry.

#### In situ histochemical localization of cytochrome C

The paraffin sections made for morphological observations were used for fluorescence immunohistochemical detection with reference to the methods [12] in the guidelines for molecular biology experiments.

#### Determination of cytoplasmic Ca^2+^ concentration

The cytoplasmic Ca^2+^ concentration of mung bean hypocotyl protoplasts was determined by the method of Zhang Juntian et al. [13] and Wang Zejun et al. [14].

### Statistical analysis

Data were expressed as the means ± SDs. Statistical analysis of the data for the different preimbibition phases with/without dehydration treatment was performed through paired *t*-test, in which *P* < 0.05 was considered significant.

## Results

### Dehydration causes cell death of meristem cells

**Fig 1:**
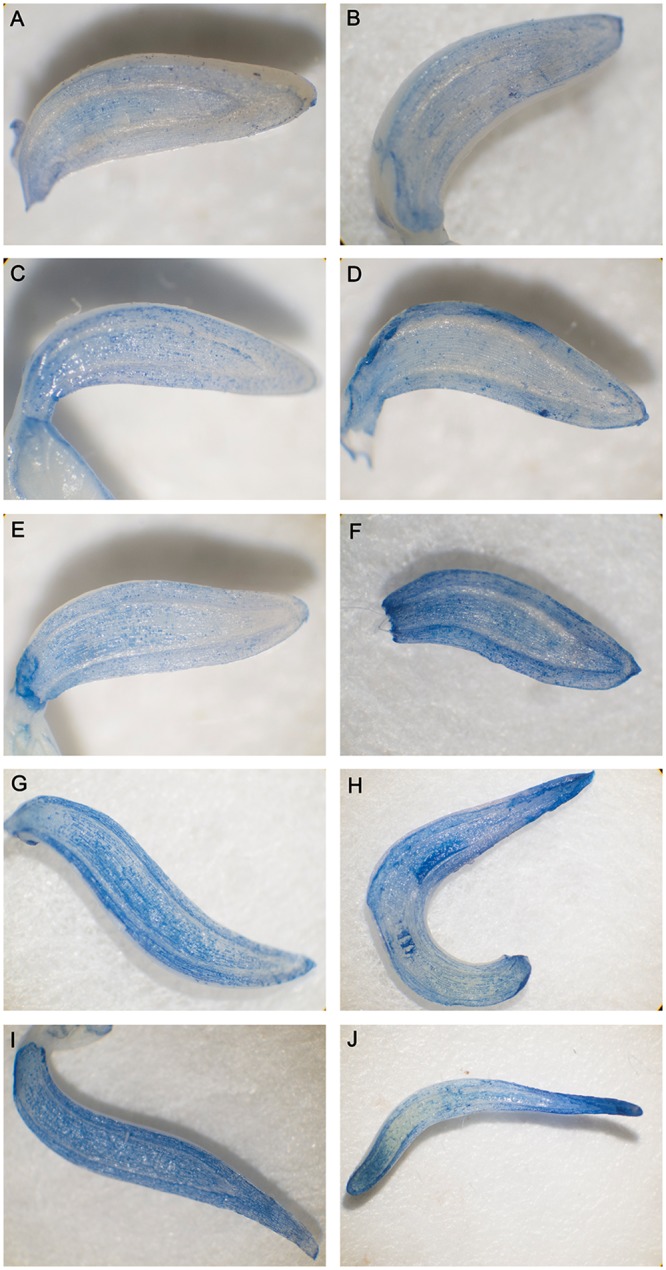
Evans blue staining of mung bean hypocotyls in different preimbibition and dehydration states. A, C, E, G and I represent the longitudinal section of the hypocotyl at 0, 3, 6, 18 and 24 h of preimbibition, respectively. B, D, F, H and J represent the longitudinal section of the hypocotyl at 0, 3, 6, 18 and 24 h of preimbibition after 24 h of dehydration, respectively.

**Fig 2:**
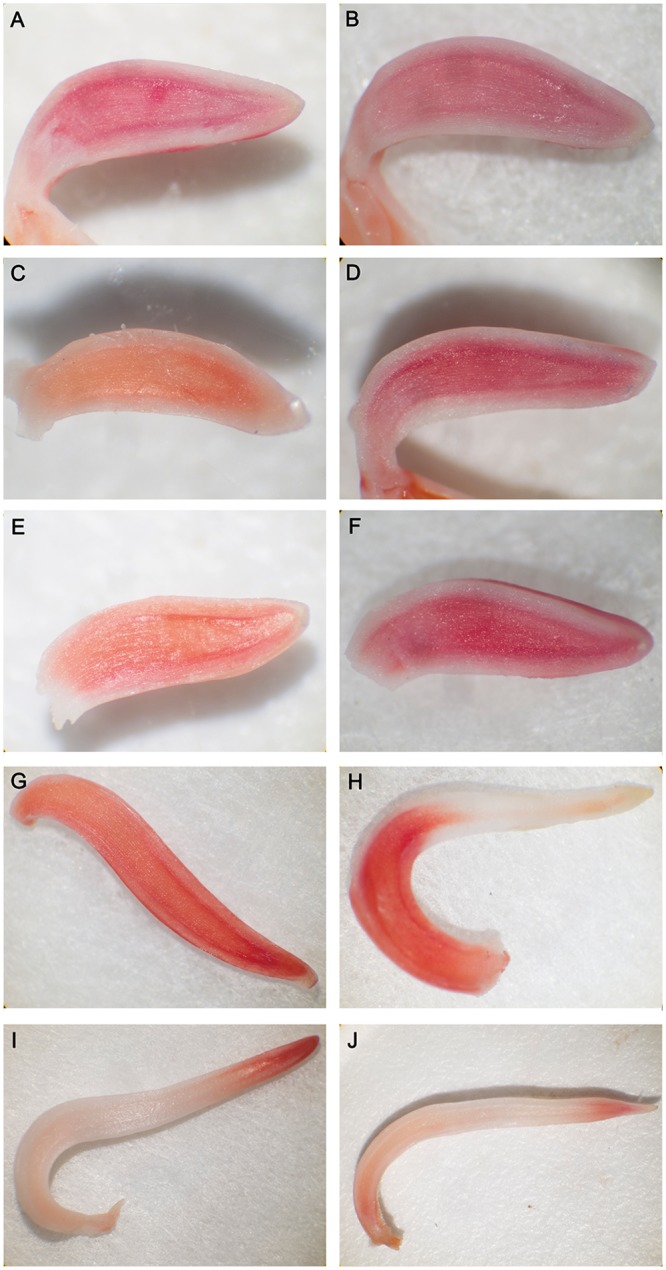
TTC staining of mung bean hypocotyls in different preimbibition and dehydration states. A, C, E, G and I represent the longitudinal section of the hypocotyl with 0, 3, 6, 18 and 24 h of preimbibition, respectively. B, D, F, H and J represent the longitudinal section of the hypocotyl with 0, 3, 6, 18 and 24 h of preimbibition after 24 h of dehydration, respectively.

Evans blue staining is a method for the rapid identification of cell death in which the dead cells are dyed blue. A clear increase in cell death in the mung bean hypocotyl due to dehydration was observed in a timely manner. The upper cortex of dehydrated mung bean hypocotyl was markedly colored after 3 h of preimbibition, the cortex and pith of hypocotyl were obviously stained after 6 h of preimbibition, and the meristem of hypocotyl showed obvious coloration after preimbibition for 18 h and 24 h (Fig 1 and S1 Supporting Information). The results of TTC staining (surviving cells are dyed red) indicated that with the increase in imbibition time, the base color of meristem gradually deepened, and the darkest color in the meristem was found at 18–24 h imbibition (Fig 2 and S2 Supporting Information).

The above staining results (Figs 1 and 2) clearly suggest that the late stage of preimbibition is the stage of loss of desiccation tolerance, which leads to cell death in meristem cells and may be the main cause of the decline in vitality due to dehydration in the later stage of preimbibition.

### Dehydration induces morphological changes in hypocotyl tissue cells

**Fig 3:**
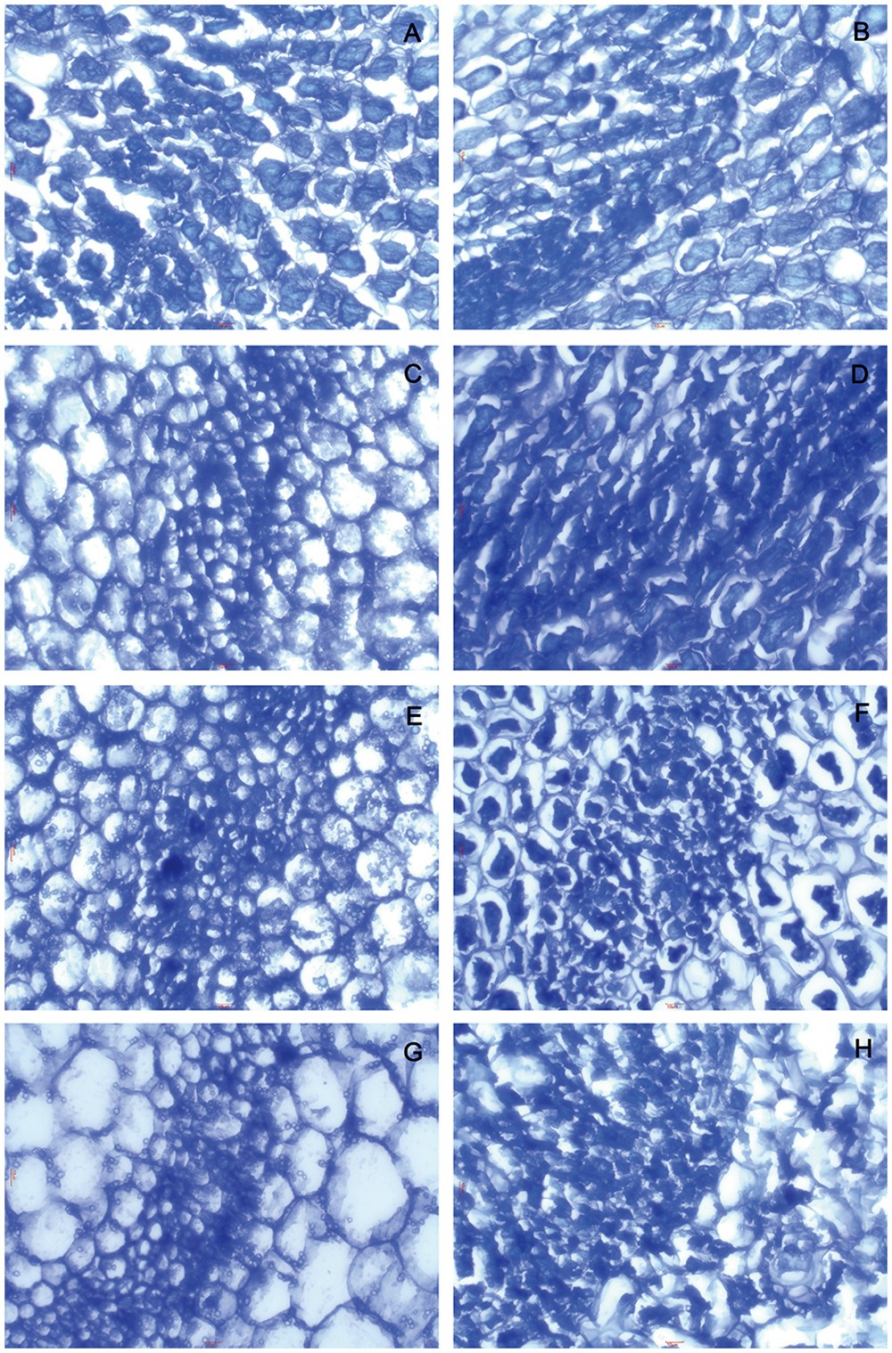
Toluidine blue staining of mung bean hypocotyls in different preimbibition and dehydration states. A, C, E and G show the transverse sections of the hypocotyl with 3, 6, 18 and 24 h of preimbibition, respectively. B, D, F and H show the transverse sections of the hypocotyl with 3, 6, 18 and 24 h of preimbibition after 24 h of dehydration, respectively.

The tissue cell morphology of mung bean hypocotyl stained with toluidine blue O at different preimbibition periods with/without dehydration treatment is presented in Fig 3 and S3 Supporting Information. For various periods (except at 3 h) without dehydration treatment, the cell morphology was full and intact, the nucleus and cytoplasm were clear, and the cell staining in all components except the nucleus was light. After 24 h of dehydration, morphological changes in cells, such as cell shrinkage and nucleus elongation, appeared in accordance with the increasing time of preimbibition, indicating that the morphological changes to cells in the late stage of imbibition are consistent with the morphological characteristics of cell death.

### Dehydration induces subcellular structural changes in the mung bean hypocotyl

**Fig 4:**
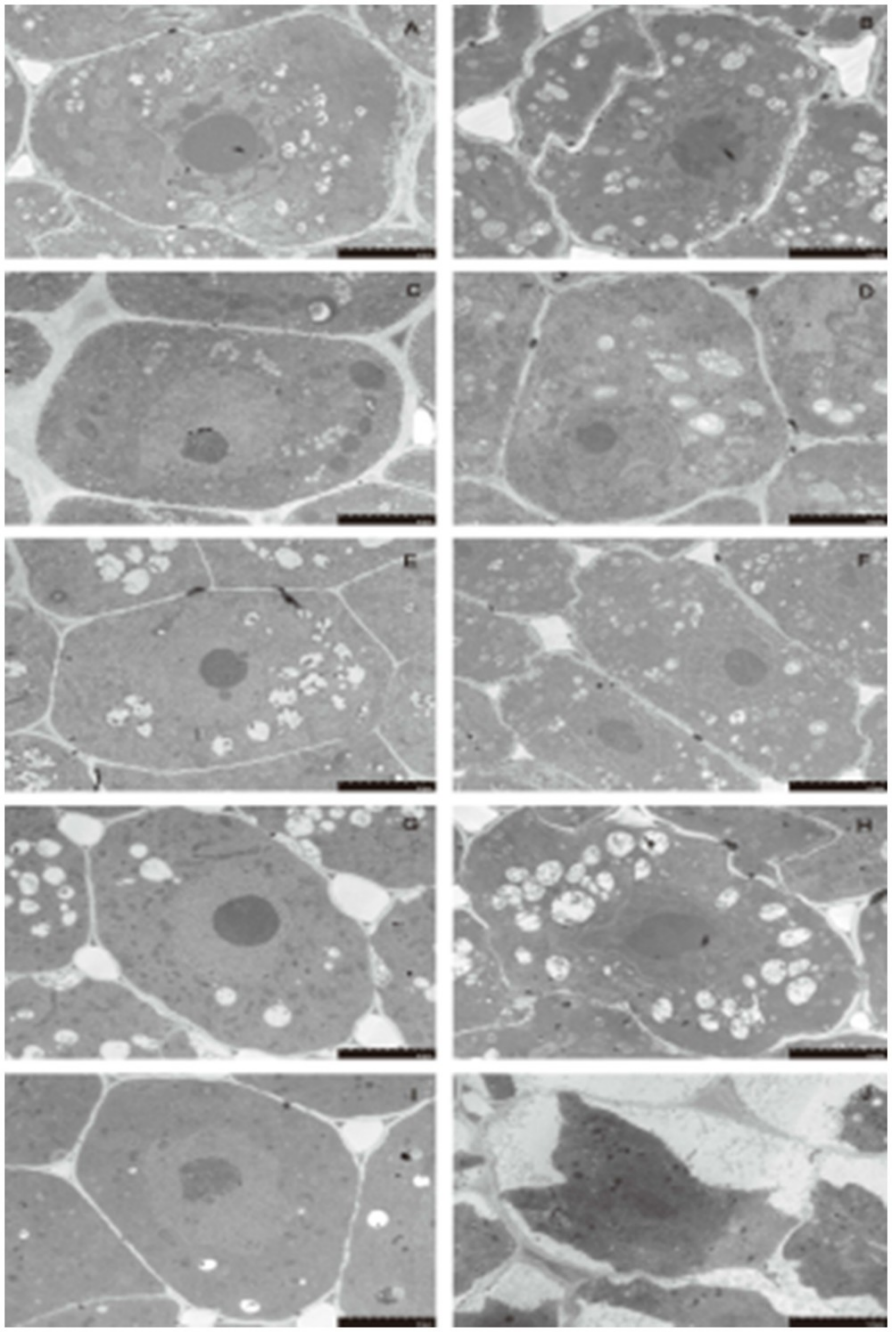
Subcellular structure of meristematic cells from mung bean hypocotyls in different preimbibition and dehydration states. A, C, E, G and I show meristematic cells of the hypocotyl with 0, 3, 6, 18 and 24 h of preimbibition, respectively. B, D, F, H and J show meristematic cells of the hypocotyl with 0, 3, 6, 18 and 24 h of preimbibition after 24 h of dehydration, respectively.

**Fig 5:**
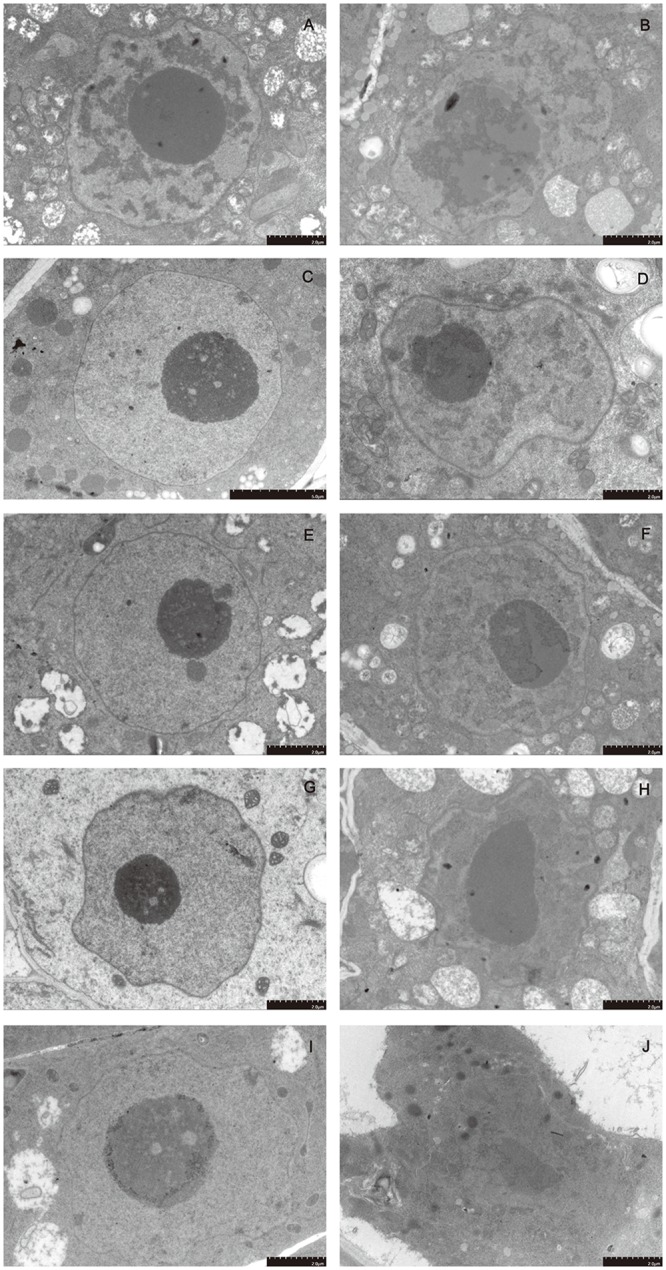
Ultrastructure of meristematic cell nuclei in different preimbibition and dehydration states. A, C, E, G and I show meristematic cell nuclei of the hypocotyl with 0, 3, 6, 18 and 24 h of preimbibition, respectively. B, D, F, H and J show meristematic cell nuclei of the hypocotyl with 0, 3, 6, 18 and 24 h of preimbibition after 24 h of dehydration, respectively.

**Fig 6:**
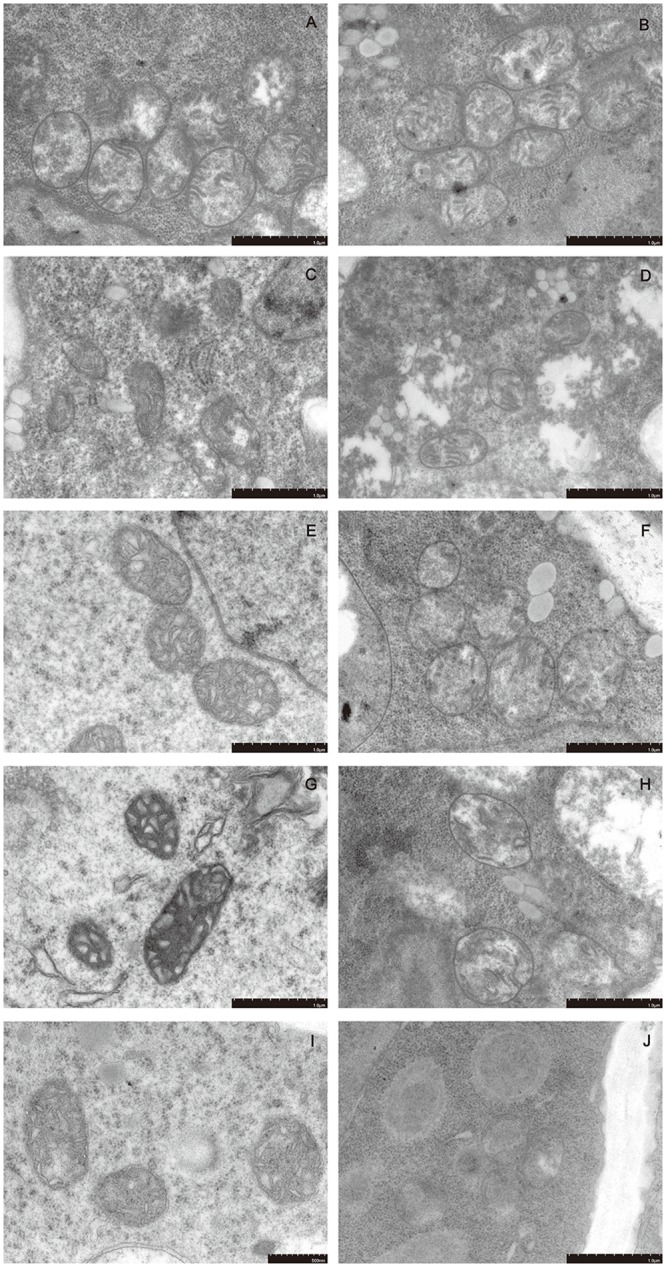
Ultrastructure of meristematic cell mitochondria in different preimbibition and dehydration states. A, C, E, G and I show meristematic cell mitochondria of the hypocotyl at 0, 3, 6, 18 and 24 h of preimbibition, respectively. B, D, F, H and J show meristematic cell mitochondria of the hypocotyl with 0, 3, 6, 18 and 24 h of preimbibition after 24 h of dehydration, respectively.

The ultrastructures of the cell morphology of mung bean hypocotyls at different preimbibition periods with/without dehydration treatment are shown in Figs 4–6 and S4–S6 Supporting Information. During normal imbibition, the cell morphology of the hypocotyl tissue was plump, the cytoplasm and nucleus were distinct, the cell size and the ratio of nucleus to cytoplasm were stable, and the number of vacuoles increased with time. However, after 24 h of dehydration, the cell morphology, nucleus morphology and mitochondrial morphology of the meristem changed gradually with the time of imbibition. The cytoplasmic membrane boundary of hypocotyl meristem cells with 24 h of preimbibition is absent while the nuclear membrane disappeared, the nucleus became indistinct, and the outer mold of the mitochondria was destroyed, which conformed to the basic characteristics of PCD. The mitochondrial morphological changes occurred earlier than the nuclear morphological changes. Therefore, the results suggest that apoptosis in the mitochondria may induce PCD.

### Dehydration causes DNA damage in mung bean hypocotyls

#### Degradation of total DNA

The DNA bands of mung bean hypocotyls with normal imbibition were not tailed, which indicates that the DNA of the hypocotyl was intact and did not degrade significantly. However, after dehydration, the tail of DNA bands increased significantly with prolonged preimbibition duration, indicating significant DNA degradation (S1 Fig and S7 Supporting Information).

#### Appearance of TUNEL labeling cells

TUNEL-labeled cells can be stained yellow, yellow-green and brown-yellow. During normal imbibition, there were no TUNEL-labeled cells in the hypocotyl tissue, showing a light gray background. After dehydration, the onset time of TUNEL labeling in preimbibed mung bean hypocotyls was 18 h, accompanied by an increase in the number of TUNEL-labeled cells with the prolongation of preimbibition time, which proved that PCD occurred after 18 h of preimbibition (S2 Fig and S8 Supporting Information).

#### Increase in the number of nucleosomes

**Fig 7:**
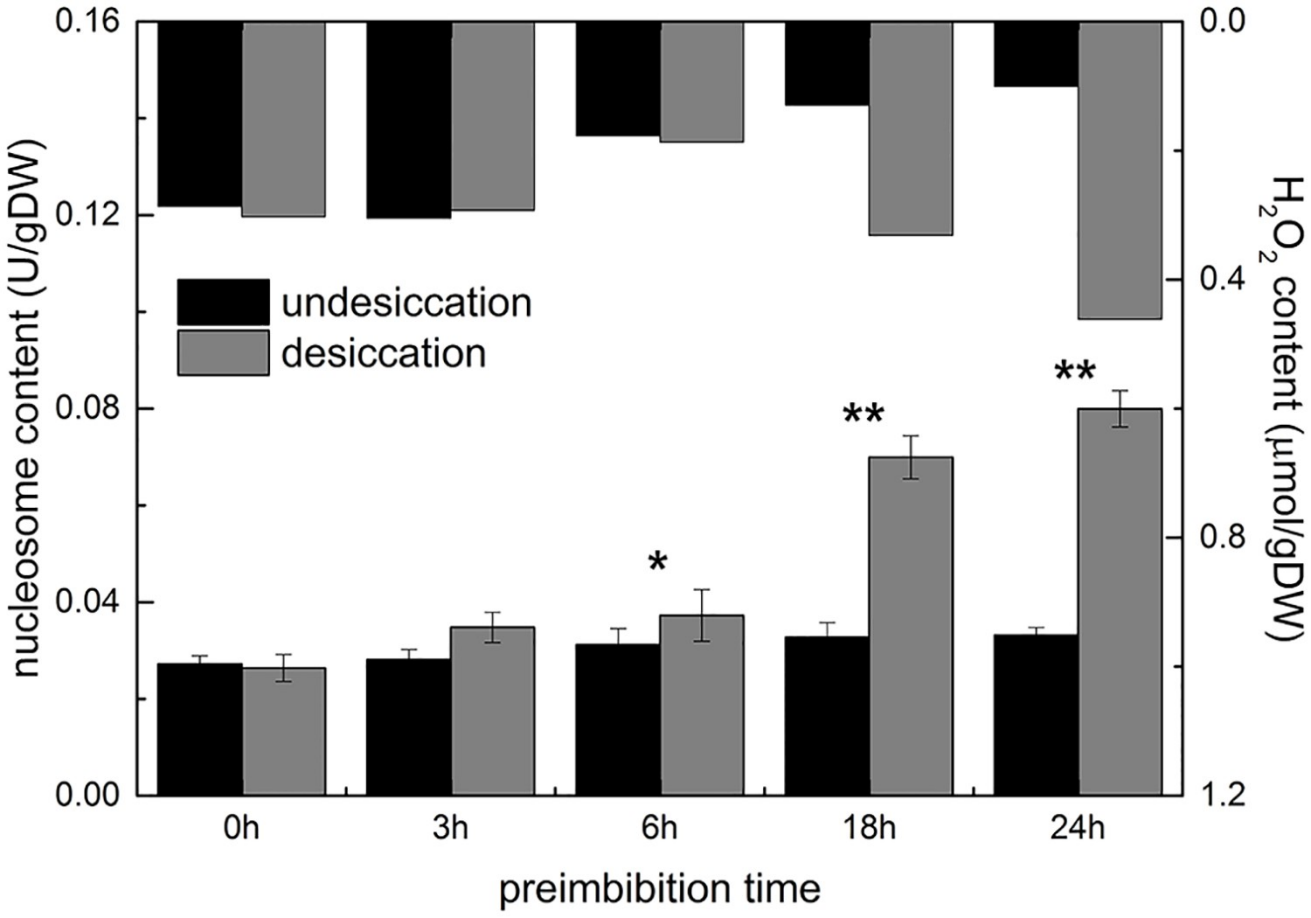
ELISA quantity of nucleosomes from mung bean hypocotyls in different preimbibition and dehydration states. * *P* < 0.05; ** *P* < 0.01.

In normal imbibition, the number of nucleosomes in unit cell numbers is 0.033–0.037 U/g DW with no significant change. When the preimbibition duration reached 18–24 h, with the prolongation of imbibition time after dehydration, the number of nucleosomes increased gradually. The number of nucleosomes imbibed for 18–24 h reached 0.077–0.080 U/g DW, which was approximately 2 times the amount in nonimbibition conditions (Fig 7 and S9 Supporting Information).

#### Increase in total nuclease activity

**Fig 8:**
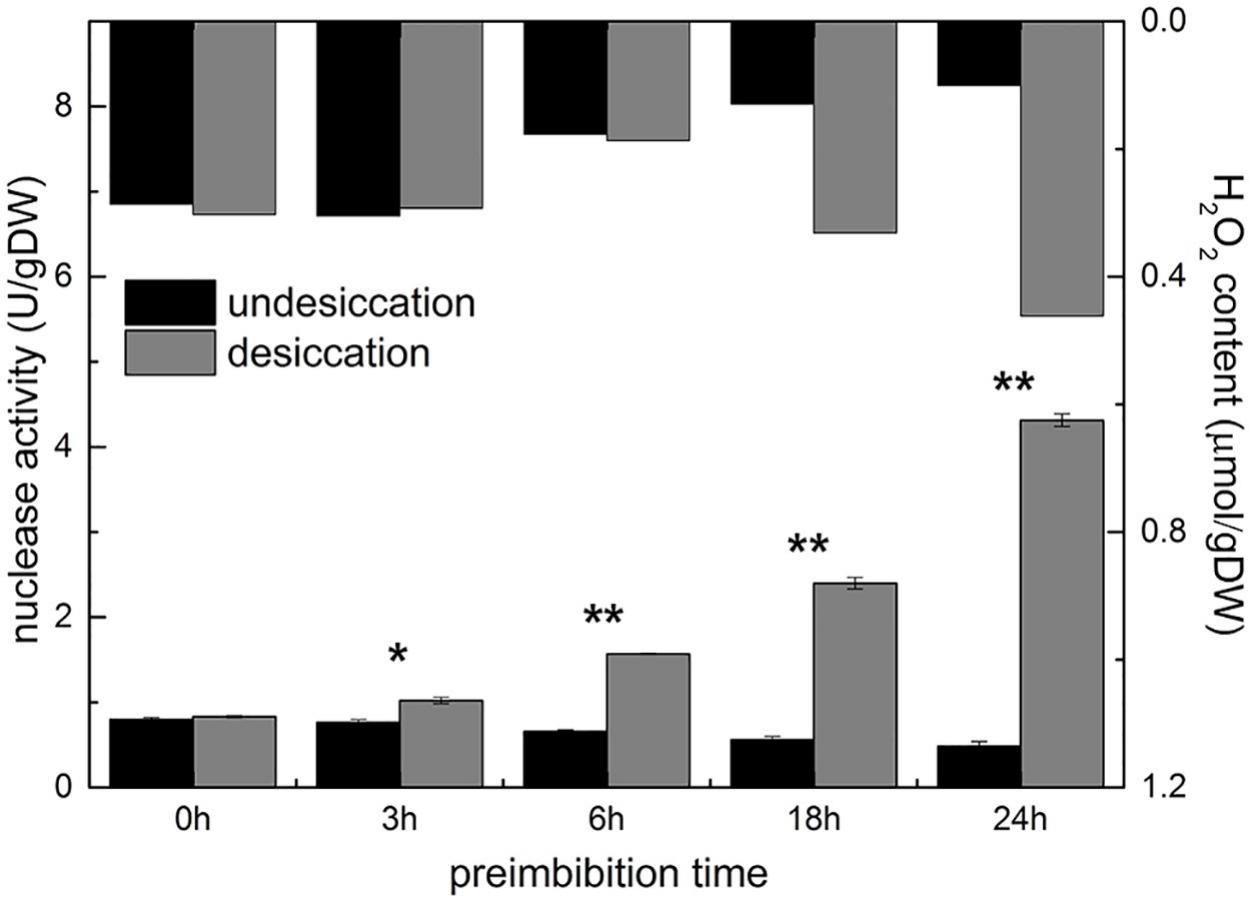
Activity of total nuclease from mung bean hypocotyls in different preimbibition and dehydration states. * *P* < 0.05; ** *P* < 0.01.

Compared with normal imbibition, dehydration at the middle and late stages of preimbibition (6–24 h) significantly increased the total nuclease activity, which showed that the total nuclease activity at 24 h (4.313±0.074 U/g DW) was approximately 2.7 times higher than that at 6 h (1.568±0.007 U/g DW). In terms of comparisons of total nuclease activity and nucleosome content changes, the obvious magnitude of the change indicated that the site of total nuclease cleavage was not confined to the junction of nucleosomes, which also confirmed the results of DNA electrophoresis (Fig 8 and S9 Supporting Information).

### Dehydration increases caspase-like activity

**Fig 9:**
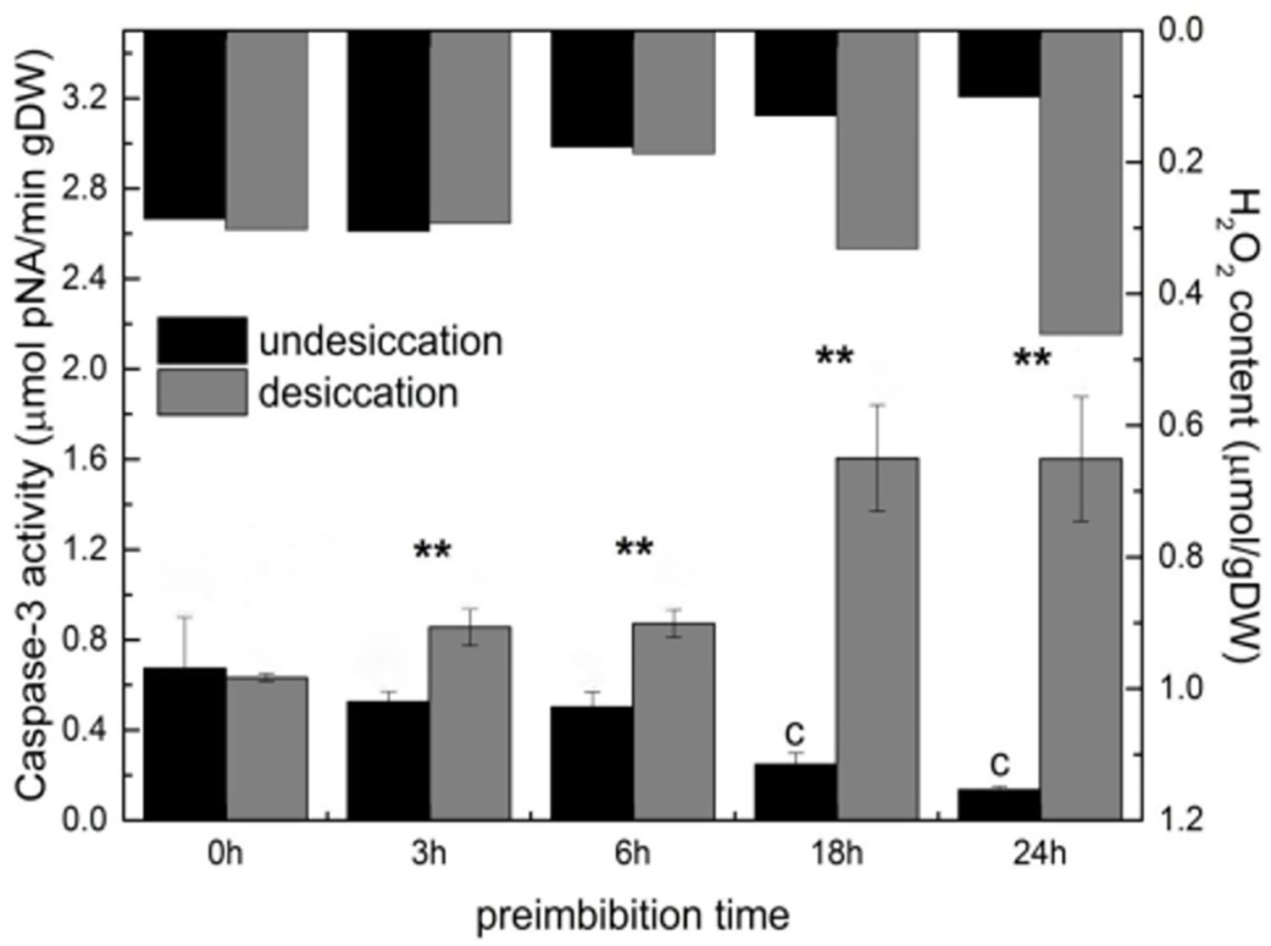
Activity of caspase-3 in mung bean hypocotyls in different preimbibition and dehydration states. ** *P* < 0.01.

**Fig 10:**
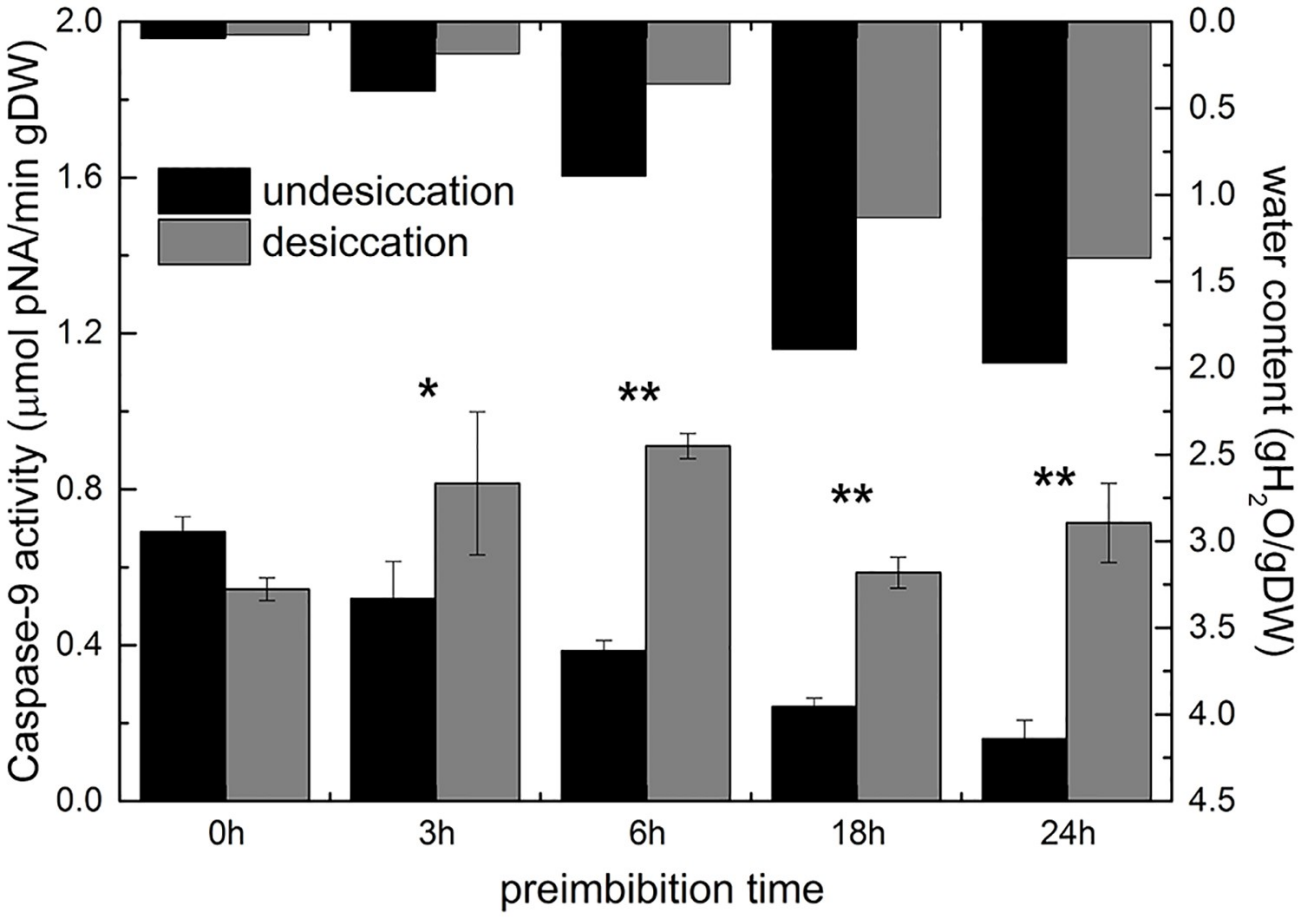
Activity of caspase-9 in mung bean hypocotyls in different preimbibition and dehydration states. * *P* < 0.05; ** *P* < 0.01.

After dehydration, the activity of caspase-3 and caspase-9 increased significantly with the prolongation of imbibition time, especially during the middle and late preimbibition period (6–24 h) (Figs 9 and 10 and S10 Supporting Information).

### Dehydration reverses mitochondrial membrane potential

**Fig 11:**
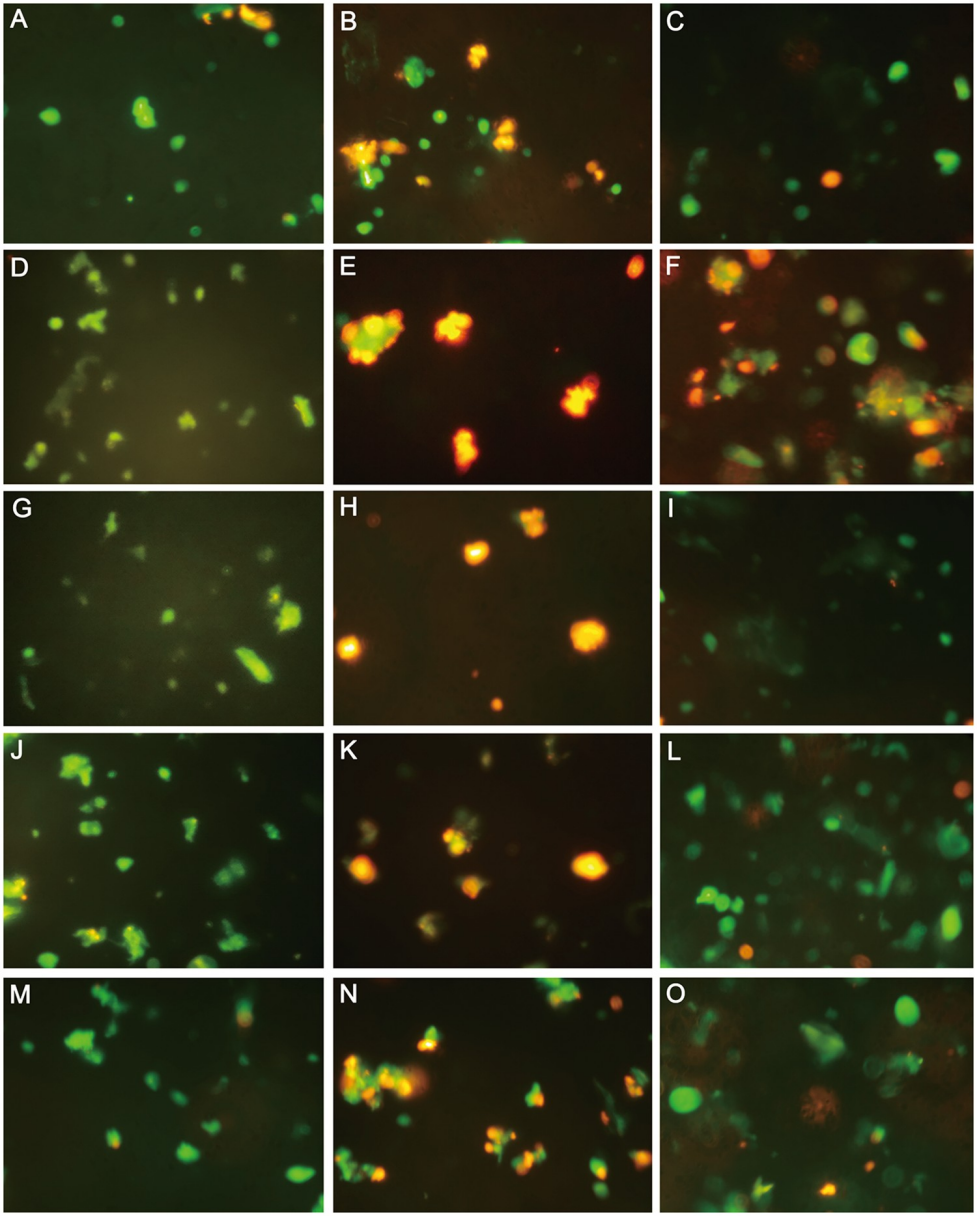
JC-1 staining of protoplasts from mung bean hypocotyls in different preimbibition and dehydration states. A, D, G, J and M show the positive control of preimbibed protoplasts 3, 6, 18 and 24 h after the CCCP treatment, respectively. B, E, H, K and N show the staining of preimbibed protoplasts at 0, 3, 6, 18 and 24 h, respectively. C, F, I, L and O show the preimbibed protoplasts with 0, 3, 6, 18 and 24 h after 24 h of dehydration, respectively.

The protoplast staining of normal imbibed hypocotyls was red, indicating low mitochondrial potential. After dehydration, the protoplast staining of preimbibition (6–24 h), especially at 18–24 h, was similar to that of CCCP positive, indicating that mitochondrial membrane potential reversed (Fig 11 and S11 Supporting Information). To prove the quantitative accuracy of the results, flow cytometry was used to analyze the protoplasts stained with JC-1 in different preimbibition and dehydration states. The results showed, consistent with the protoplast staining, no obvious change in mitochondrial membrane potential in the undehydrated protoplasts (S3 Fig and S12 Supporting Information).

1. Regardless of the degree of imbibition and water content, the protoplast particles migrated from the upper quadrant to the lower quadrant in flow cytometry analysis; that is, dehydration could lead to a change in mitochondrial membrane potential.
2. With the increase in hydration, the proportions and degree of downward migration of protoplast particles increased significantly. After 18 h of preimbibition, there were almost no protoplast particles in situ in the upper quadrant.

### Dehydration leads to the release of cytochrome C into the cytoplasm

**Fig 12:**
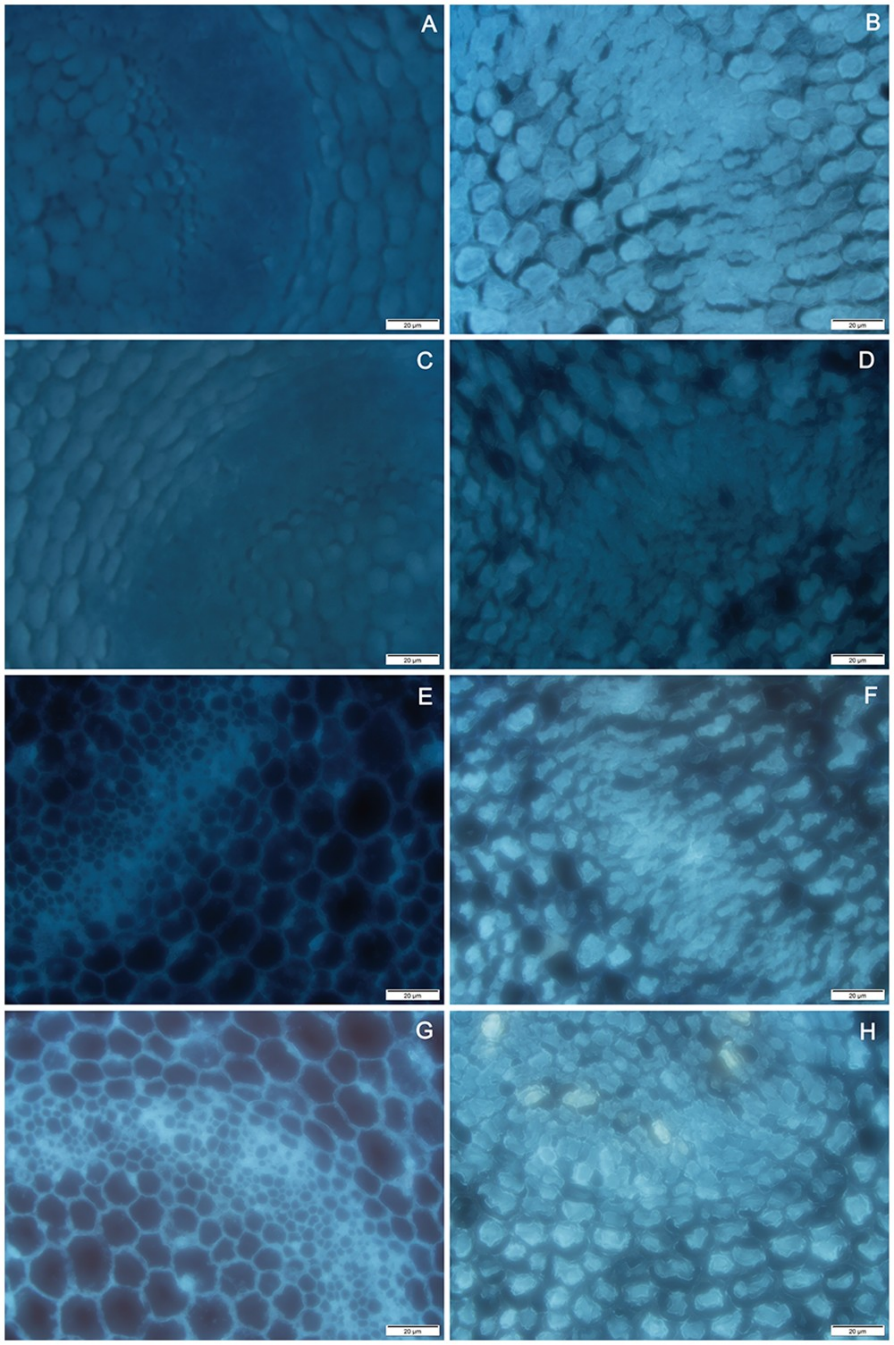
Fluorescent in situ localization of cytochrome C from mung bean hypocotyls in different preimbibition and dehydration states. A, C, E and G show hypocotyl tissue at 3, 6, 18 and 24 h of preimbibition, respectively. B, D, F and H show hypocotyl tissue with 3, 6, 18 and 24 h of preimbibition after 24 h of dehydration, respectively.

During normal imbibition, cytochrome C distributes steadily in mitochondria and does not leak into the cytoplasm. The effect of dehydration on the distribution of cytochrome C was obvious, especially the increase of cytoplasmic staining in the hypocotyl tissue after 18 h and 24 h preimbibition, which indicates that dehydration would lead to the release of cytochrome C into the cytoplasm (Fig 12 and S13 Supporting Information). This is consistent with the timing of mitochondrial membrane potential reversal.

### Dehydration increases the cytoplasmic Ca^2+^ concentration of protoplasts

**Fig 13:**
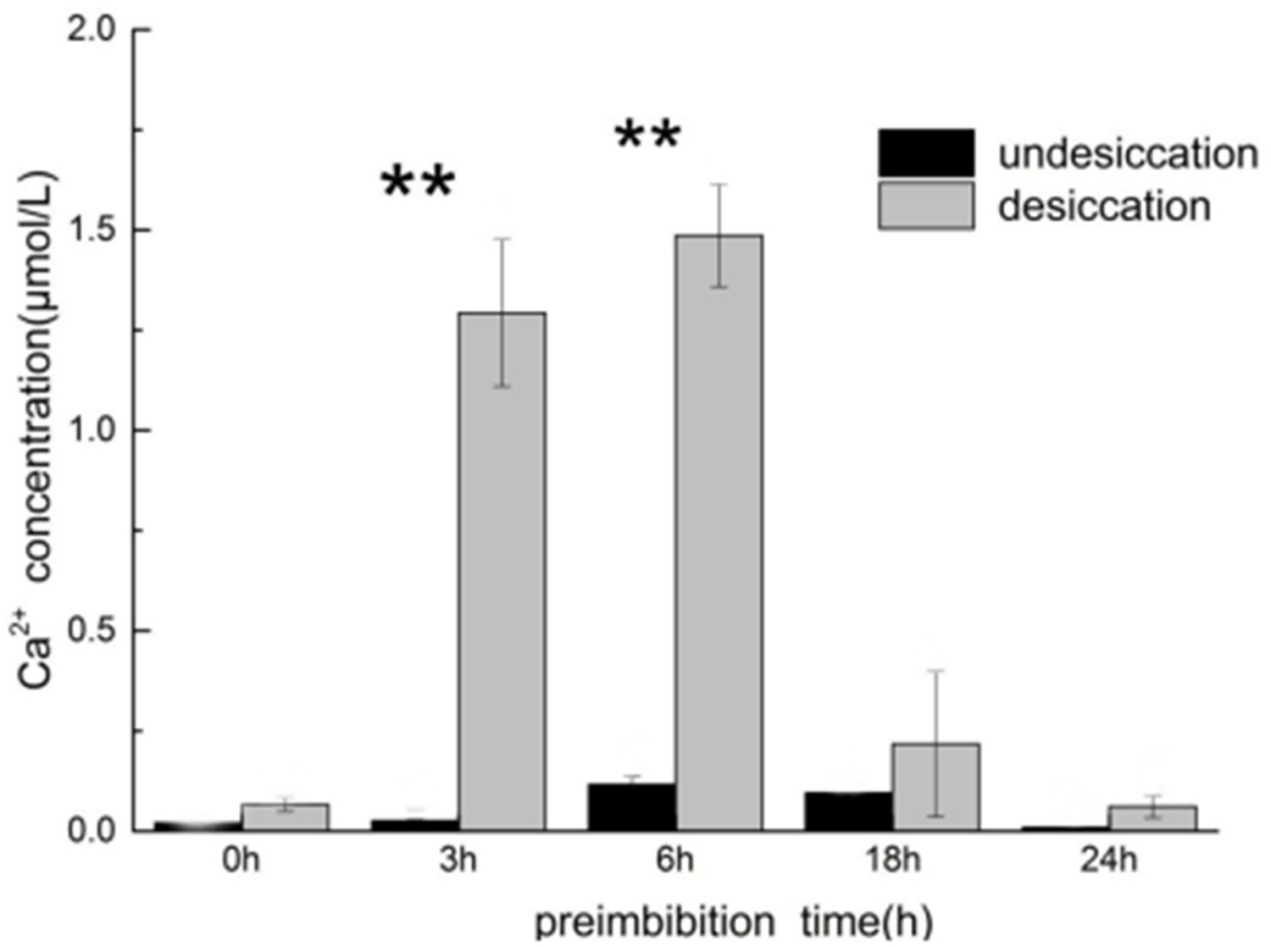
Concentration of cytoplasmic Ca^2+^ in mung bean hypocotyl tissue in different preimbibition and dehydration states. ** *P* < 0.01.

In the normal imbibition process, the cytoplasmic Ca^2+^ concentration increased slightly first and then decreased. In the early and middle stages of preimbibition (3–6 h), dehydration significantly increased the cytoplasmic Ca^2+^ concentration of protoplasts to 1.25–1.5 μmol/L, which was 5–7 times higher than that of resting cytoplasmic Ca^2+^ in animal cells, but there was no significant difference between the middle and late stages of preimbibition. The change in cytoplasmic Ca^2+^ concentration preceded PCD events, such as mitochondrial membrane potential reversal and cytochrome C release (Fig 13 and S14 Supporting Information).

## Discussion

Evans blue staining and TTC staining are important methods for identifying dead/surviving cells. Our results show that dehydration leads to cell death and that the degree of cell death increases with the prolongation of preimbibition time. It can be inferred that the damage of dehydration stress causes a decrease in seed viability, mainly due to the death of the meristem (Figs 1 and 2 and S1 and S2 Supporting Information). PCD or apoptotic cells have unique morphological characteristics [15], showing cytoskeletal destruction, cell volume shrinkage and increased density. Microstructural changes, especially subcellular changes, have always been the gold standard for determining the existence of PCD events. Therefore, this experiment is aimed at the microscopic results in cells and the changes in subcellular structure. During the experiment, it was observed that dehydration after preimbibition could induce the optical morphology of mung bean hypocotyl cells to show obvious PCD characteristics, such as abnormal nucleus morphology and increased vacuolation (Fig 3 and S3 Supporting Information). Nucleolus and nucleolus deformities, nuclear condensation, nuclear margination, nuclear envelope rupture, nuclear degradation and other PCD characteristics were also observed by electron microscopy (Figs 4 and 5 and S4 and S5 Supporting Information). The changes in mitochondrial morphology indicated that the number of ridges decreased at the middle stage of 6 h preimbibition, and the mitochondria ruptured and the outer membrane boundary disappeared in preimbibition after 24 h (Fig 6 and S6 Supporting Information).

DNA degradation occurs in the process of PCD, especially in the breakage of autokaryosomes [16]. DNA degradation characteristics of plant PCD are not as typical as those of animal cells [17]. DNA electrophoresis, nucleosome ELISA and TUNEL were employed to detect the integrity of cell DNA. The results showed that after dehydration, with the extension of imbibition time, the degree of DNA degradation increased (S1 Fig and S7 Supporting Information), the number of nucleosomes increased significantly (Fig 7 and S9 Supporting Information), the activity of total nuclease increased (Fig 8 and S9 Supporting Information), and the integrity of DNA was seriously damaged.

Modern studies have shown that during animal PCD, cells are characterized by DNA marginalization (condensation) on the nuclear membrane and are divided into nucleosome-sized lengths. The nucleus and cytoplasm are broken down into vesicles, and macrophages clear the body in vivo. PCD in plants is similar to that of animals. In view of the changes in cell morphology, caspase activity and DNA integrity, it is suggested that PCD should exist as part of the dehydration response and in the hypocotyl tissue cells after dehydration in the late preimbibition stage.

In the process of apoptosis of animal cells, caspase is the core executor; caspase-9 is the main inducing factor, and caspase-3 is the executor. Caspase-related or similar enzymes can be used as important markers of the occurrence of PCD in plants. The results show that dehydration significantly increased the activity of caspase-9 and caspase-3. At the late stage of preimbibition, caspase activities changed dramatically (Figs 9 and 10 and S10 Supporting Information), indicating that dehydration at the late stage of preimbibition could lead to PCD in hypocotyl cells.

Balk et al. [18] confirmed the relationship between cytochrome C release and PCD in mitochondrial mutants of PET1-CMS in sunflower. In this study, cytochrome C was distributed in the cytoplasm from 18 h to 24 h after imbibition (Fig 12 and S13 Supporting Information) and then combined with the reversal of mitochondrial membrane potential (Fig 11 and S11 Supporting Information) and the morphological changes of mitochondria (Fig 6 and S6 Supporting Information). Thus, it can be inferred that the mitochondrial apoptosis pathway may be the main form of dehydration-induced PCD. PCD-related factors released by mitochondria enter the cytoplasm in two ways: one is the loss of mitochondrial membrane integrity, and the other is the release through the mitochondrial permeability transition (PT) pore [2]. The results show that changes in the mitochondrial ridge caused an imbalance in respiratory electron transport from the middle stage of preimbibition, which could induce changes in the mitochondrial membrane potential, initiate PT channels and release apoptosis-inducing factors into the cytoplasm. The results of this experiment show that the mitochondrial membrane potential inversion, mitochondrial morphological changes, and cytochrome C distribution changes proved the above inference.

The increase in cytoplasmic Ca^2+^ concentration is a precursor signal event of PCD. For example, Pennel and Lamb [19] considered that the elevation of Ca^2+^ concentration induced by ethylene is an important component of PCD signal transduction in plants. We observed that the increase in cytoplasmic Ca^2+^ concentration occurred after dehydration in the early and middle stages of preimbibition, but there was no significant change in the late stage of preimbibition when PCD occurred (Fig 13 and S14 Supporting Information). The PT channel is the receptor with which plant mitochondria obtain the upstream signals of PCD and sense ATP, reactive oxygen species, Ca^2+^ concentration and receptor signals [20]. The important target downstream of the Ca^2+^ enhancement signal is the opening of the mitochondrial PT pore and the release of cytochrome C.

## Conclusions

The results of mitochondrial membrane potential and fluorescent in situ localization of cytochrome C, combined with the results of morphological changes in mitochondria, suggested that mitochondria are an important inducer and transmitter involved in PCD in the dehydration response process. That is, the mitochondrial apoptosis pathway can be considered the main form of dehydration-induced PCD. The whole process can be divided into dehydration induction, transfer changes in Ca^2+^ and mitochondrial respiratory electron, ROS and Ca^2+^-induced mitochondrial membrane potential reversal, and cytochrome C release. The hypothesis of mitochondrial pathway PCD as the reason for loss of desiccation tolerance can be confirmed by classical morphological observation and by physiological and biochemical analysis (S4 Fig).

## Supporting information

S1 Fig Electrophoresis of total DNA from mung bean hypocotyls in different preimbibition and dehydration states. A shows electrophoresis of total DNA at 0, 3, 6, 18 and 24 h of preimbibition, while B shows electrophoresis of total DNA at 0, 3, 6, 18 and 24 h preimbibition after 24 h of dehydration.
(TIF)

S2 Fig TUNEL in mung bean hypocotyls in different preimbibition and dehydration states. A, C, E and G show mung bean hypocotyl tissue at 3, 6, 18 and 24 h of preimbibition, respectively. B, D, F and H show the transverse section of the hypocotyl at 3, 6, 18 and 24 h of preimbibition after 24 h of dehydration, respectively.
(TIF)

S3 Fig Flow cytometer quantity of JC-1 staining in protoplasts from mung bean hypocotyls in different preimbibition and dehydration states. A, C, E, G and I represent the measurement of protoplasts at 3, 6, 18 and 24 h of preimbibition, respectively. B, D, F, H and J represent the measurement of protoplasts at 0, 3, 6, 18 and 24 h of preimbibition after 24 h of dehydration, respectively.
(TIF)

S4 Fig Illustration of the mechanism of PCD induced by dehydration in mung bean hypocotyls. (TIF)

S1 Supporting Information Raw images of Evans blue staining of mung bean hypocotyls in different preimbibition and dehydration states. (ZIP)

S2 Supporting Information Raw images of TTC staining of mung bean hypocotyls in different preimbibition and dehydration states. (ZIP)

S3 Supporting Information Raw images of Toluidine blue staining of mung bean hypocotyls in different preimbibition and dehydration states. (ZIP)

S4 Supporting Information Raw images of subcellular structure of meristematic cells from mung bean hypocotyls in different preimbibition and dehydration states. (ZIP)

S5 Supporting Information Raw images of ultrastructure of meristematic cell nuclei in different preimbibition and dehydration states. (ZIP)

S6 Supporting Information Raw images of ultrastructure of meristematic cell mitochondria in different preimbibition and dehydration states. (ZIP)

S7 Supporting Information Raw images of electrophoresis of total DNA from mung bean hypocotyls in different preimbibition and dehydration states. (ZIP)

S8 Supporting Information Raw images of TUNEL in mung bean hypocotyls in different preimbibition and dehydration states. (ZIP)

S9 Supporting Information Individual data points for analysis of nuclease activity and nucleosome content in mung bean hypocotyls in different preimbibition and dehydration states. (OPJ)

S10 Supporting Information Individual data points for analysis of activities of caspase-3 and caspase-9 in mung bean hypocotyls in different preimbibition and dehydration states. (OPJ)

S11 Supporting Information Raw images of JC-1 staining of protoplasts from mung bean hypocotyls in different preimbibition and dehydration states. (ZIP)

S12 Supporting Information Raw images of flow cytometer quantity of JC-1 staining in protoplasts from mung bean hypocotyls in different preimbibition and dehydration states. (ZIP)

S13 Supporting Information Raw images of fluorescent in situ localization of cytochrome C from mung bean hypocotyls in different preimbibition and dehydration states. (ZIP)

S14 Supporting Information Individual data points for concentration of cytoplasmic Ca^2+^ in mung bean hypocotyl tissue in different preimbibition and dehydration states. (XLSX)
