# Supplementary figures and images for: Relationship between loss of desiccation tolerance and programmed cell death (PCD) in mung bean (Vigna radiata) seeds (part 1 of 2)
Source: PLoS One. 2019 Jul 2;14(7):e0218513. doi: 10.1371/journal.pone.0218513 (PMC6605718; doi:10.1371/journal.pone.0218513)

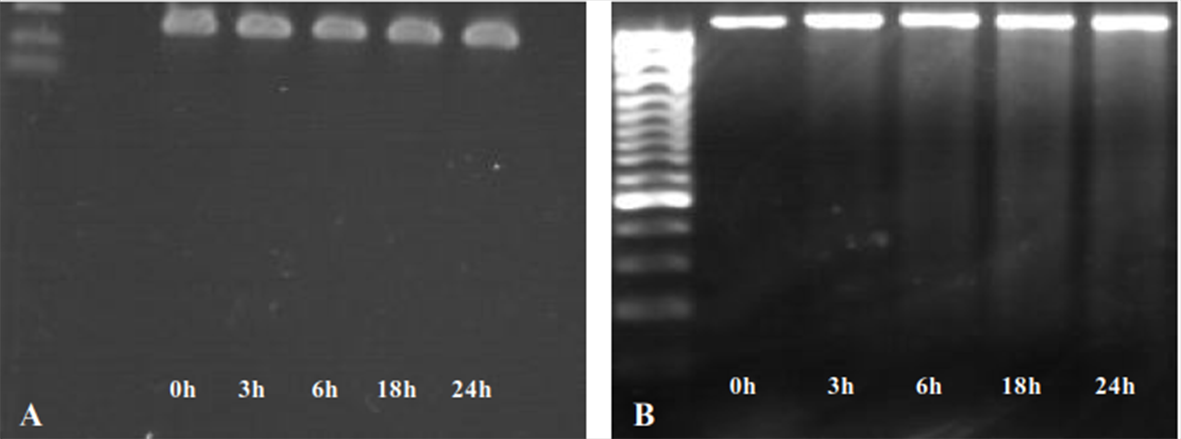

Supplement: S1 Fig — A shows electrophoresis of total DNA at 0, 3, 6, 18 and 24 h of preimbibition, while B shows electrophoresis of total DNA at 0, 3, 6, 18 and 24 h preimbibition after 24 h of dehydration. (TIF) [file pone.0218513.s001.tif]

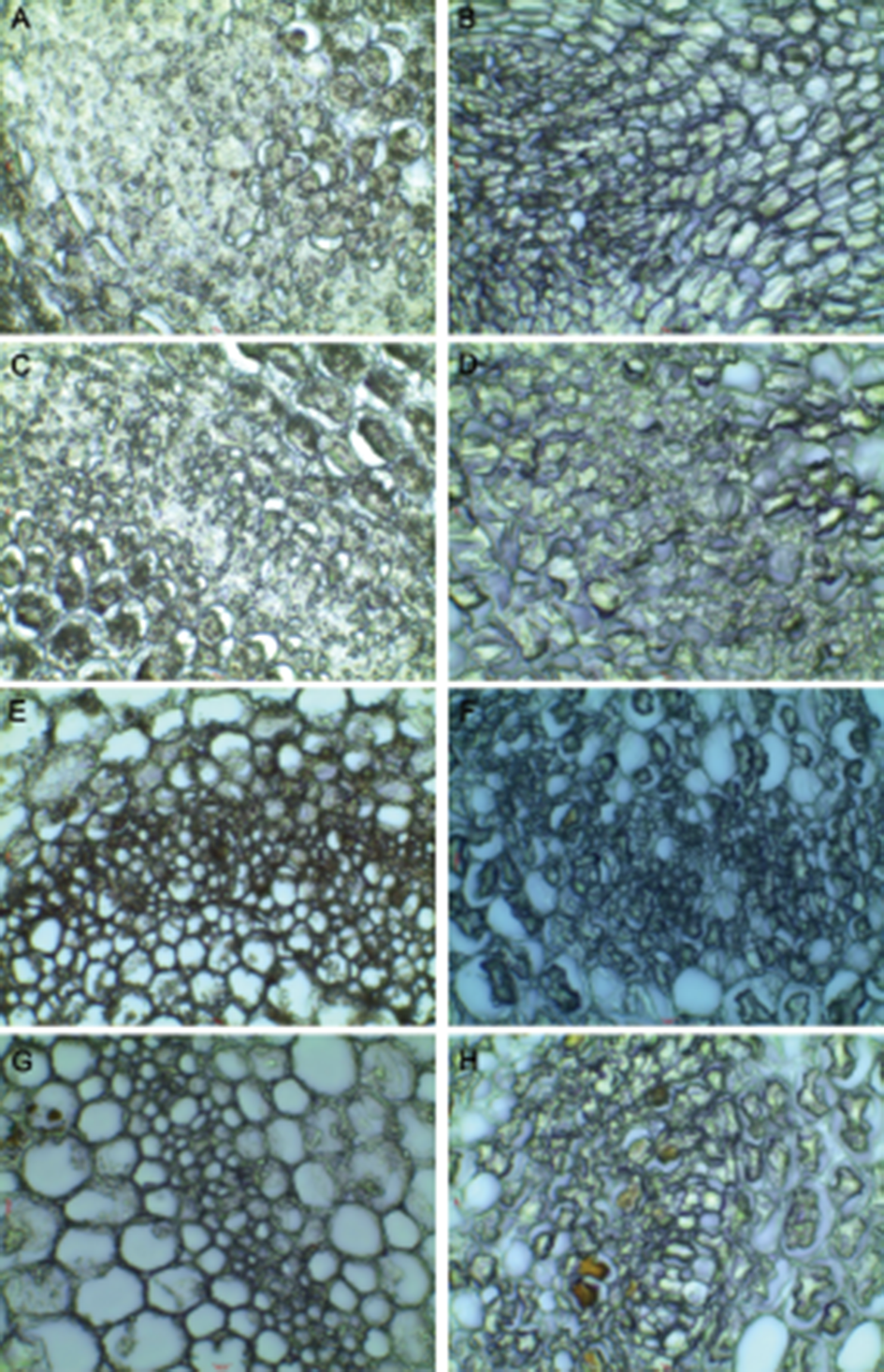

Supplement: S2 Fig — A, C, E and G show mung bean hypocotyl tissue at 3, 6, 18 and 24 h of preimbibition, respectively. B, D, F and H show the transverse section of the hypocotyl at 3, 6, 18 and 24 h of preimbibition after 24 h of dehydration, respectively. (TIF) [file pone.0218513.s002.tif]

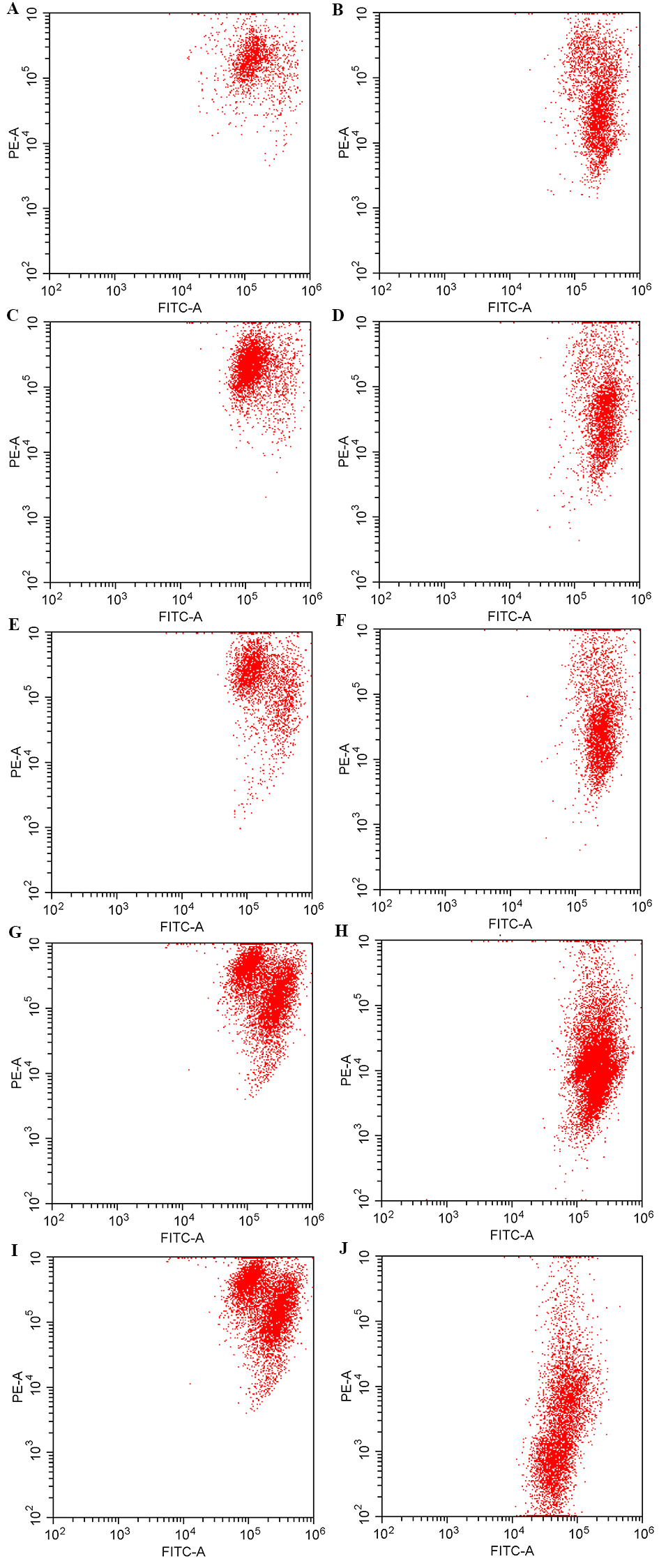

Supplement: S3 Fig — A, C, E, G and I represent the measurement of protoplasts at 3, 6, 18 and 24 h of preimbibition, respectively. B, D, F, H and J represent the measurement of protoplasts at 0, 3, 6, 18 and 24 h of preimbibition after 24 h of dehydration, respectively. (TIF) [file pone.0218513.s003.tif]

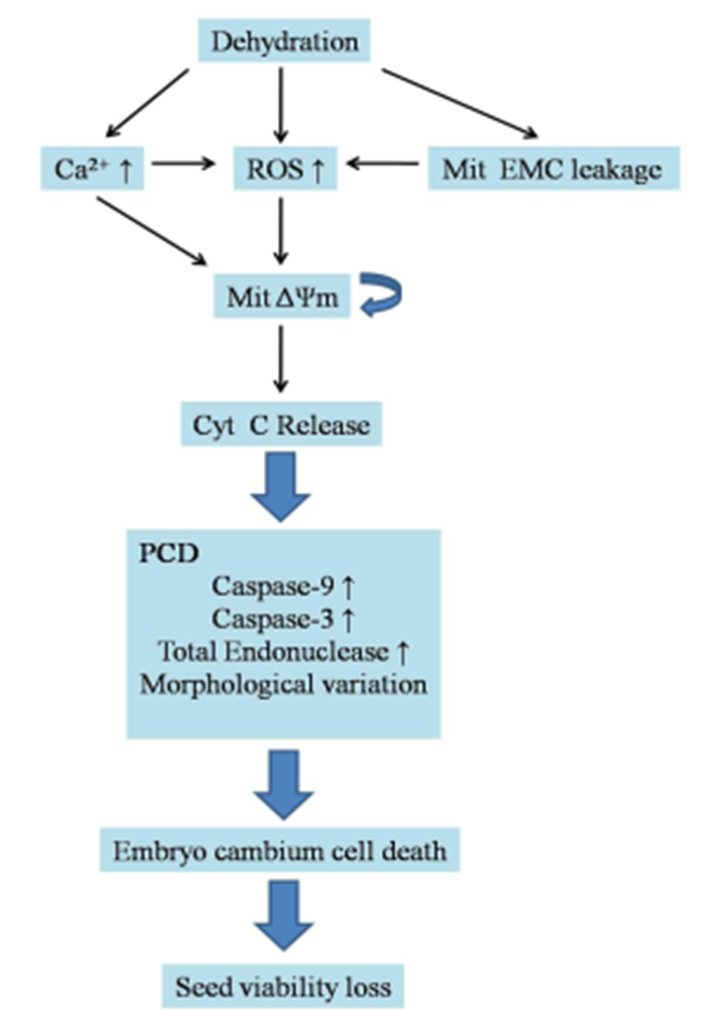

Supplement: S4 Fig — (TIF) [file pone.0218513.s004.tif]

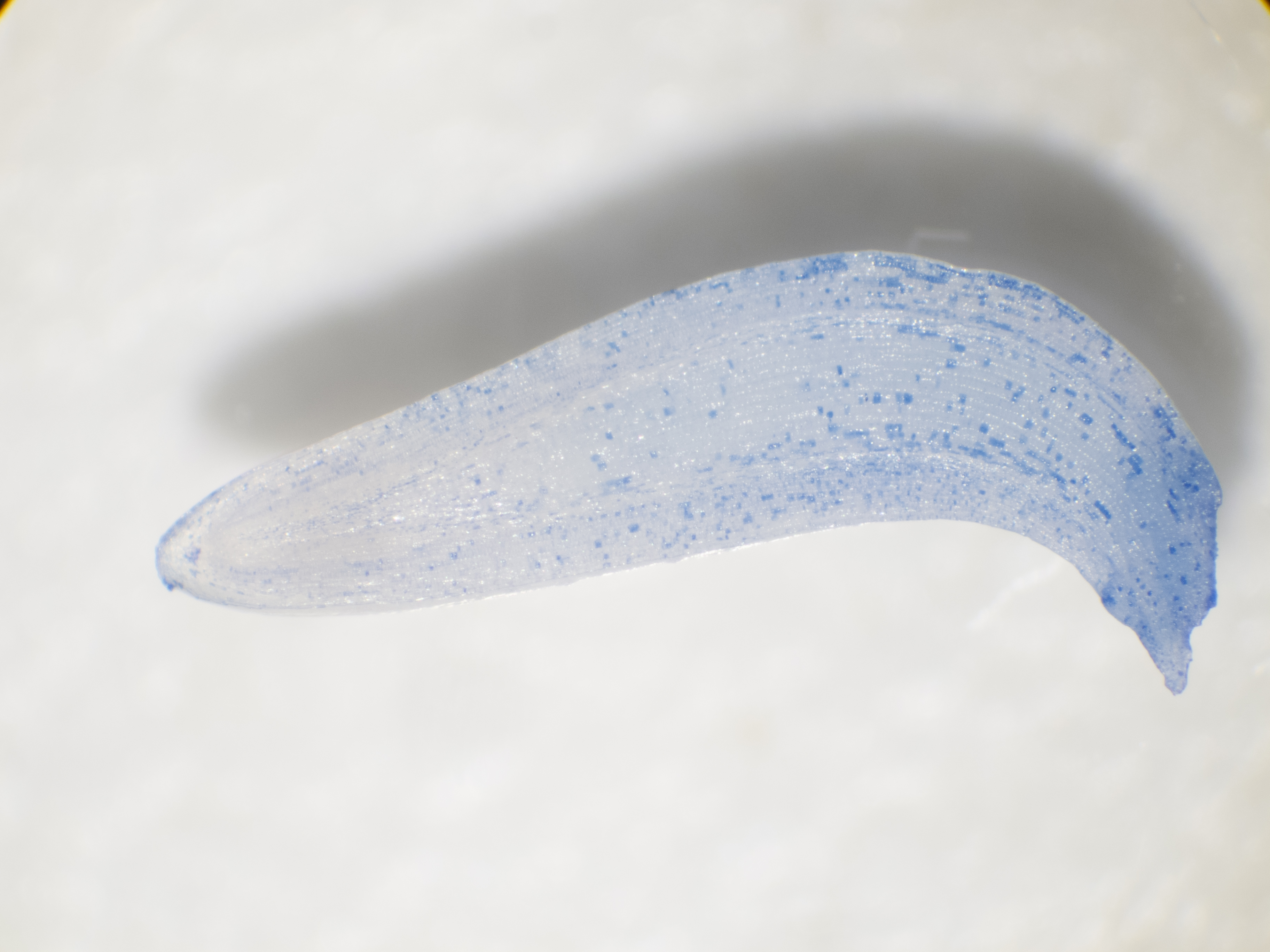

Supplement: S1 Supporting Information — (ZIP) [file pone.0218513.s005.zip › S1_Supporting Information.zip/6.jpg]

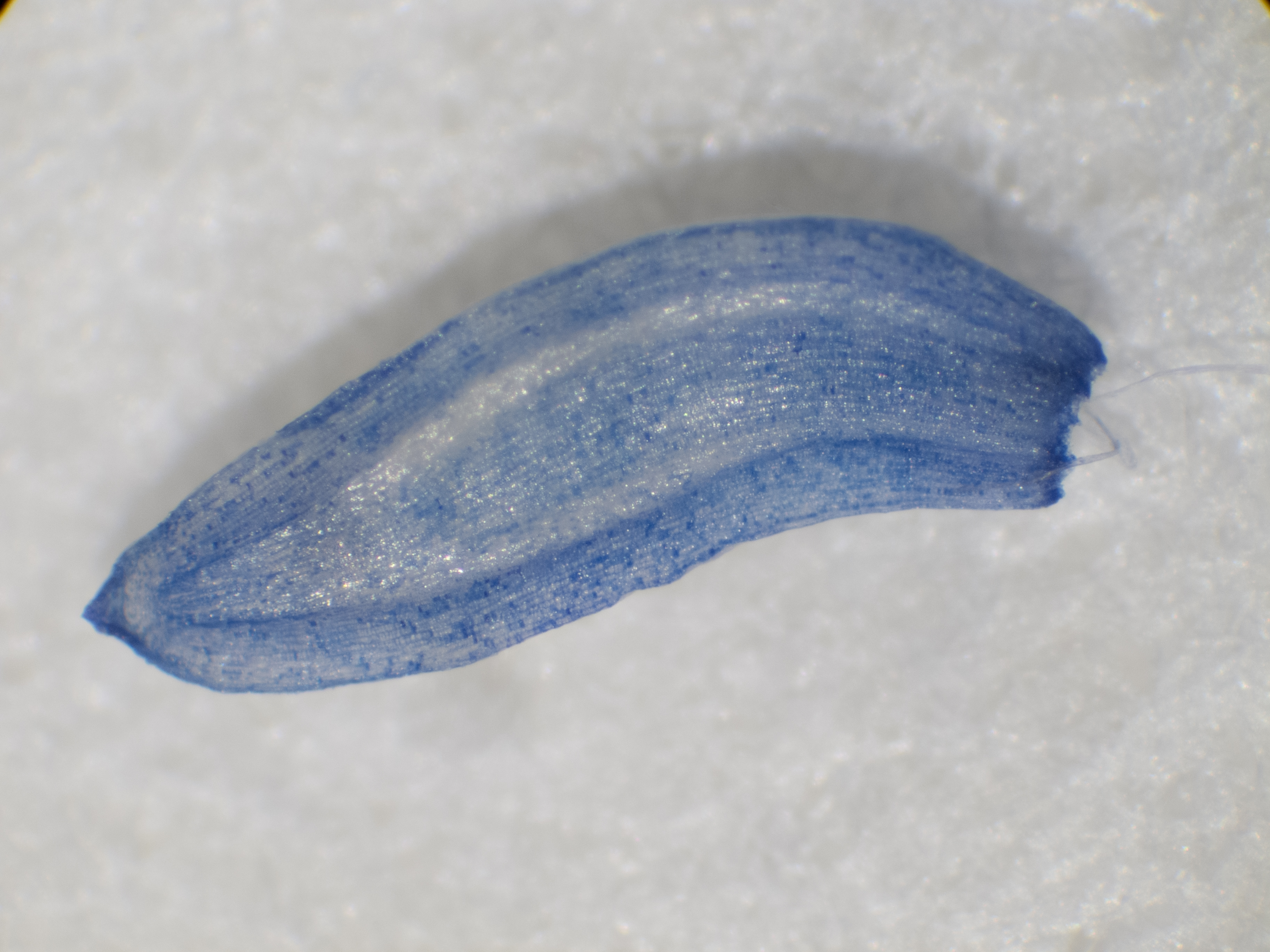

Supplement: S1 Supporting Information — (ZIP) [file pone.0218513.s005.zip › S1_Supporting Information.zip/6+24.jpg]

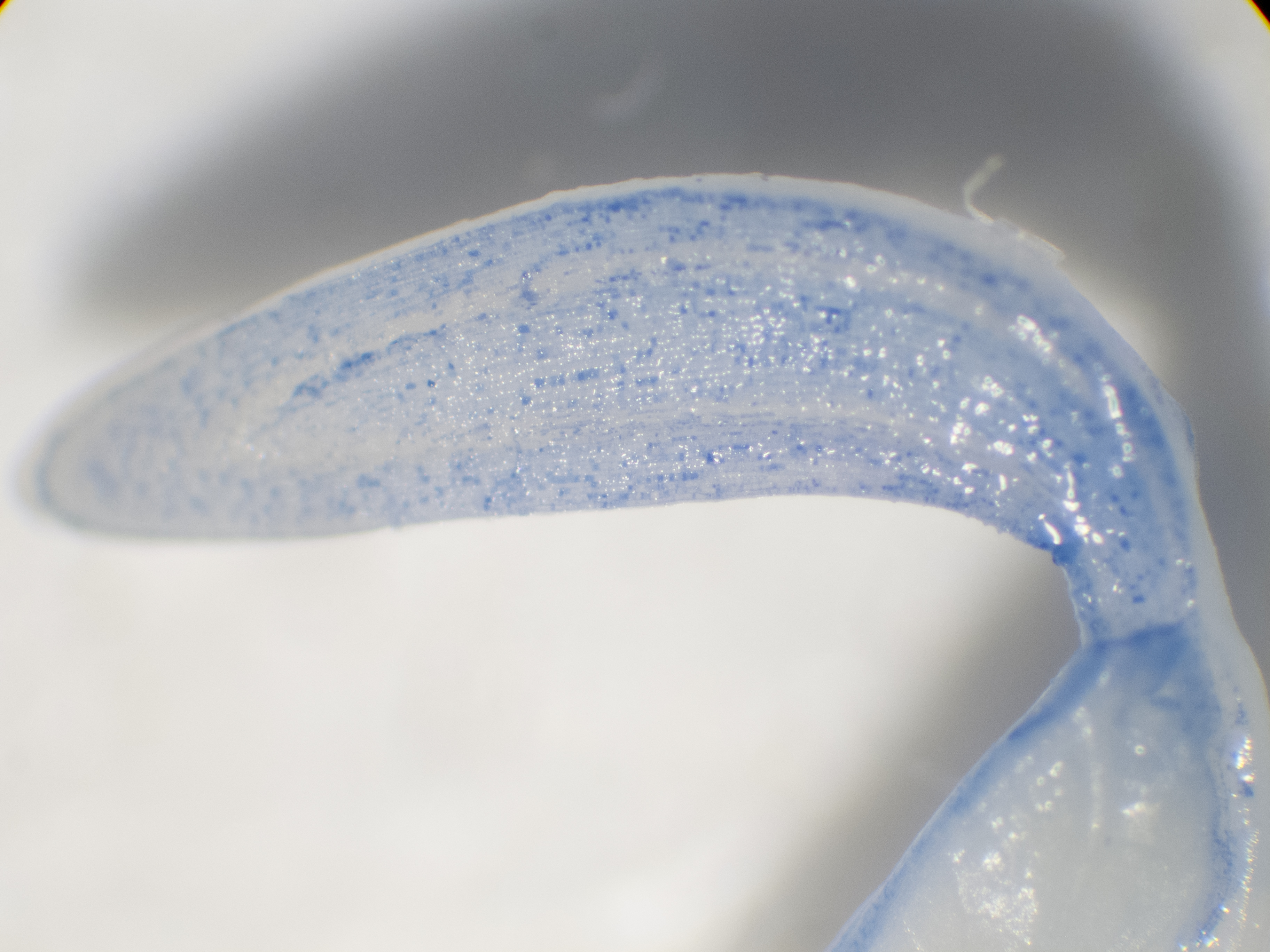

Supplement: S1 Supporting Information — (ZIP) [file pone.0218513.s005.zip › S1_Supporting Information.zip/3h(Evans Blue).jpg]

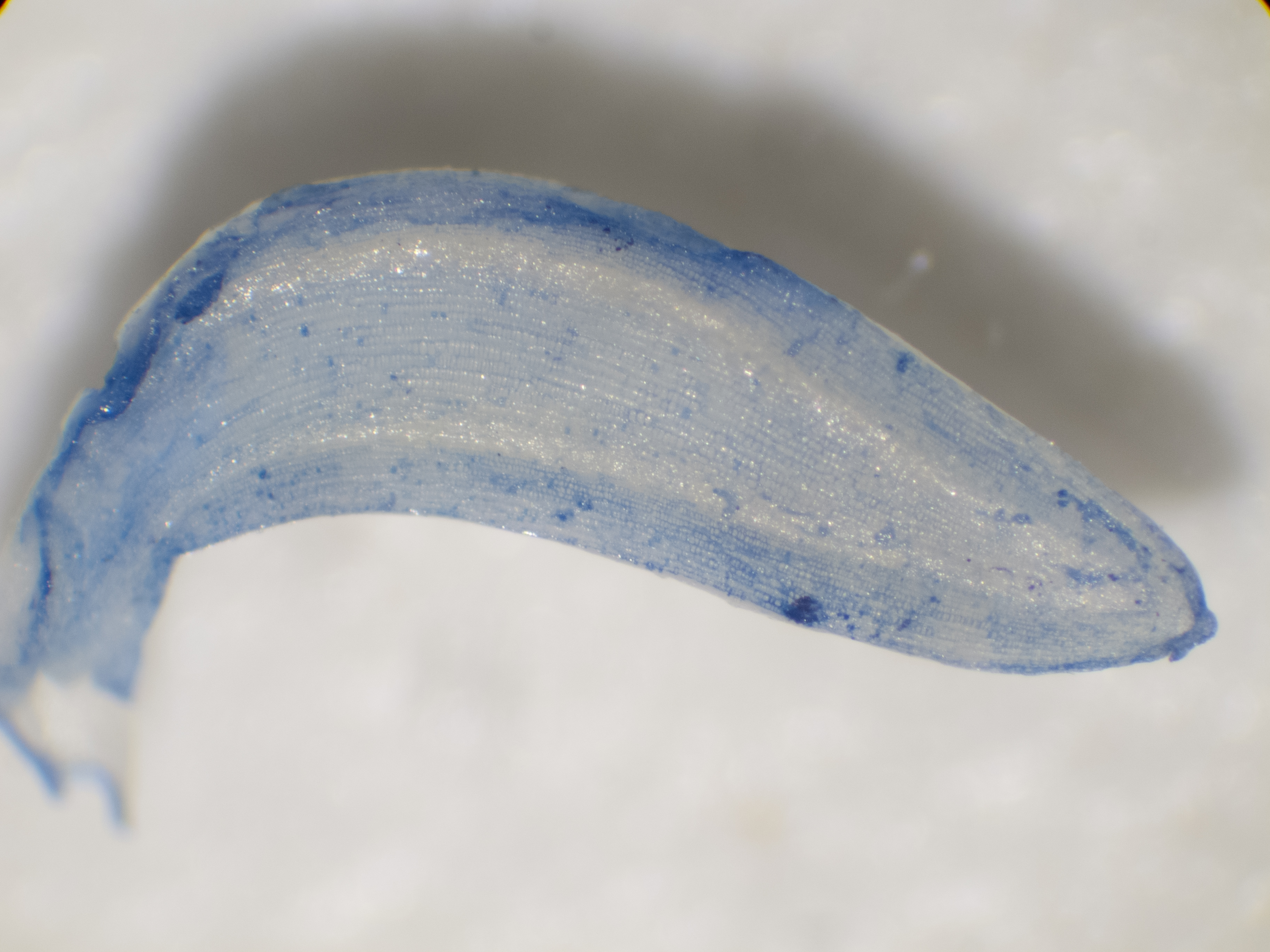

Supplement: S1 Supporting Information — (ZIP) [file pone.0218513.s005.zip › S1_Supporting Information.zip/3+24.jpg]

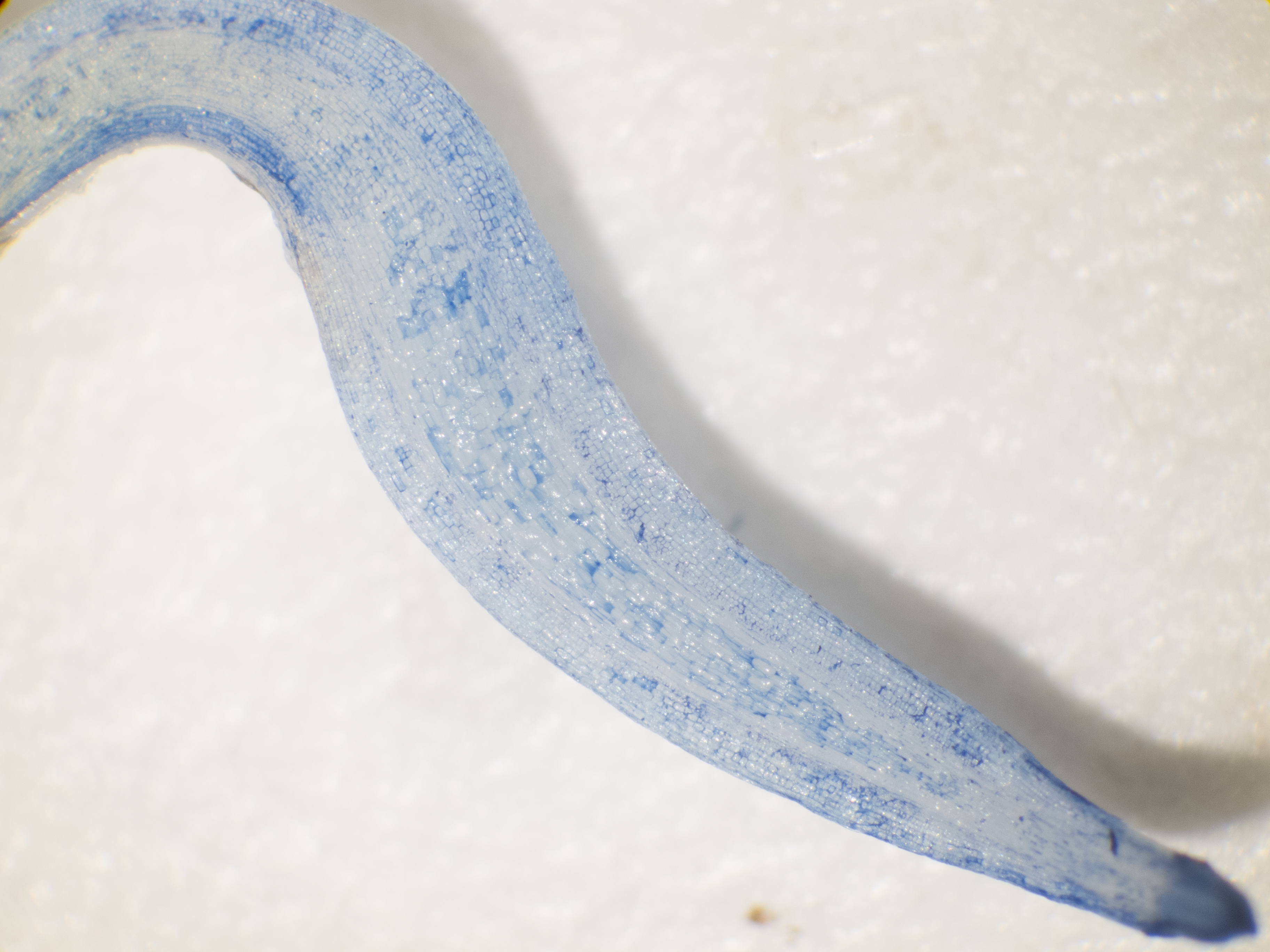

Supplement: S1 Supporting Information — (ZIP) [file pone.0218513.s005.zip › S1_Supporting Information.zip/24h.jpg]

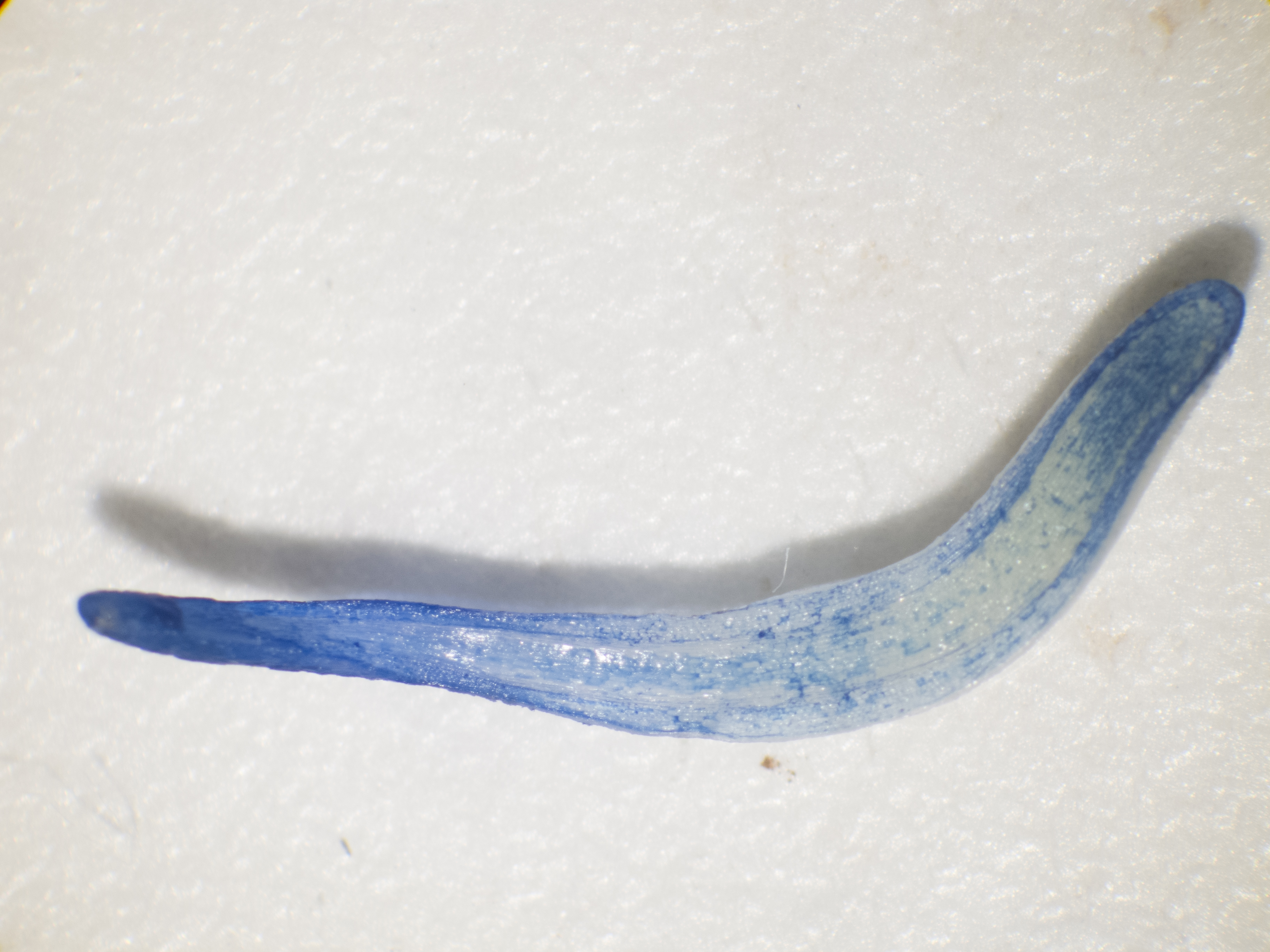

Supplement: S1 Supporting Information — (ZIP) [file pone.0218513.s005.zip › S1_Supporting Information.zip/24+24.jpg]

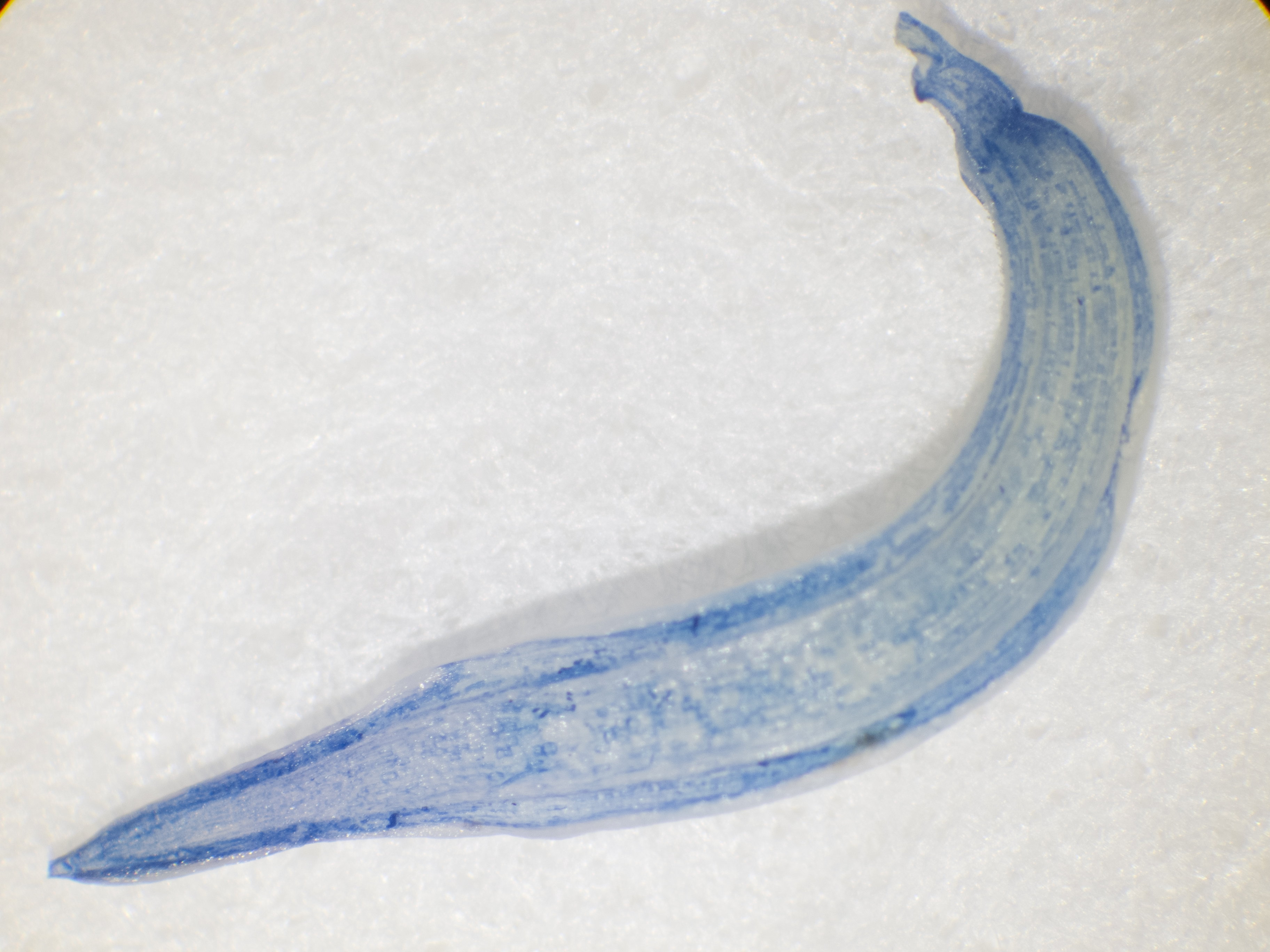

Supplement: S1 Supporting Information — (ZIP) [file pone.0218513.s005.zip › S1_Supporting Information.zip/18+24.jpg]

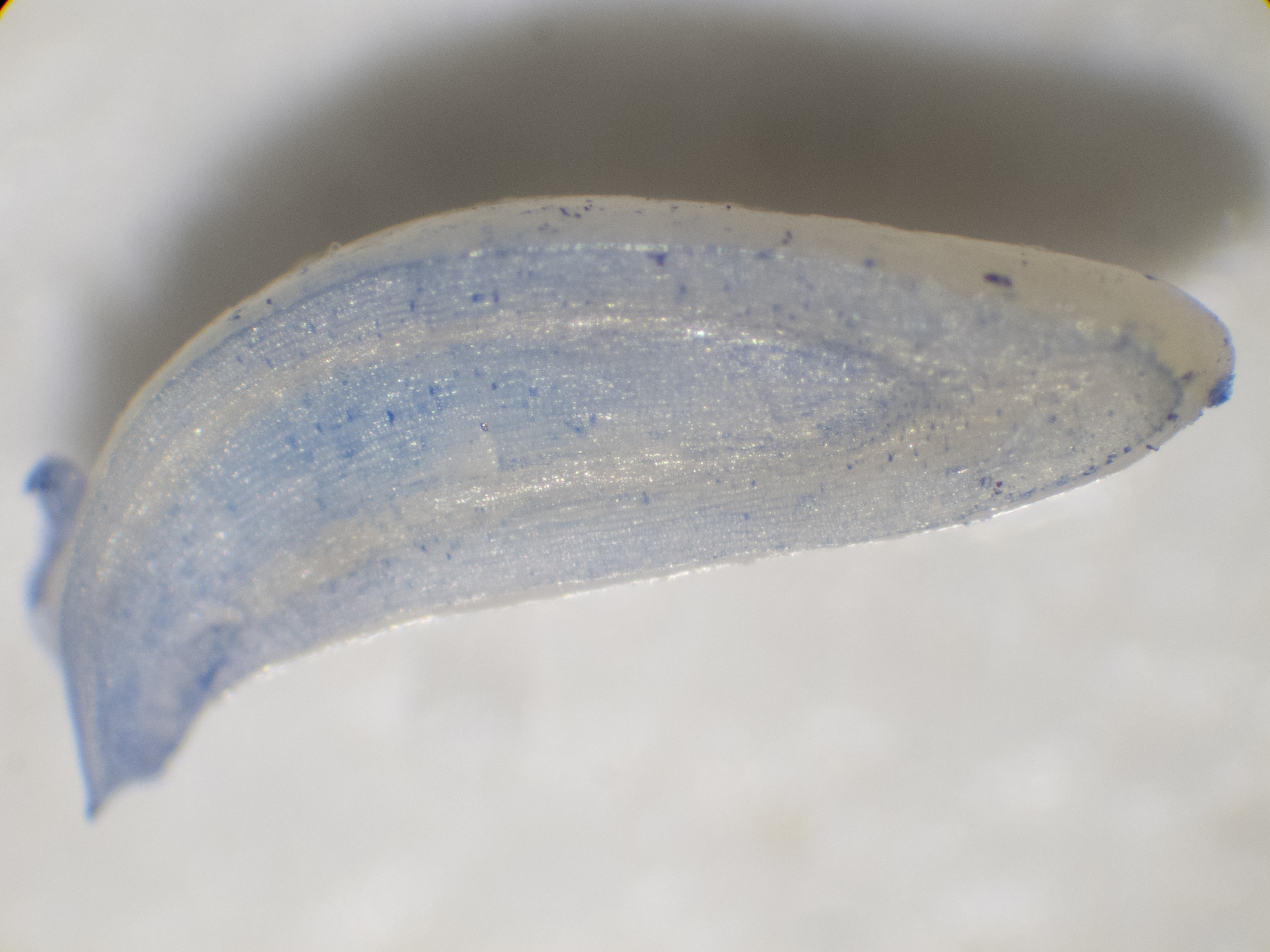

Supplement: S1 Supporting Information — (ZIP) [file pone.0218513.s005.zip › S1_Supporting Information.zip/0.jpg]

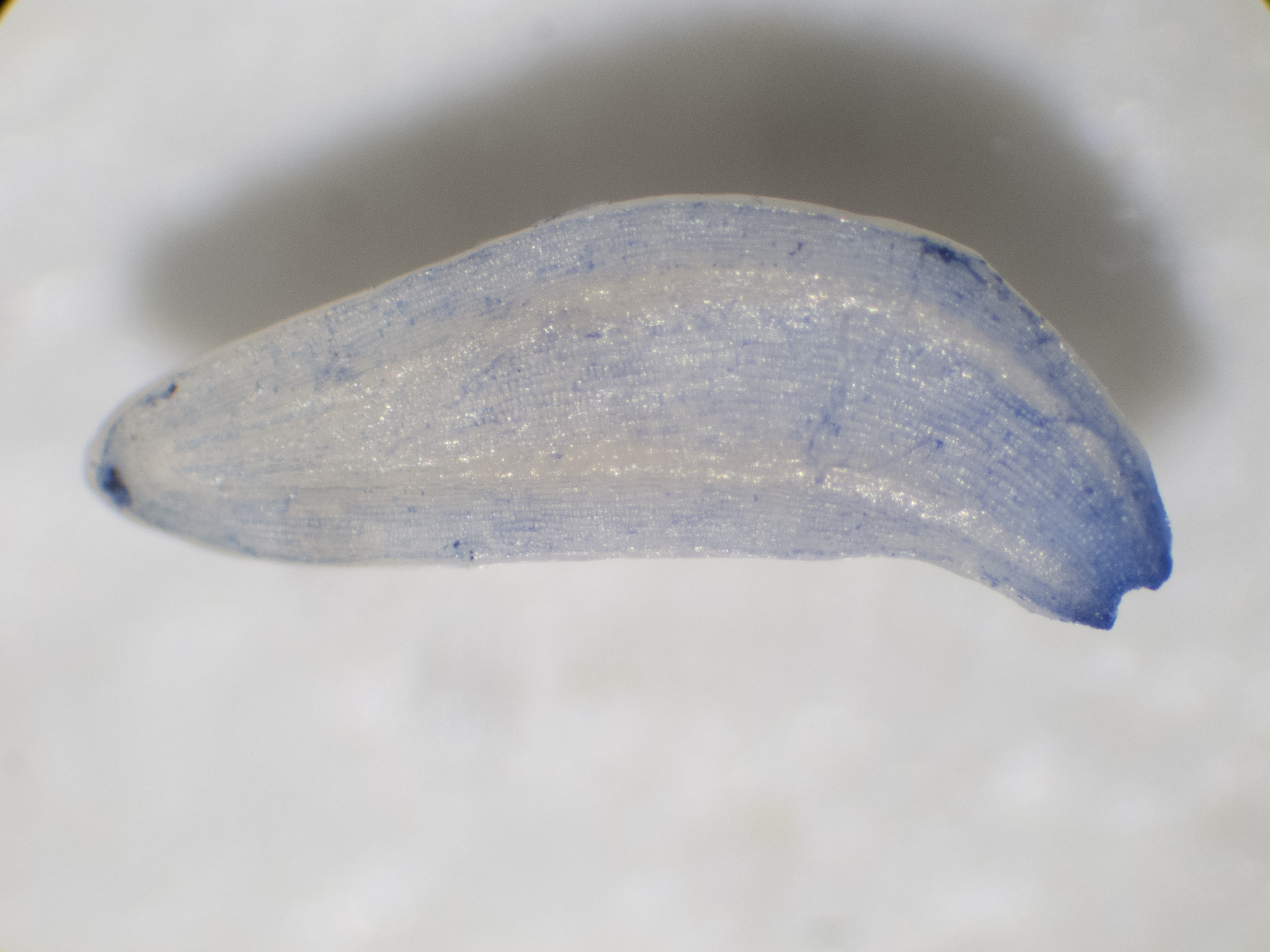

Supplement: S1 Supporting Information — (ZIP) [file pone.0218513.s005.zip › S1_Supporting Information.zip/0+24.jpg]

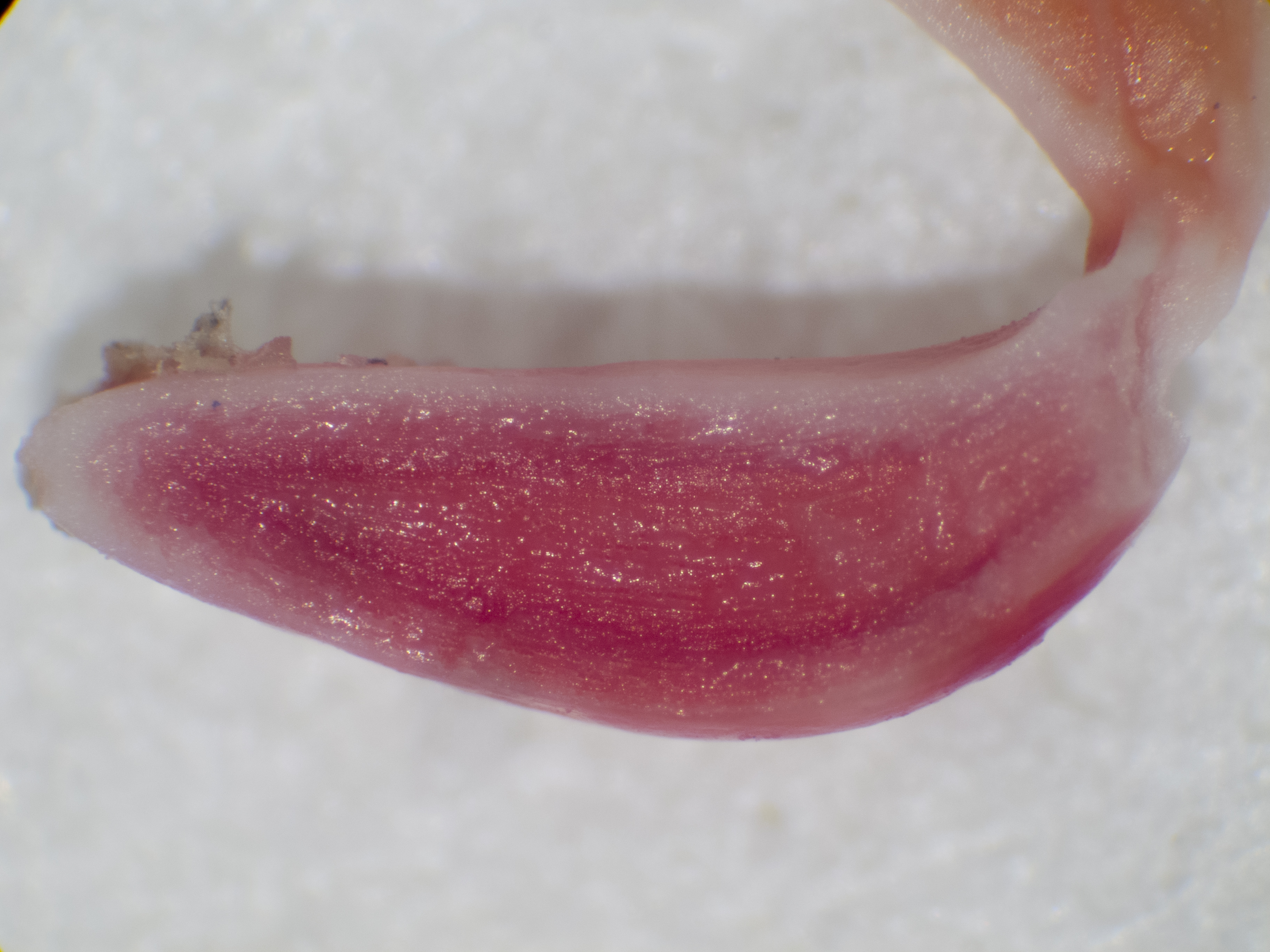

Supplement: S2 Supporting Information — (ZIP) [file pone.0218513.s006.zip › S2_Supporting Information.zip/0+24.jpg]

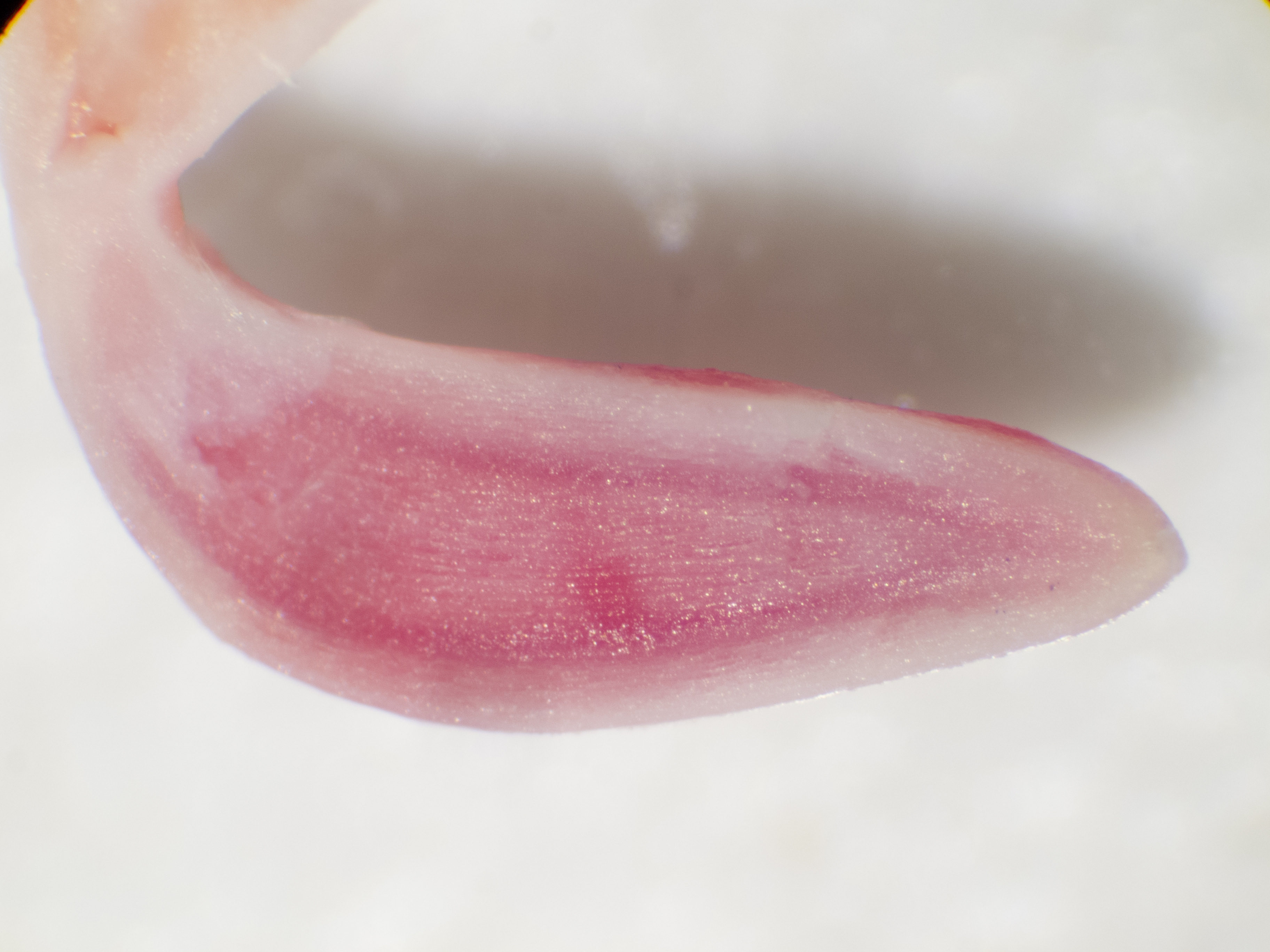

Supplement: S2 Supporting Information — (ZIP) [file pone.0218513.s006.zip › S2_Supporting Information.zip/0.jpg]

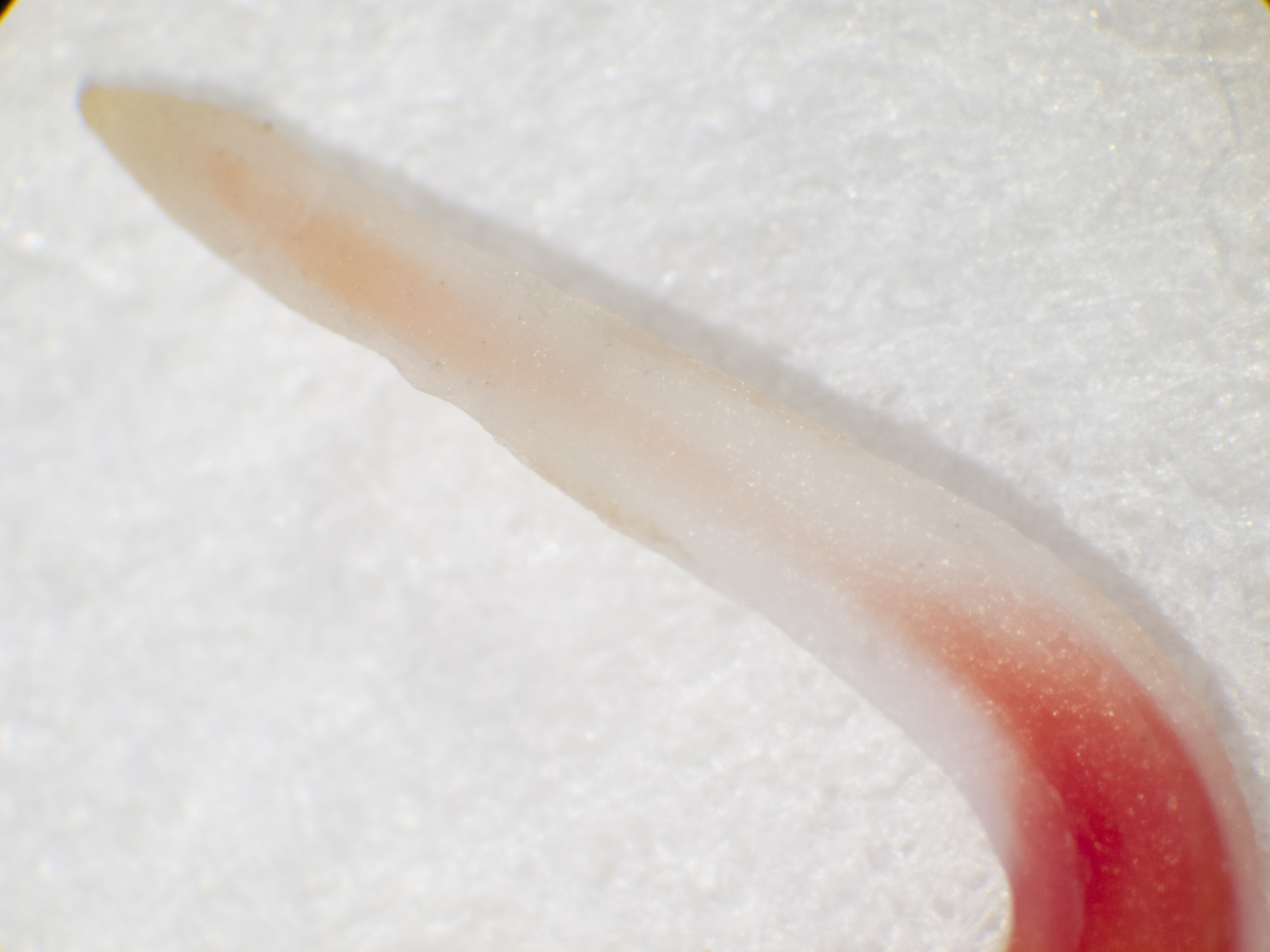

Supplement: S2 Supporting Information — (ZIP) [file pone.0218513.s006.zip › S2_Supporting Information.zip/18+24.jpg]

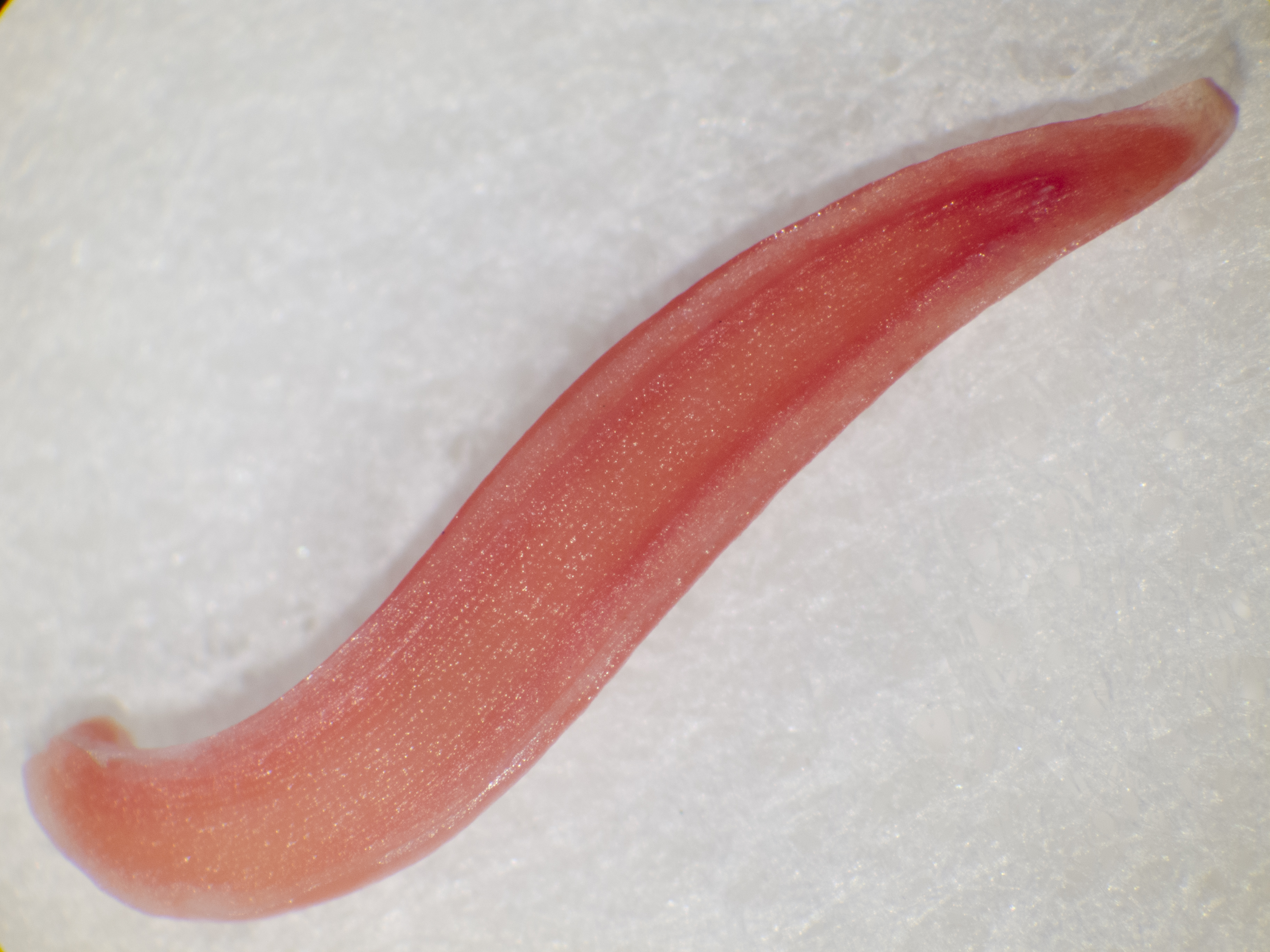

Supplement: S2 Supporting Information — (ZIP) [file pone.0218513.s006.zip › S2_Supporting Information.zip/18.jpg]

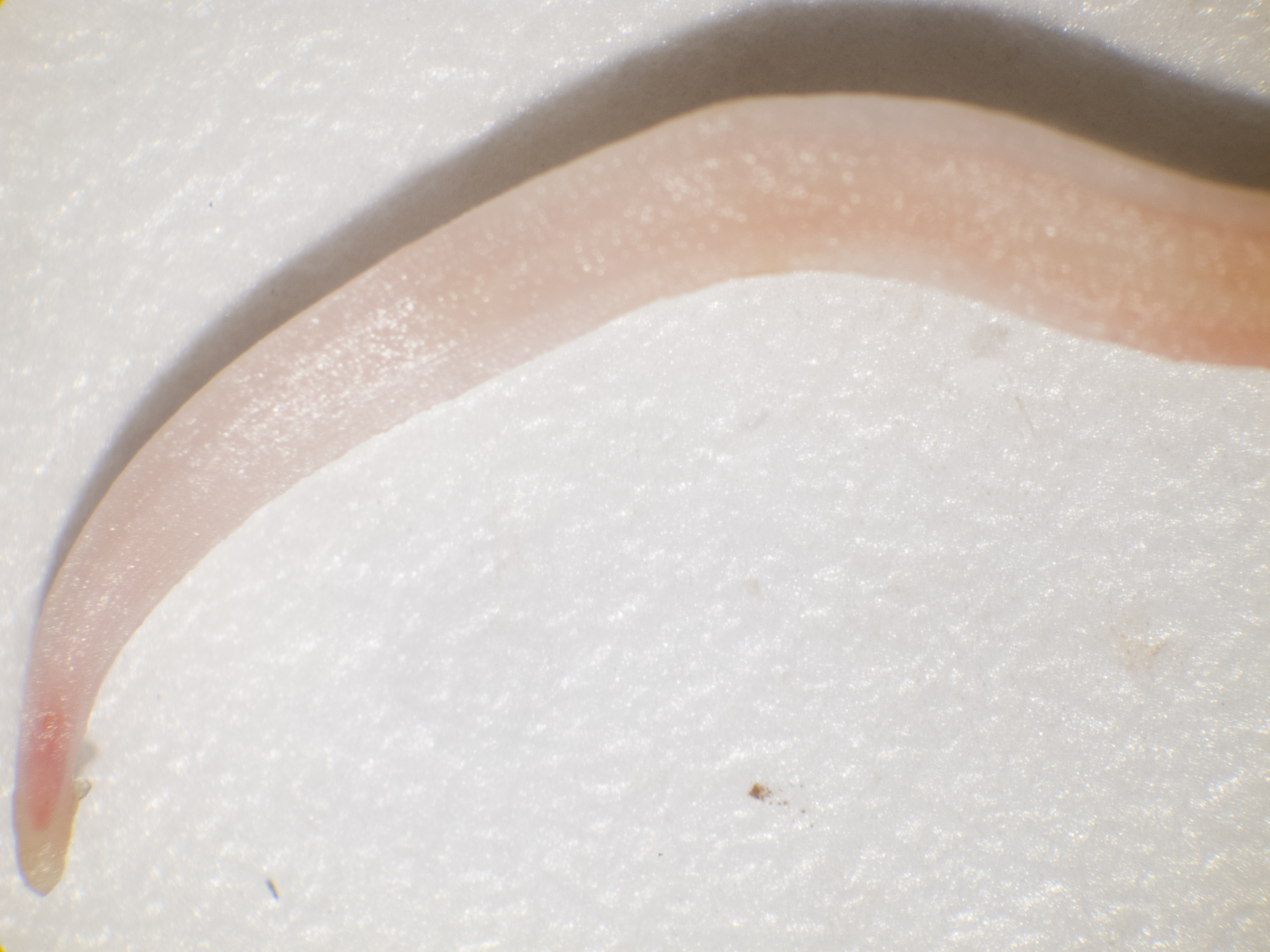

Supplement: S2 Supporting Information — (ZIP) [file pone.0218513.s006.zip › S2_Supporting Information.zip/24+24.jpg]

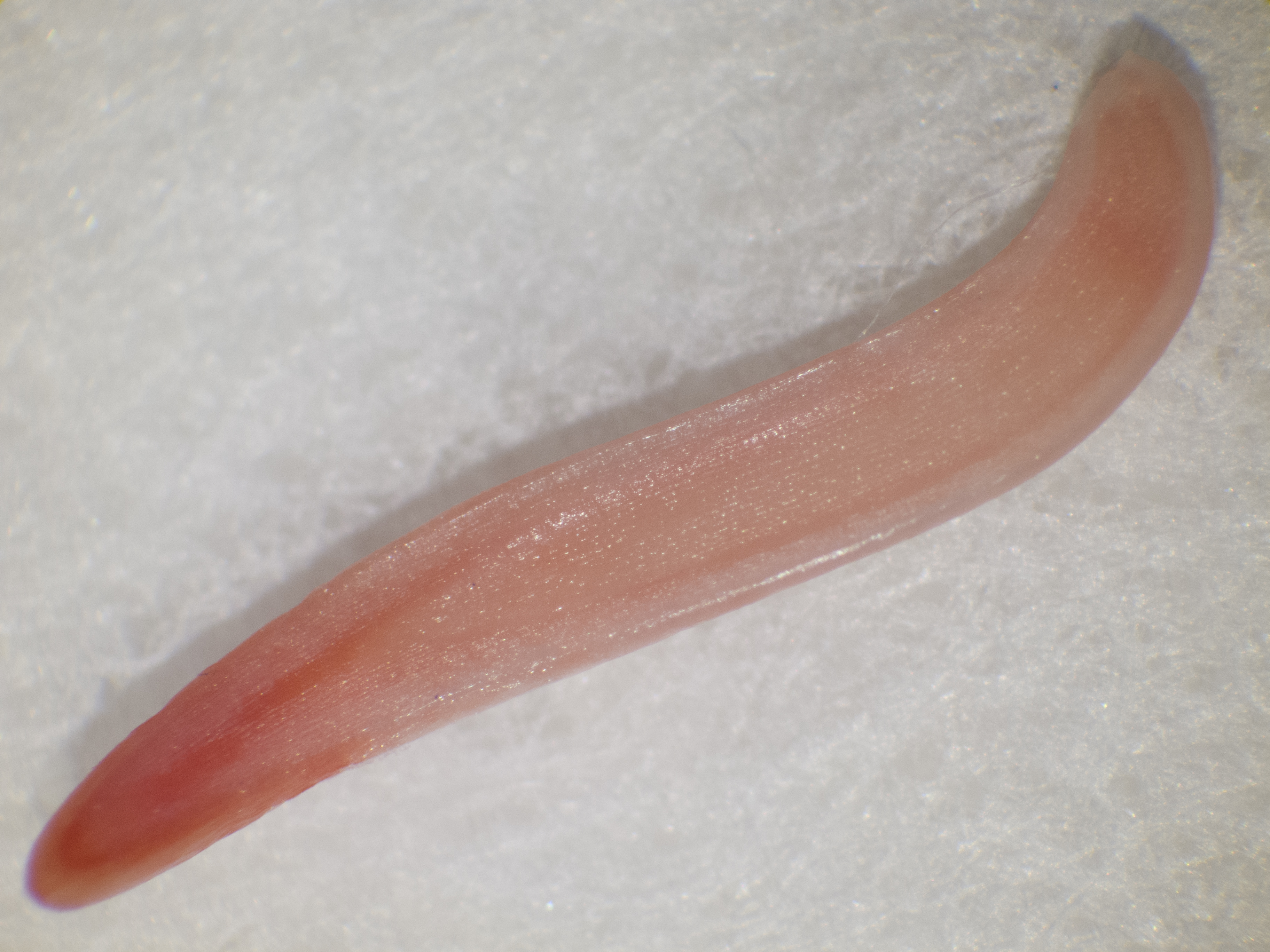

Supplement: S2 Supporting Information — (ZIP) [file pone.0218513.s006.zip › S2_Supporting Information.zip/24.jpg]

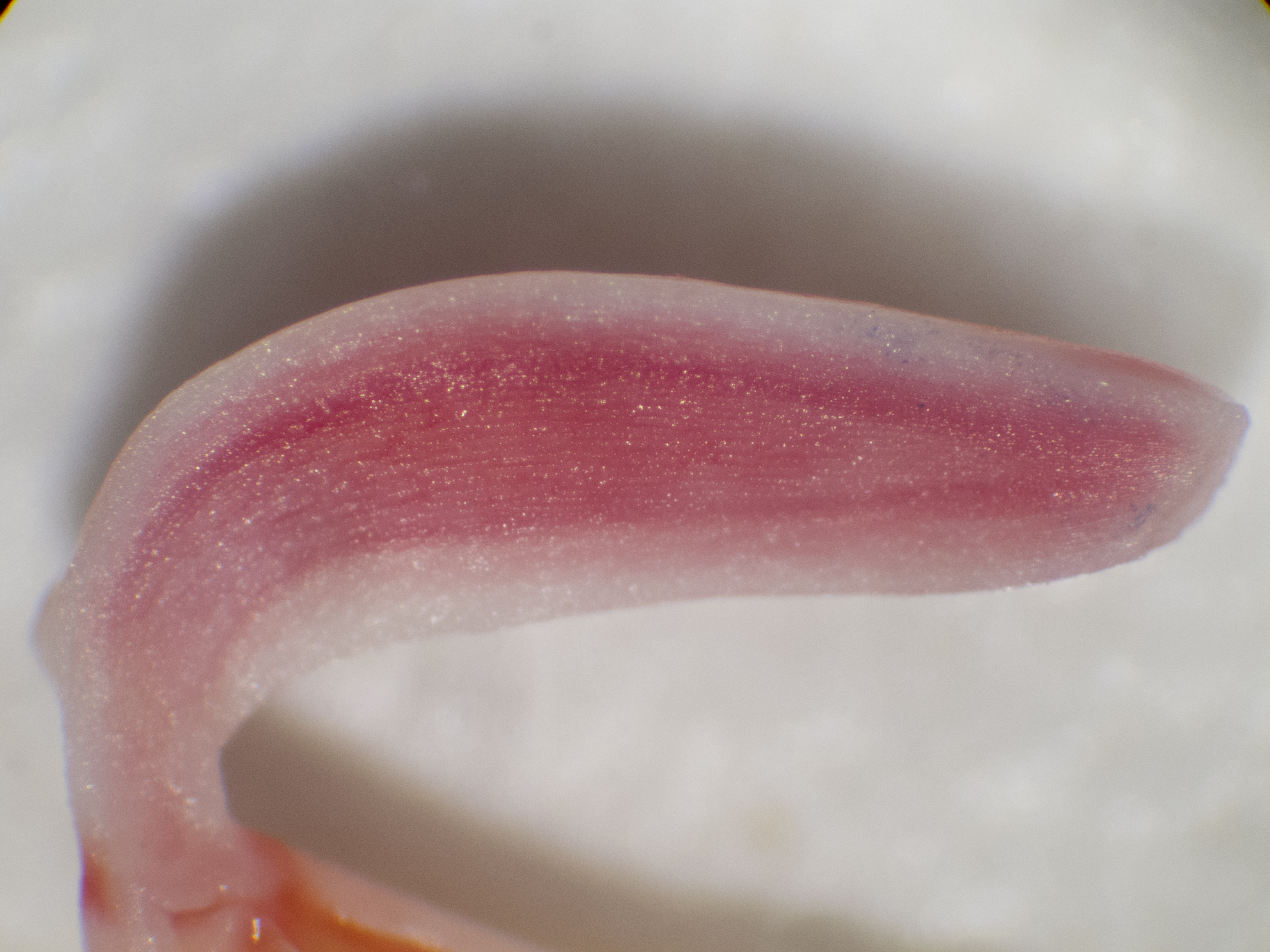

Supplement: S2 Supporting Information — (ZIP) [file pone.0218513.s006.zip › S2_Supporting Information.zip/3+24.jpg]

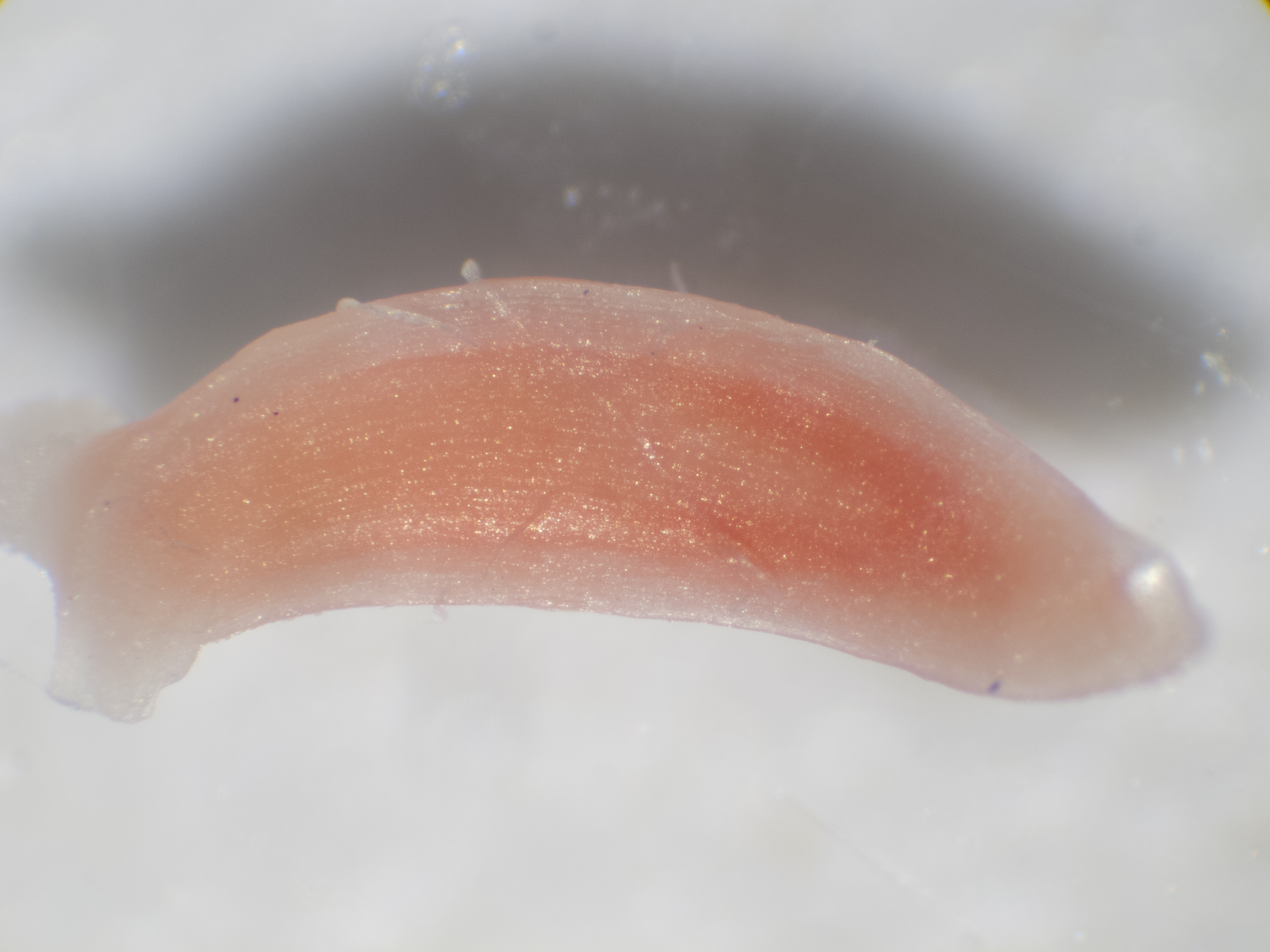

Supplement: S2 Supporting Information — (ZIP) [file pone.0218513.s006.zip › S2_Supporting Information.zip/3.jpg]

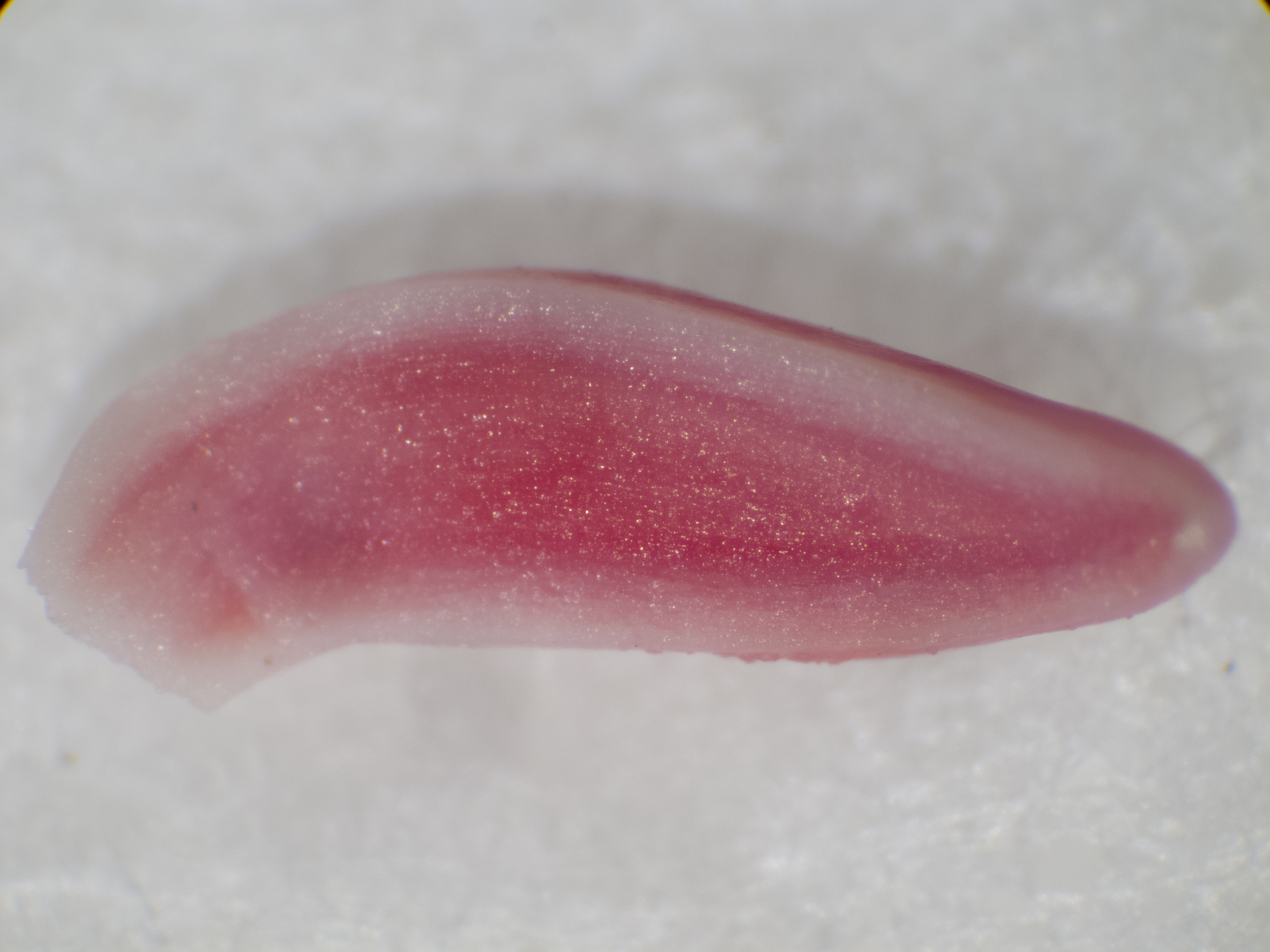

Supplement: S2 Supporting Information — (ZIP) [file pone.0218513.s006.zip › S2_Supporting Information.zip/6+24.jpg]

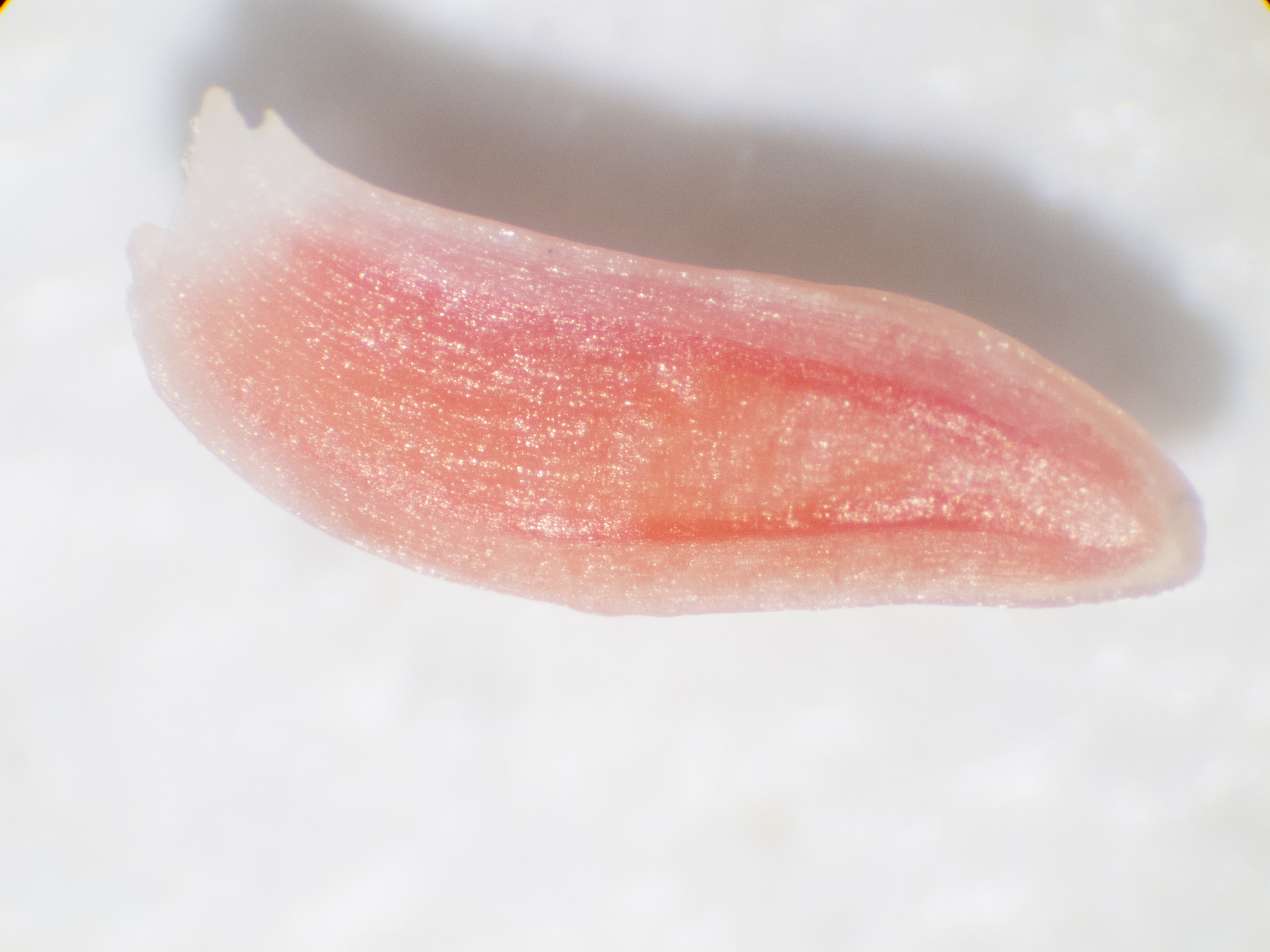

Supplement: S2 Supporting Information — (ZIP) [file pone.0218513.s006.zip › S2_Supporting Information.zip/6.jpg]

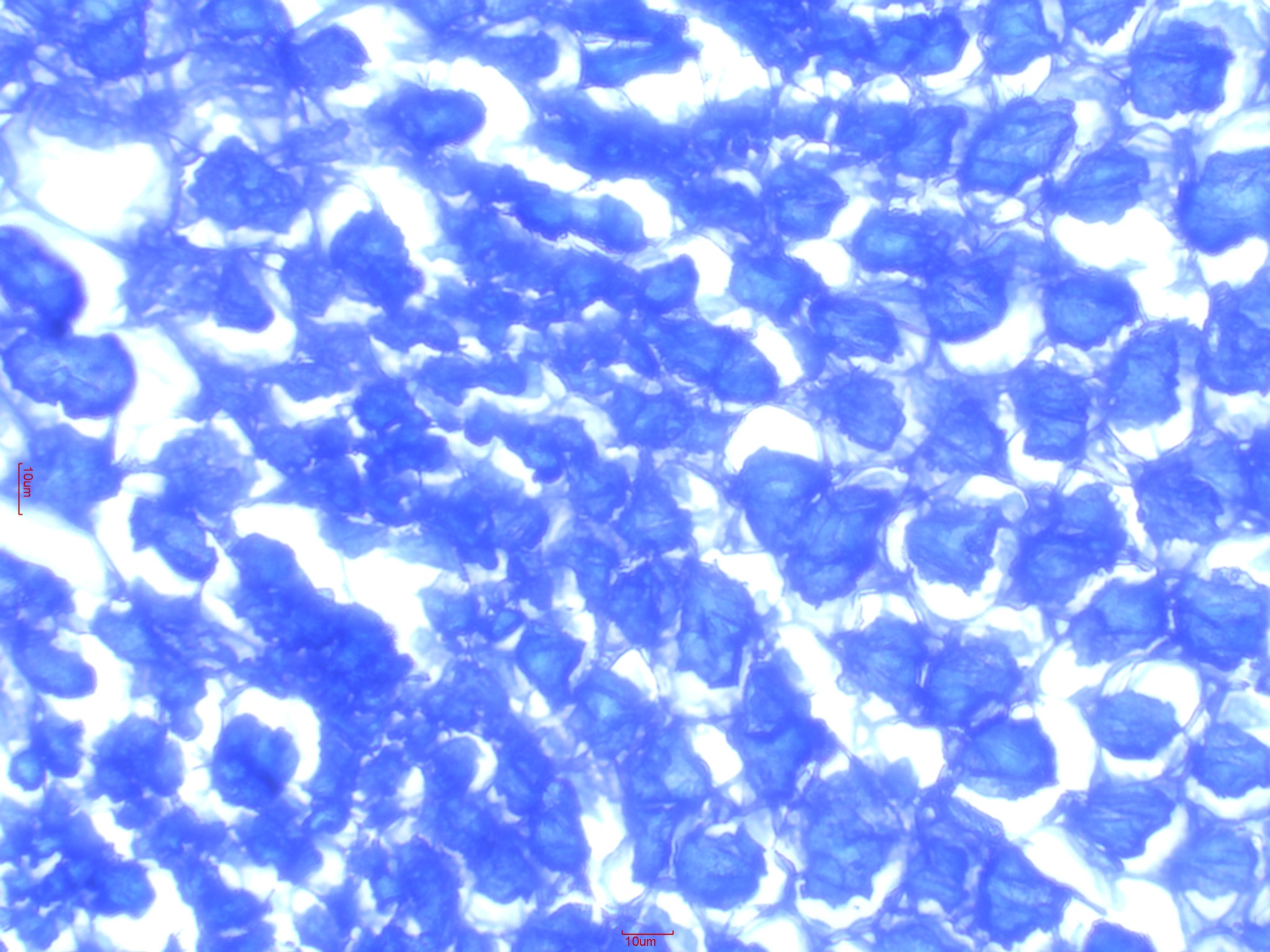

Supplement: S3 Supporting Information — (ZIP) [file pone.0218513.s007.zip › S3_Supporting Information.zip/╣Γ╛╡1.jpg]

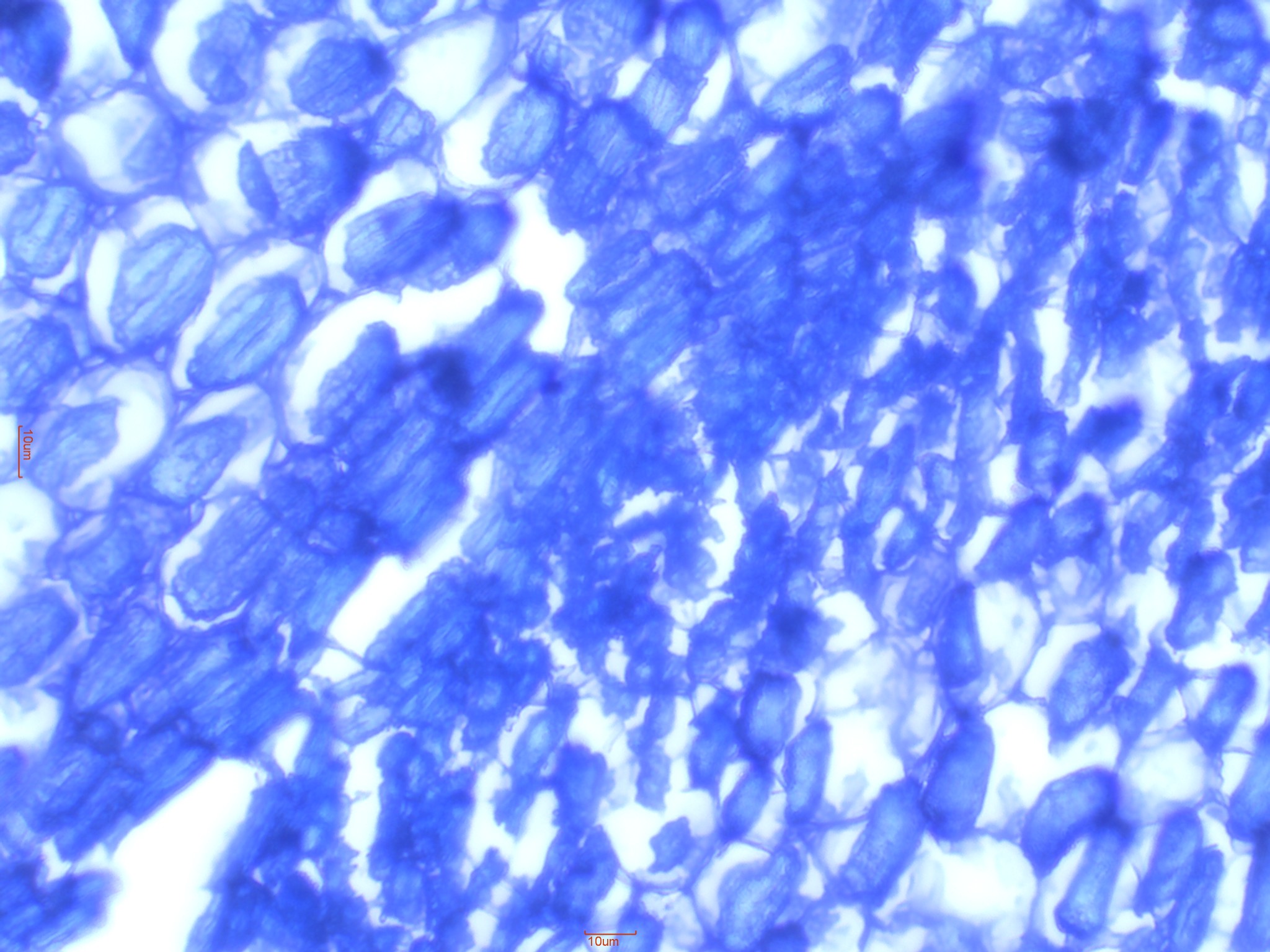

Supplement: S3 Supporting Information — (ZIP) [file pone.0218513.s007.zip › S3_Supporting Information.zip/╣Γ╛╡2.jpg]

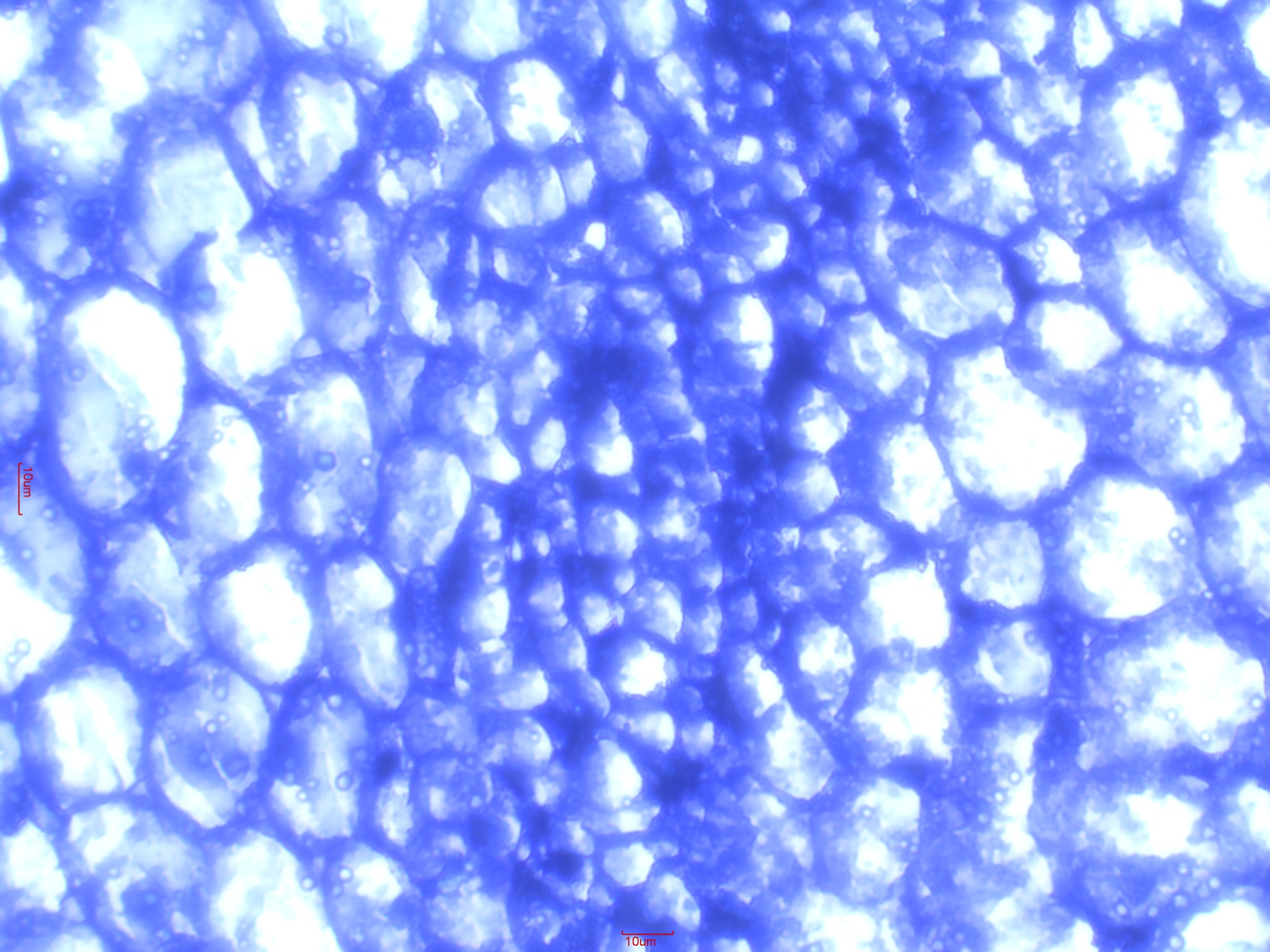

Supplement: S3 Supporting Information — (ZIP) [file pone.0218513.s007.zip › S3_Supporting Information.zip/╣Γ╛╡3.jpg]

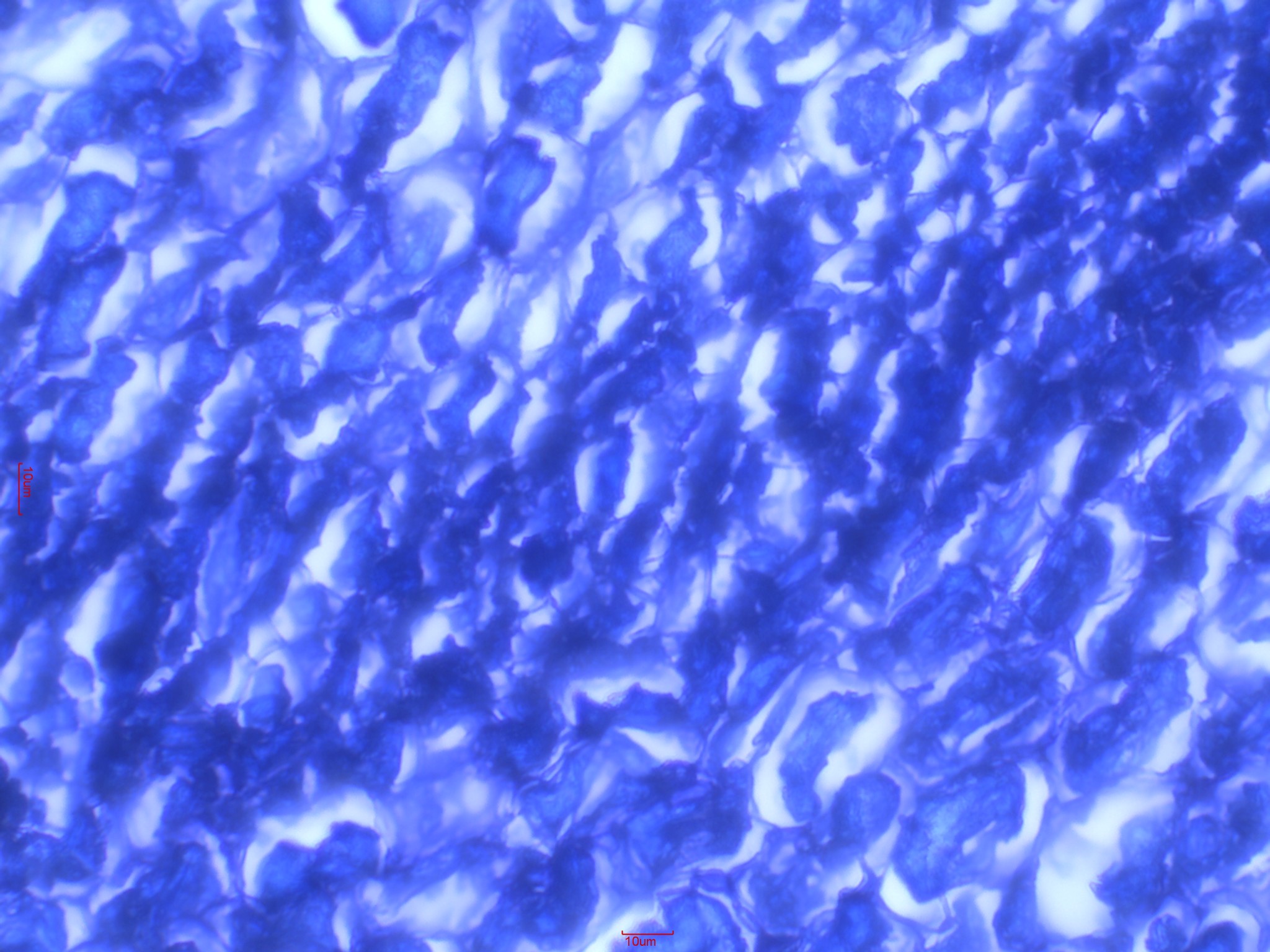

Supplement: S3 Supporting Information — (ZIP) [file pone.0218513.s007.zip › S3_Supporting Information.zip/╣Γ╛╡4.jpg]

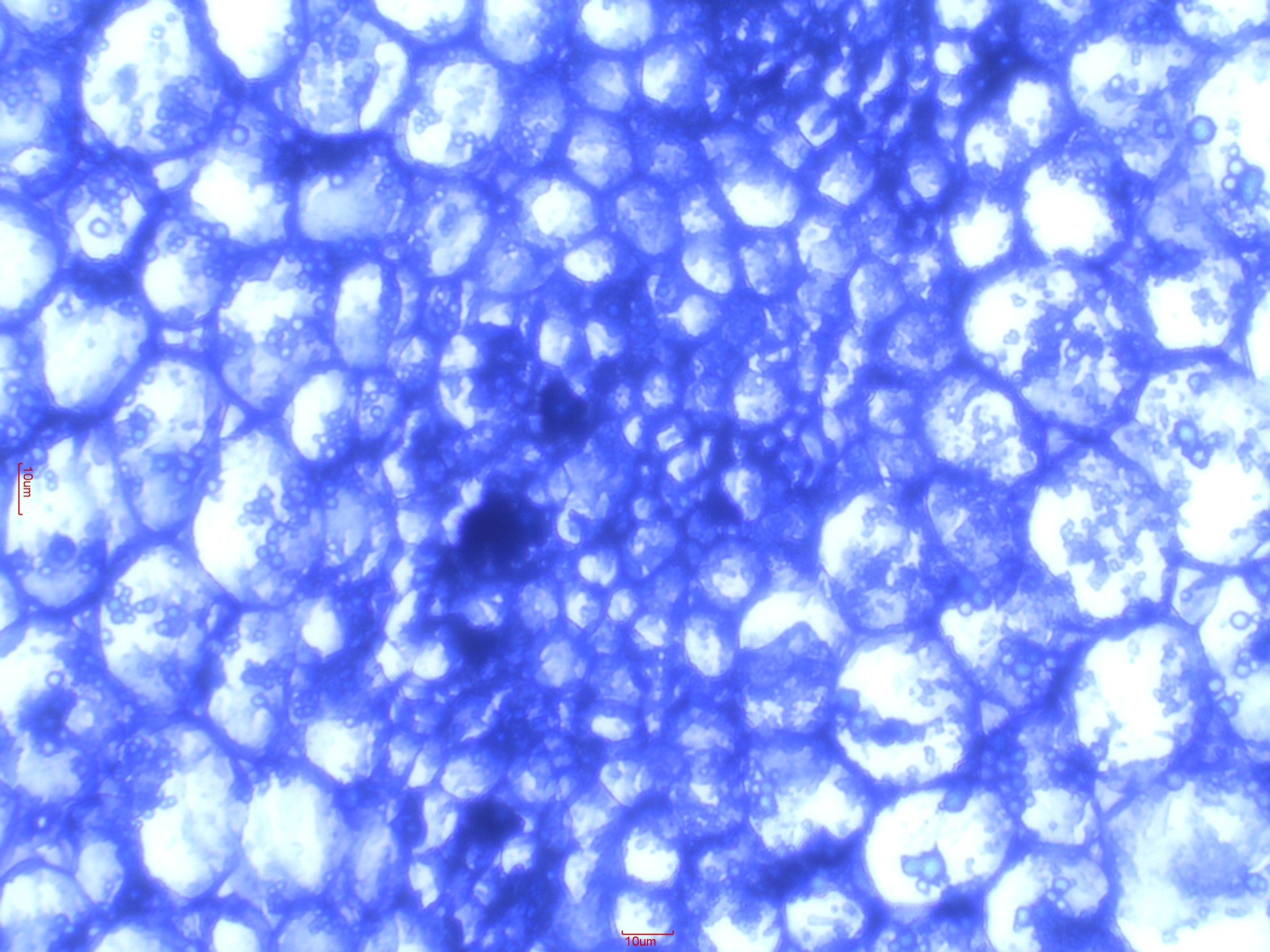

Supplement: S3 Supporting Information — (ZIP) [file pone.0218513.s007.zip › S3_Supporting Information.zip/╣Γ╛╡5.jpg]

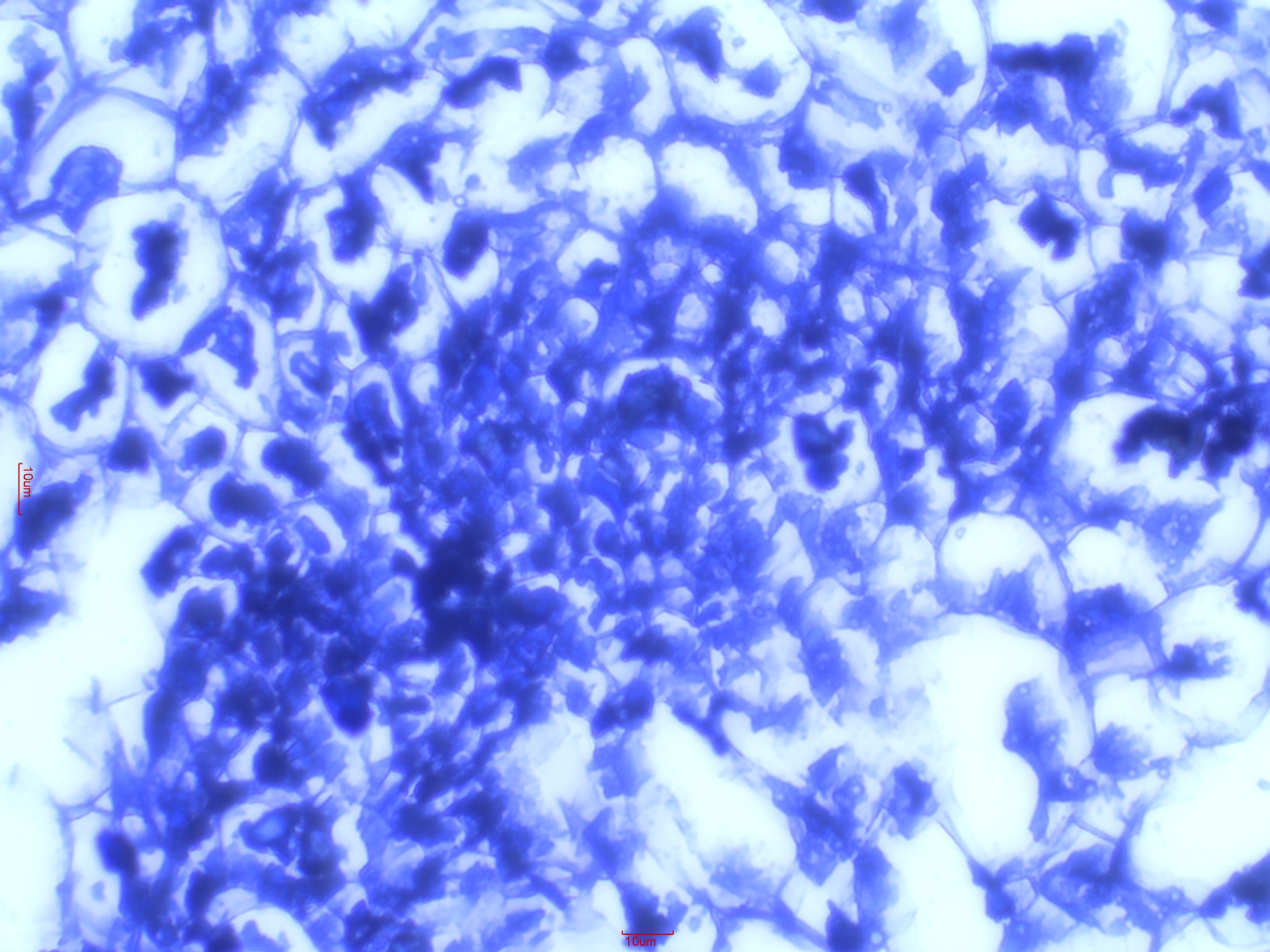

Supplement: S3 Supporting Information — (ZIP) [file pone.0218513.s007.zip › S3_Supporting Information.zip/╣Γ╛╡6.jpg]

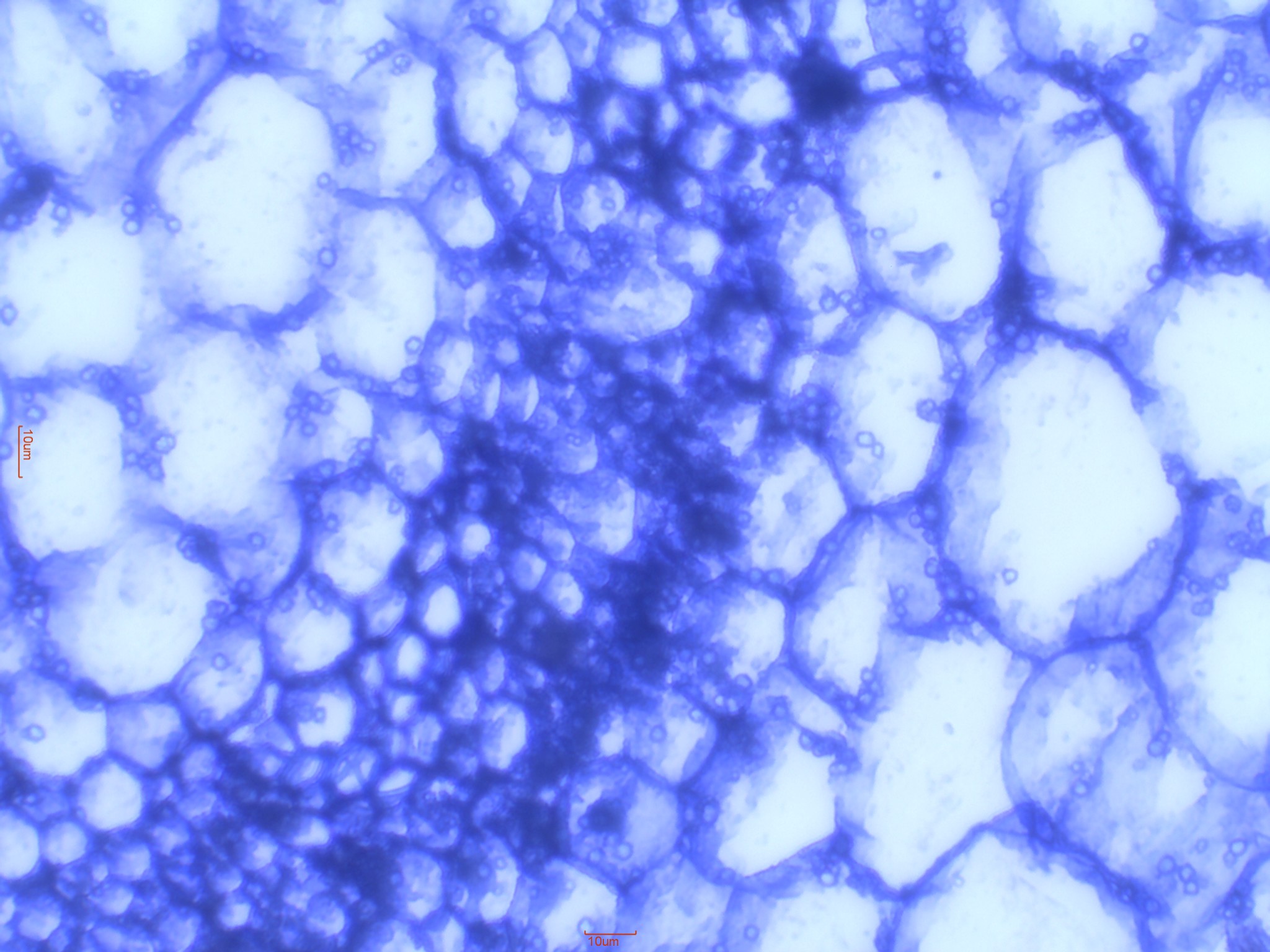

Supplement: S3 Supporting Information — (ZIP) [file pone.0218513.s007.zip › S3_Supporting Information.zip/╣Γ╛╡7.jpg]

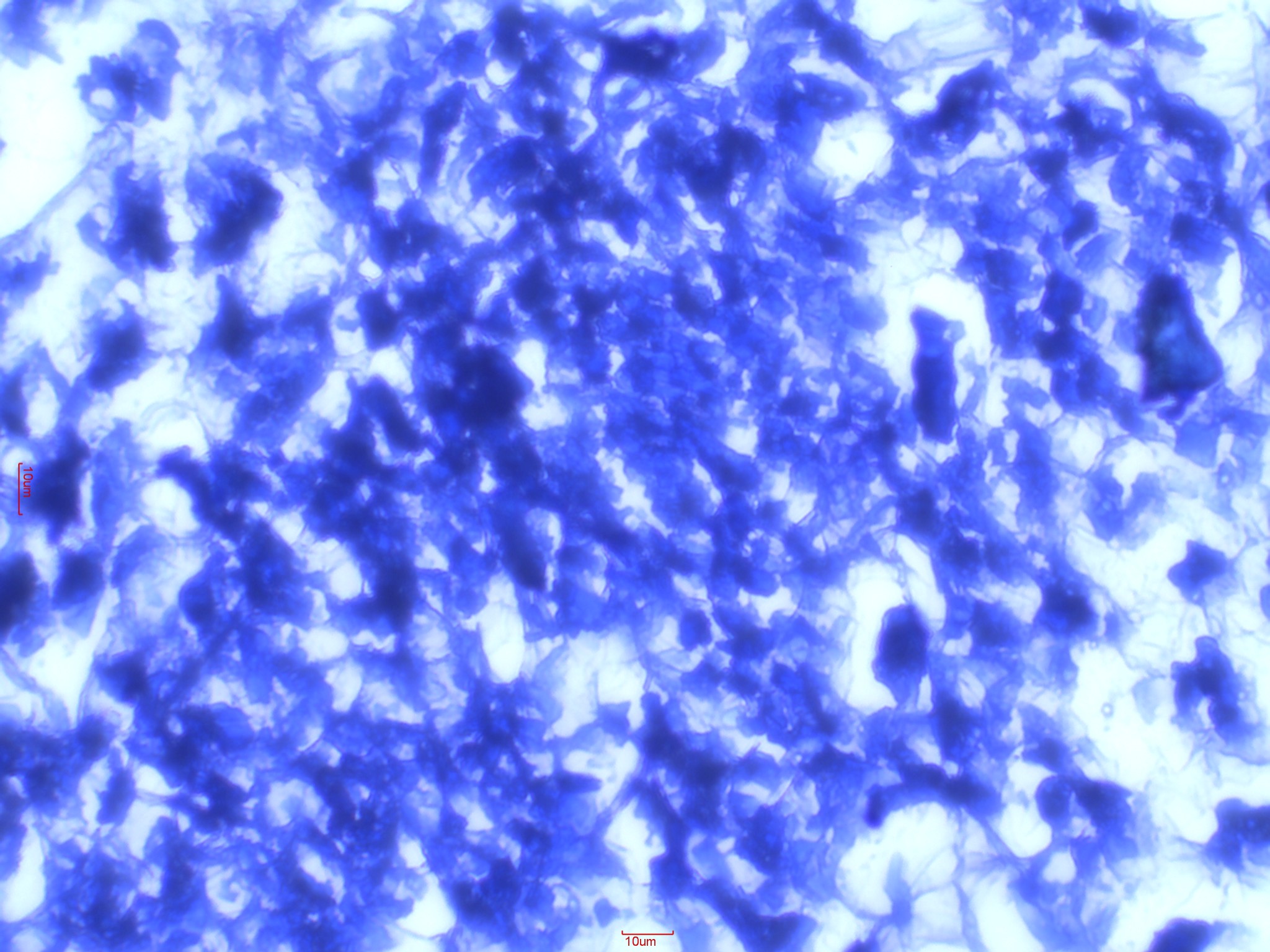

Supplement: S3 Supporting Information — (ZIP) [file pone.0218513.s007.zip › S3_Supporting Information.zip/╣Γ╛╡8.jpg]

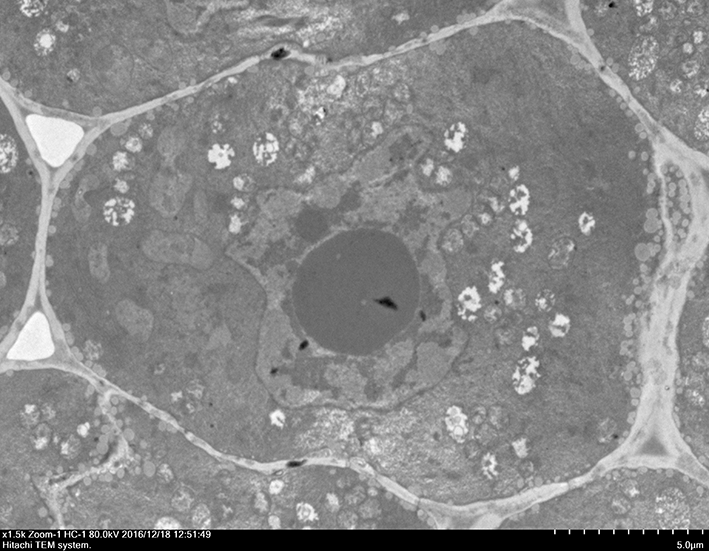

Supplement: S4 Supporting Information — (ZIP) [file pone.0218513.s008.zip › S4_Supporting Information.zip/A.tif]

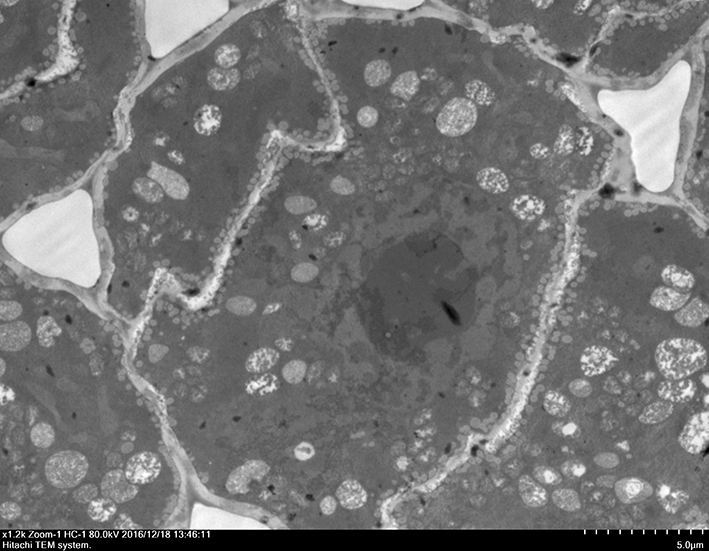

Supplement: S4 Supporting Information — (ZIP) [file pone.0218513.s008.zip › S4_Supporting Information.zip/B.tif]

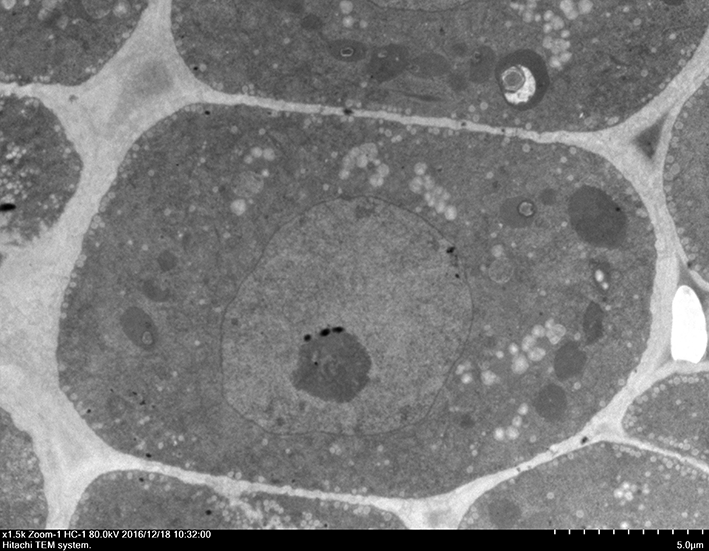

Supplement: S4 Supporting Information — (ZIP) [file pone.0218513.s008.zip › S4_Supporting Information.zip/C.tif]

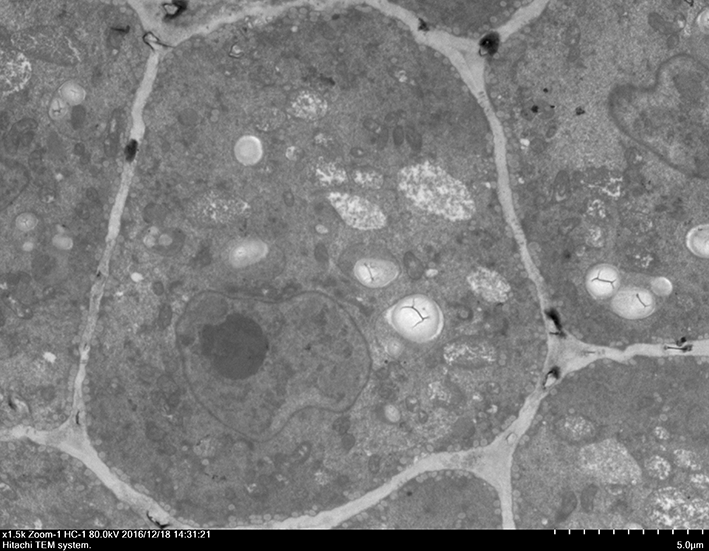

Supplement: S4 Supporting Information — (ZIP) [file pone.0218513.s008.zip › S4_Supporting Information.zip/D.tif]

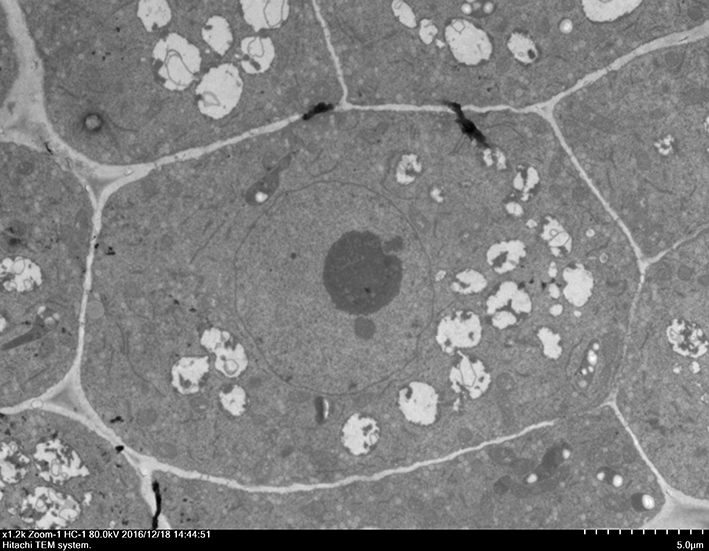

Supplement: S4 Supporting Information — (ZIP) [file pone.0218513.s008.zip › S4_Supporting Information.zip/E.tif]

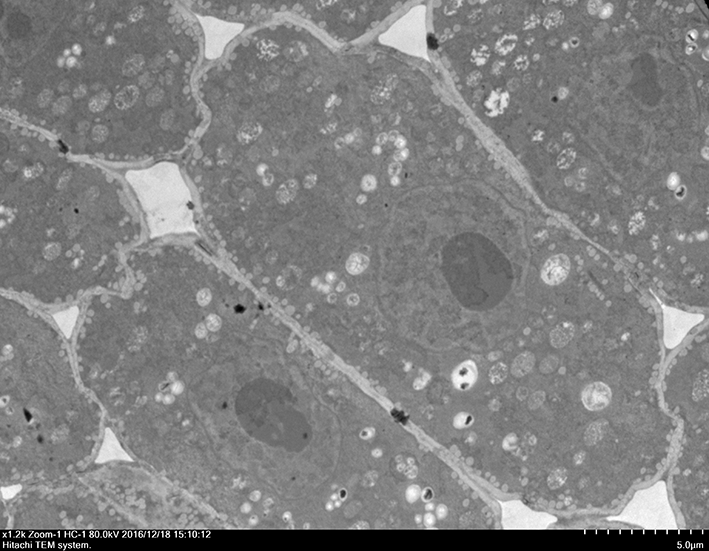

Supplement: S4 Supporting Information — (ZIP) [file pone.0218513.s008.zip › S4_Supporting Information.zip/F.tif]

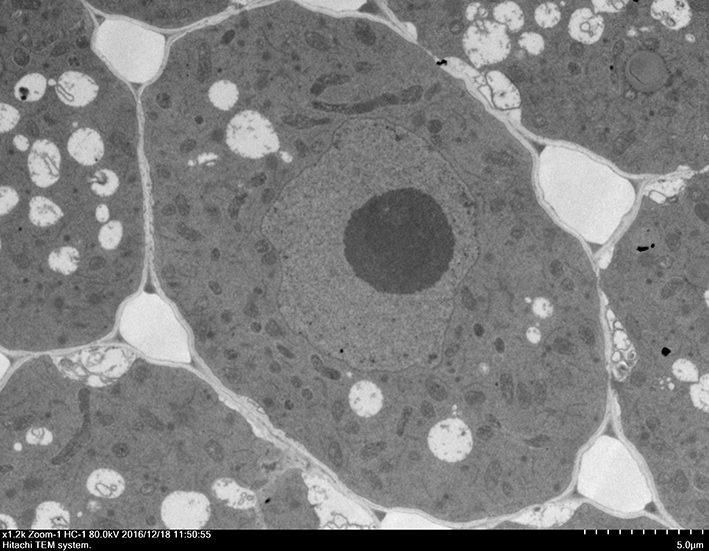

Supplement: S4 Supporting Information — (ZIP) [file pone.0218513.s008.zip › S4_Supporting Information.zip/G.tif]

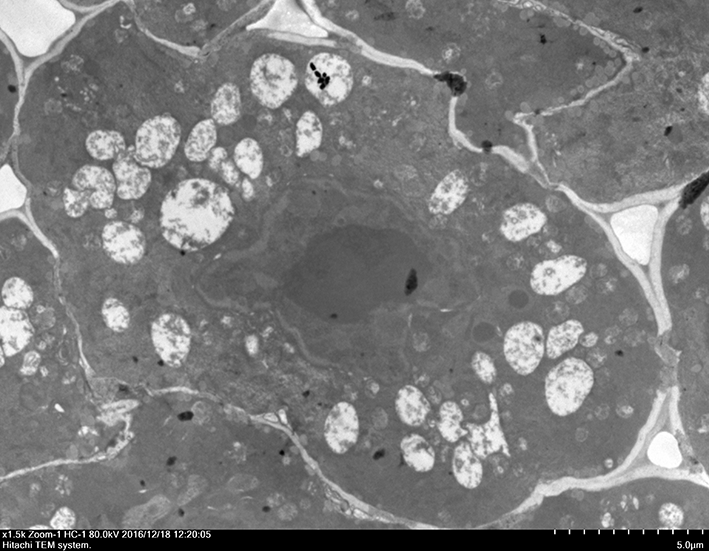

Supplement: S4 Supporting Information — (ZIP) [file pone.0218513.s008.zip › S4_Supporting Information.zip/H.tif]

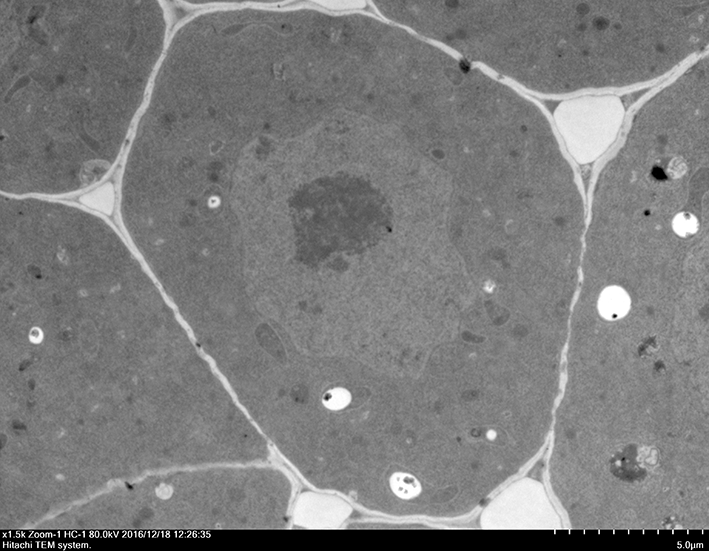

Supplement: S4 Supporting Information — (ZIP) [file pone.0218513.s008.zip › S4_Supporting Information.zip/I.tif]

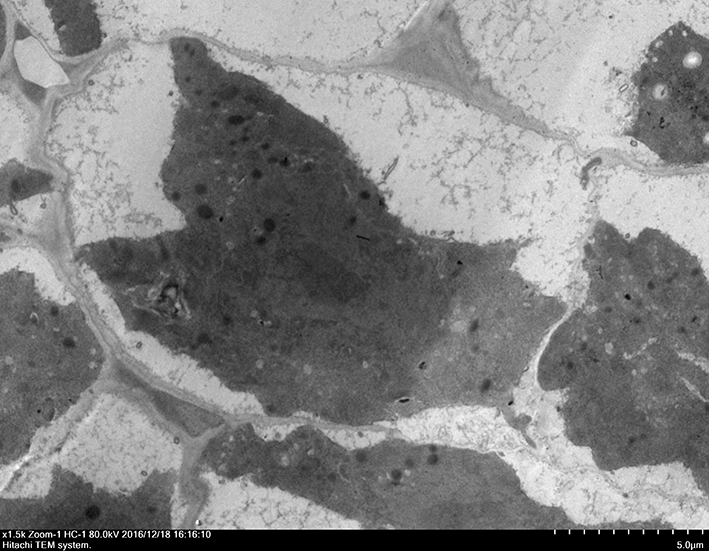

Supplement: S4 Supporting Information — (ZIP) [file pone.0218513.s008.zip › S4_Supporting Information.zip/J.tif]

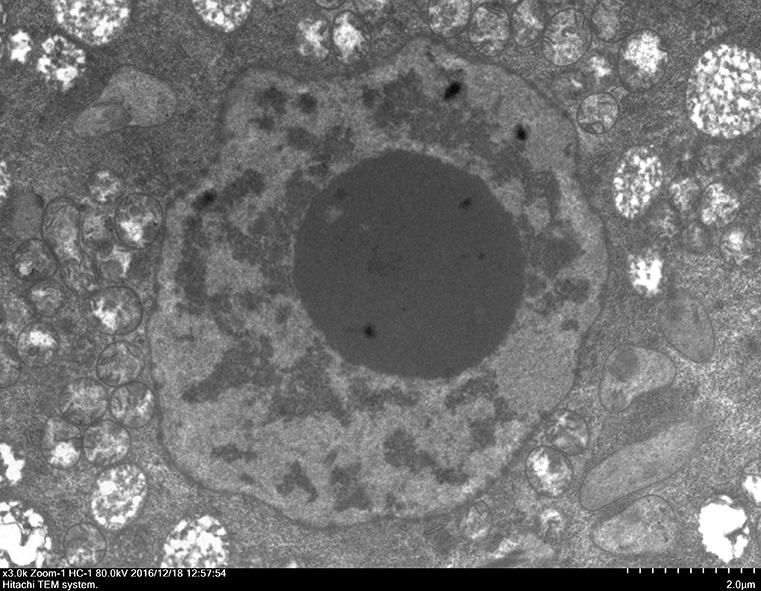

Supplement: S5 Supporting Information — (ZIP) [file pone.0218513.s009.zip › S5_Supporting Information.zip/A.tif]

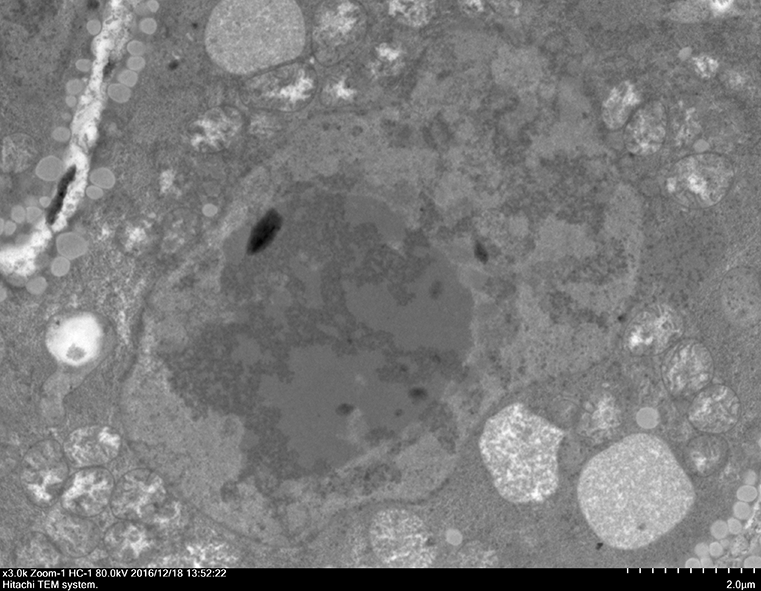

Supplement: S5 Supporting Information — (ZIP) [file pone.0218513.s009.zip › S5_Supporting Information.zip/B.tif]

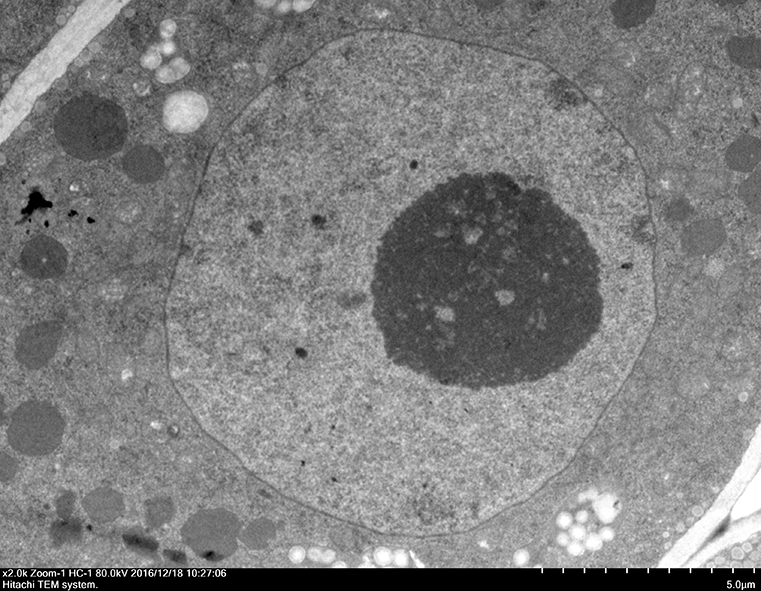

Supplement: S5 Supporting Information — (ZIP) [file pone.0218513.s009.zip › S5_Supporting Information.zip/C.tif]

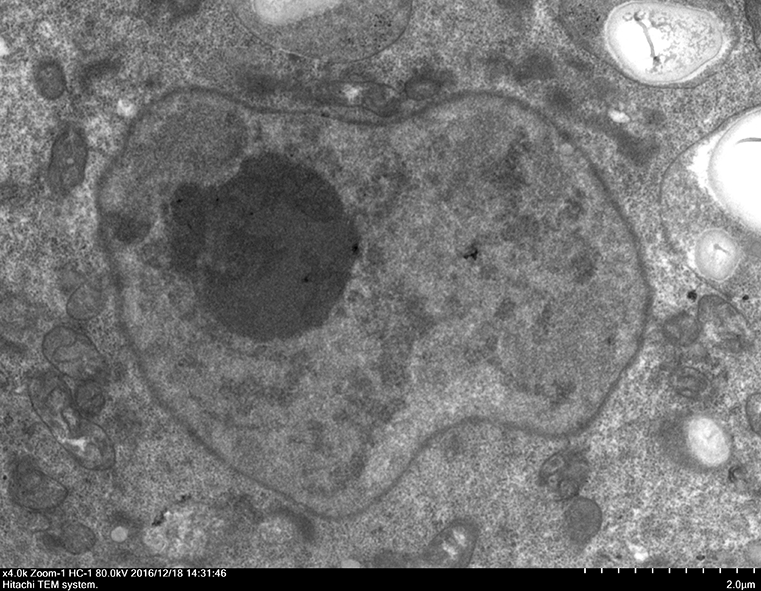

Supplement: S5 Supporting Information — (ZIP) [file pone.0218513.s009.zip › S5_Supporting Information.zip/D.tif]

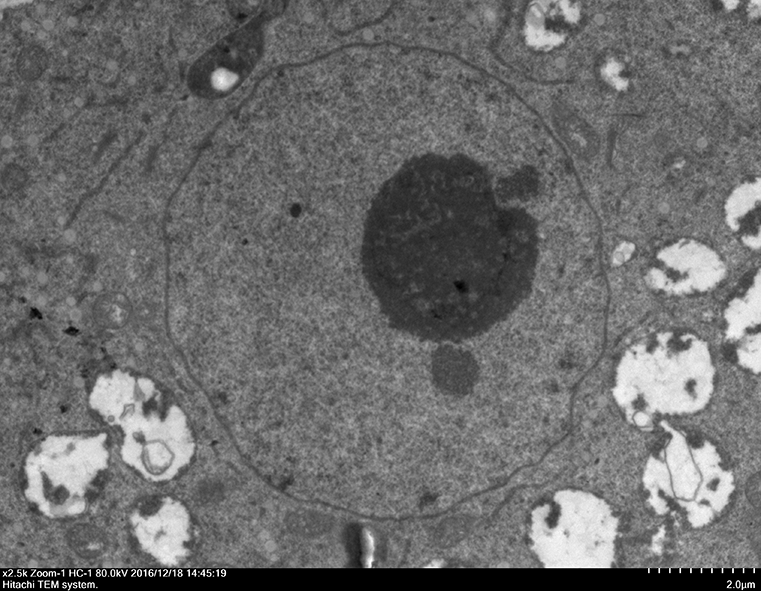

Supplement: S5 Supporting Information — (ZIP) [file pone.0218513.s009.zip › S5_Supporting Information.zip/E.tif]

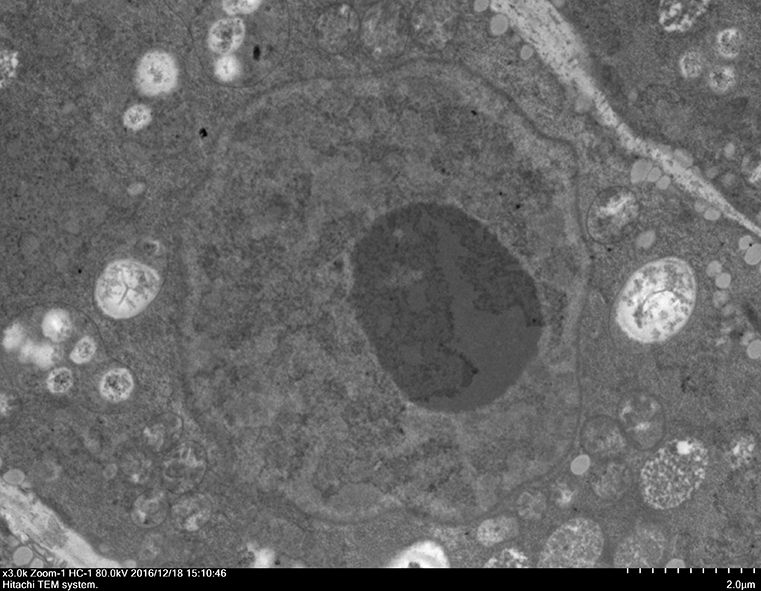

Supplement: S5 Supporting Information — (ZIP) [file pone.0218513.s009.zip › S5_Supporting Information.zip/F.tif]

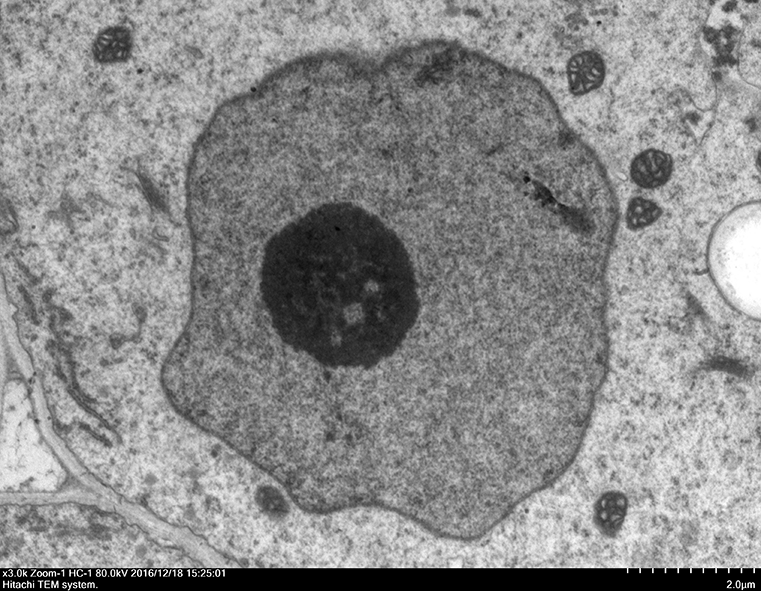

Supplement: S5 Supporting Information — (ZIP) [file pone.0218513.s009.zip › S5_Supporting Information.zip/G.tif]

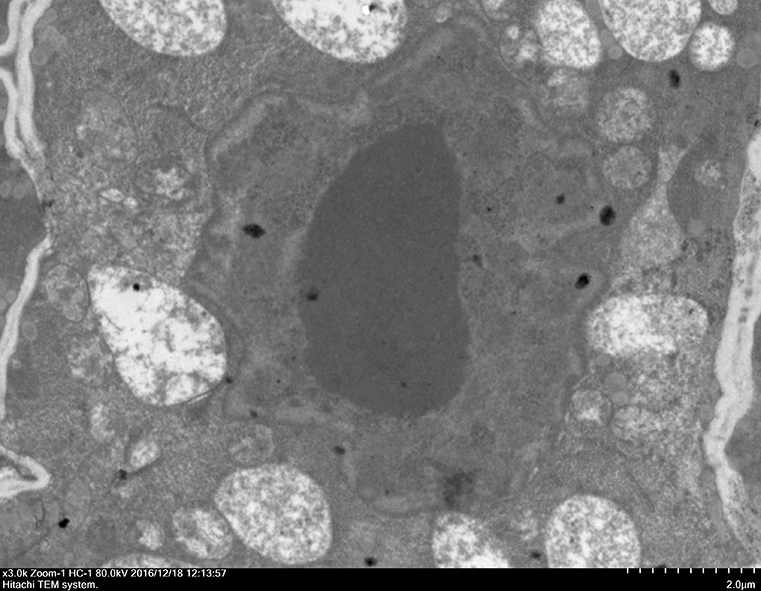

Supplement: S5 Supporting Information — (ZIP) [file pone.0218513.s009.zip › S5_Supporting Information.zip/H.tif]

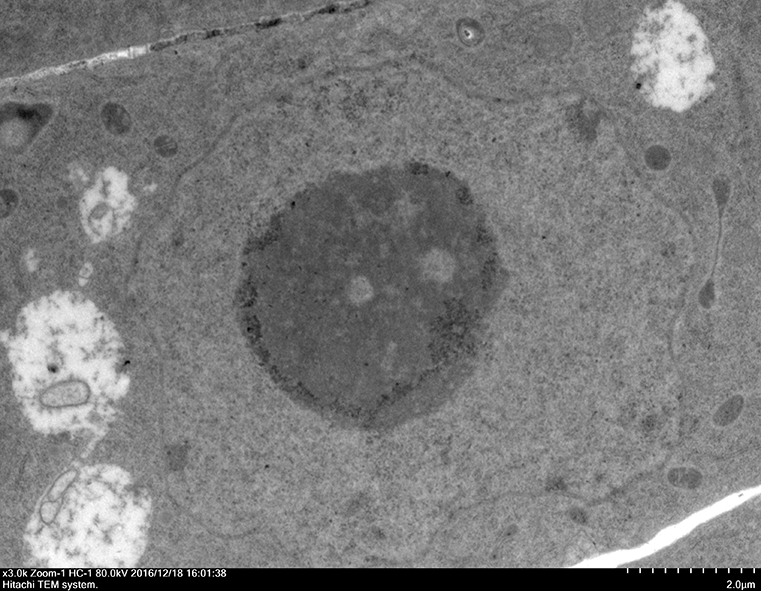

Supplement: S5 Supporting Information — (ZIP) [file pone.0218513.s009.zip › S5_Supporting Information.zip/I.tif]

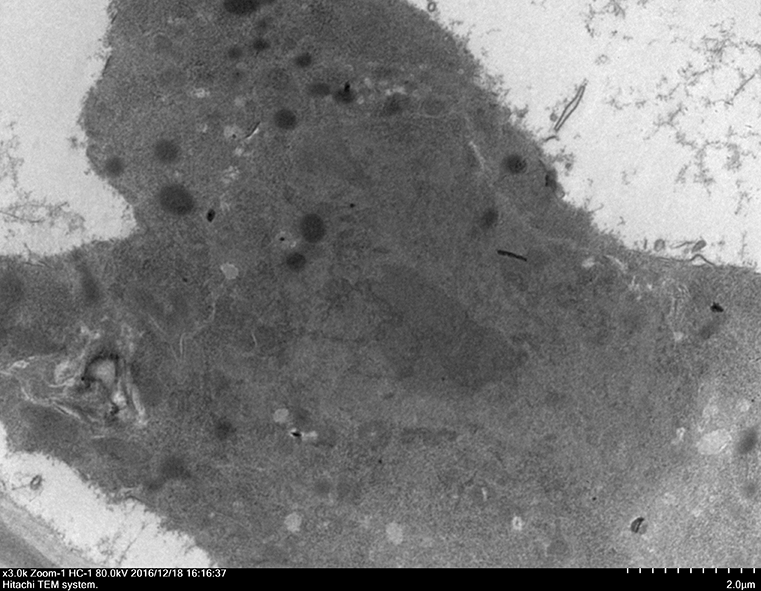

Supplement: S5 Supporting Information — (ZIP) [file pone.0218513.s009.zip › S5_Supporting Information.zip/J.tif]

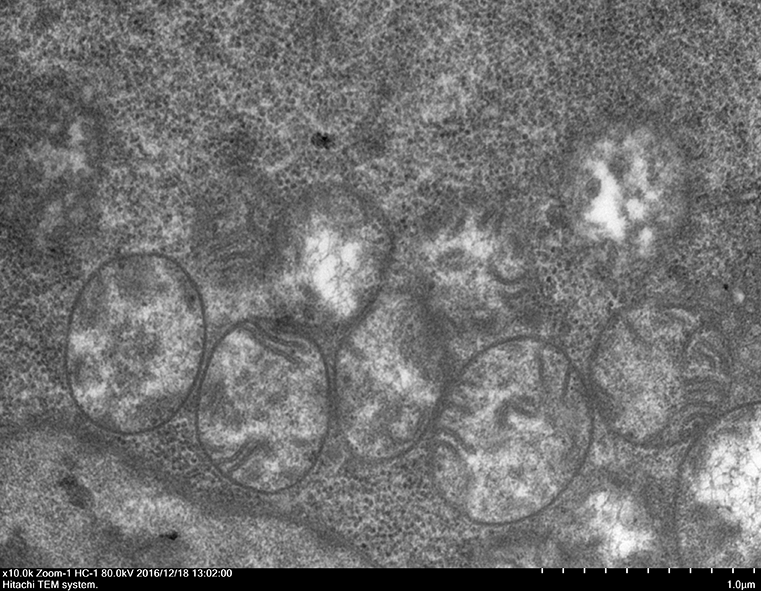

Supplement: S6 Supporting Information — (ZIP) [file pone.0218513.s010.zip › S6_Supporting Information.zip/A.tif]

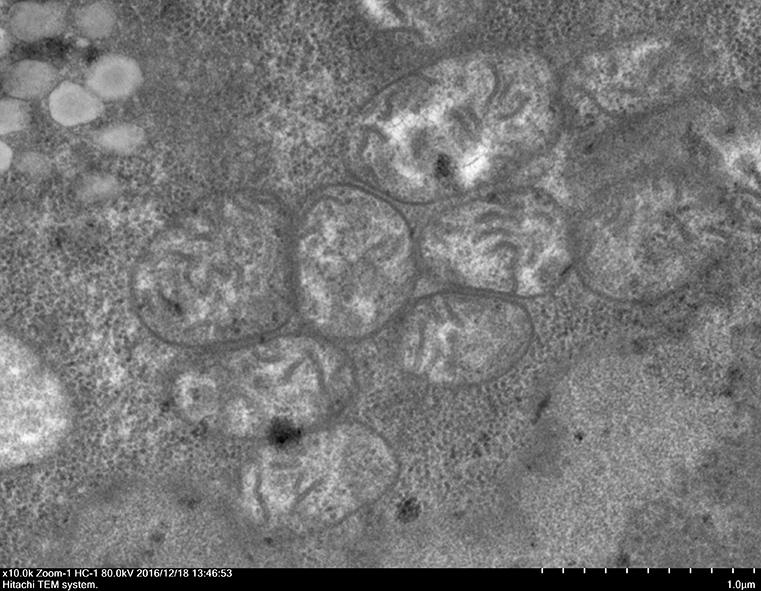

Supplement: S6 Supporting Information — (ZIP) [file pone.0218513.s010.zip › S6_Supporting Information.zip/B.tif]

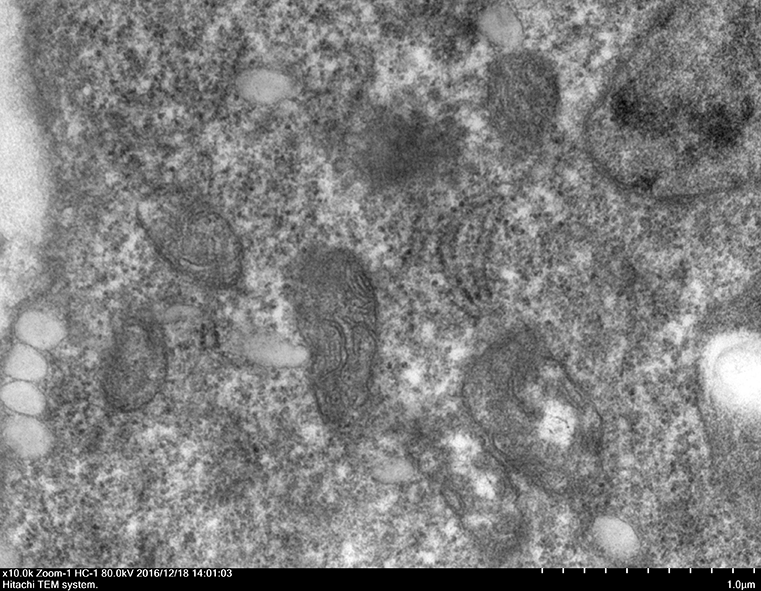

Supplement: S6 Supporting Information — (ZIP) [file pone.0218513.s010.zip › S6_Supporting Information.zip/C.tif]

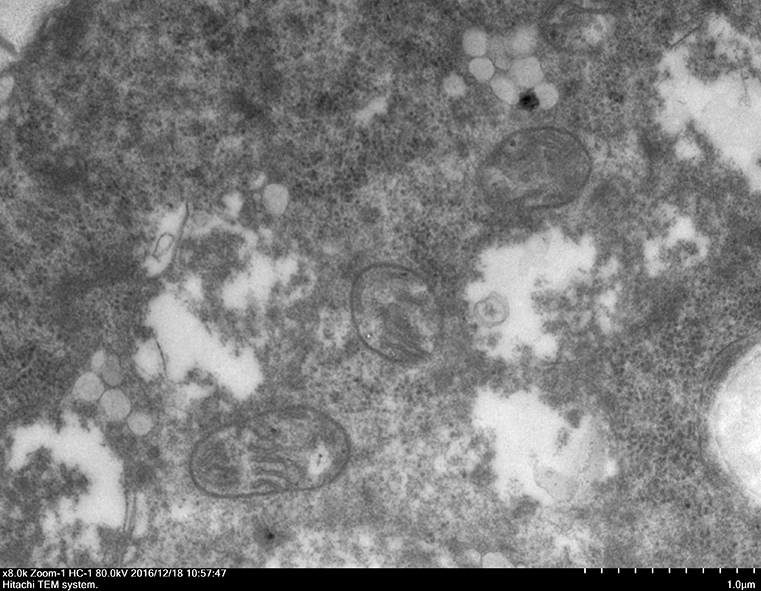

Supplement: S6 Supporting Information — (ZIP) [file pone.0218513.s010.zip › S6_Supporting Information.zip/D.tif]

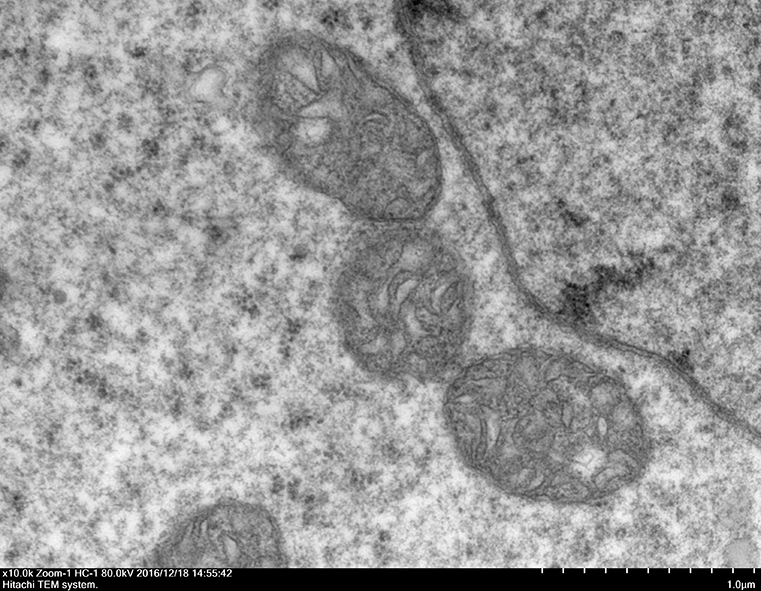

Supplement: S6 Supporting Information — (ZIP) [file pone.0218513.s010.zip › S6_Supporting Information.zip/E.tif]

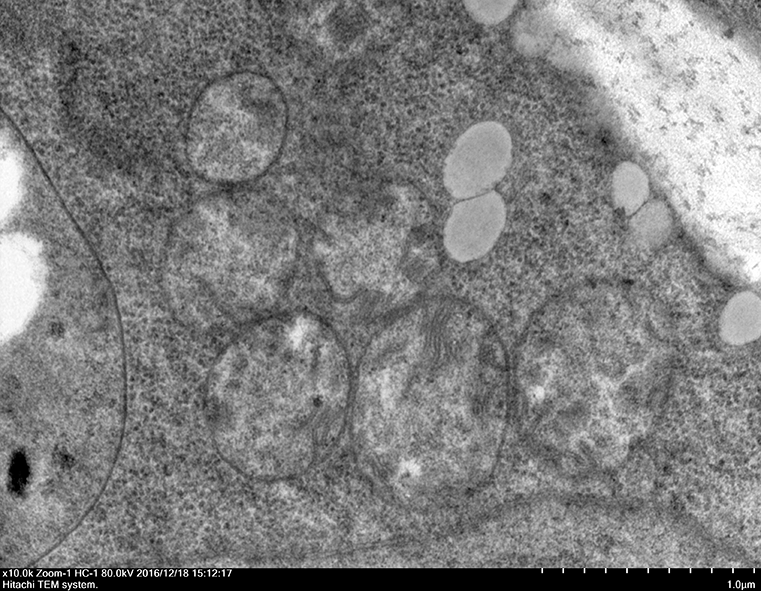

Supplement: S6 Supporting Information — (ZIP) [file pone.0218513.s010.zip › S6_Supporting Information.zip/F.tif]

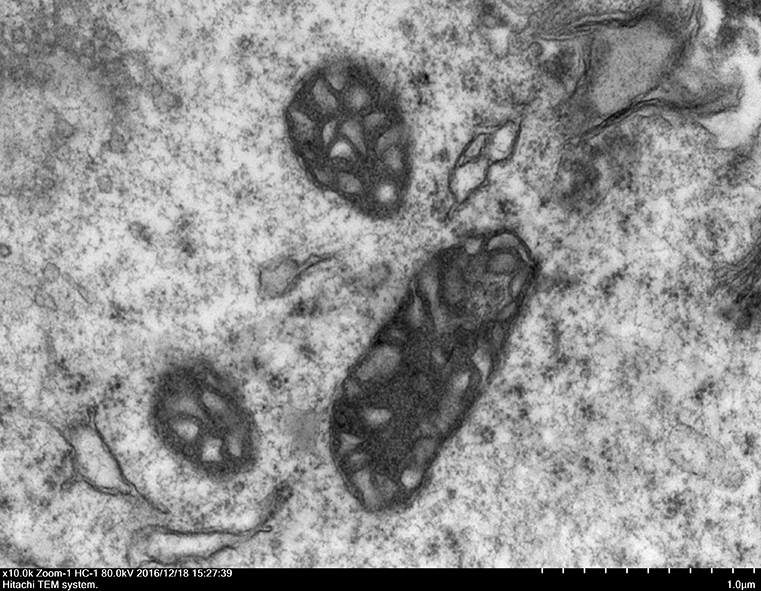

Supplement: S6 Supporting Information — (ZIP) [file pone.0218513.s010.zip › S6_Supporting Information.zip/G.tif]

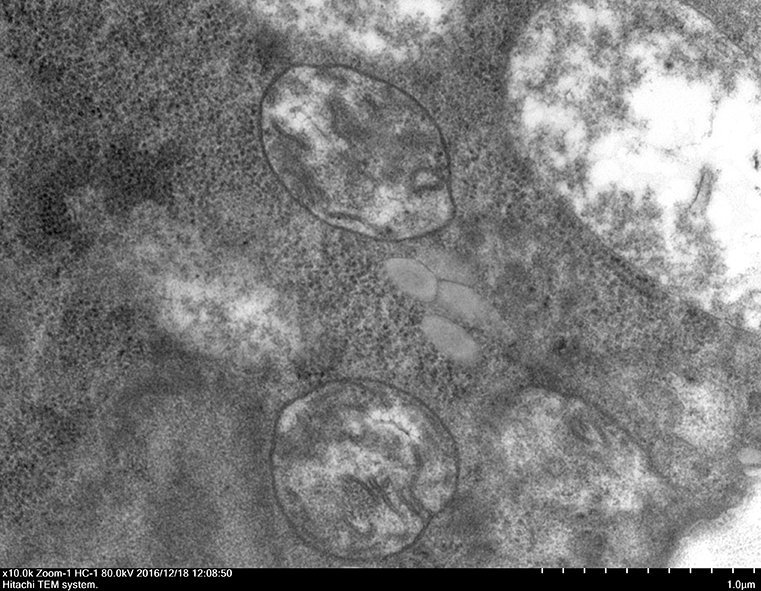

Supplement: S6 Supporting Information — (ZIP) [file pone.0218513.s010.zip › S6_Supporting Information.zip/H.tif]

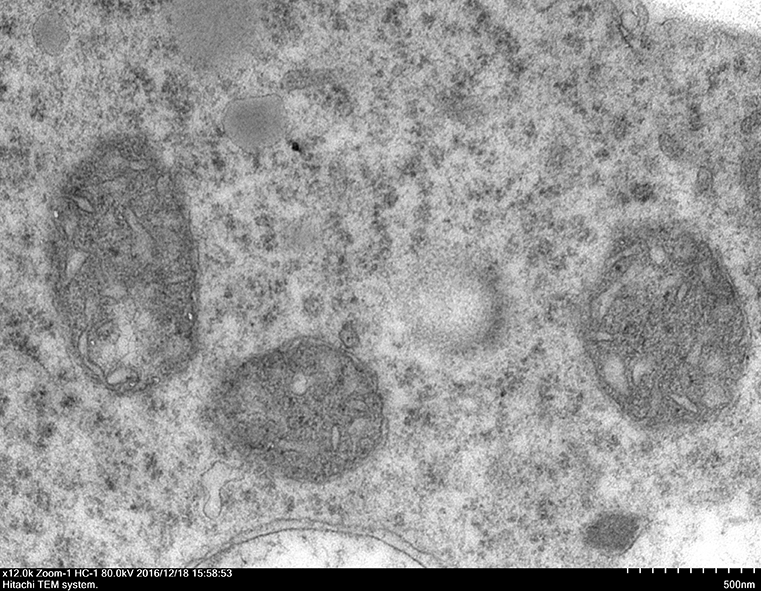

Supplement: S6 Supporting Information — (ZIP) [file pone.0218513.s010.zip › S6_Supporting Information.zip/I.tif]

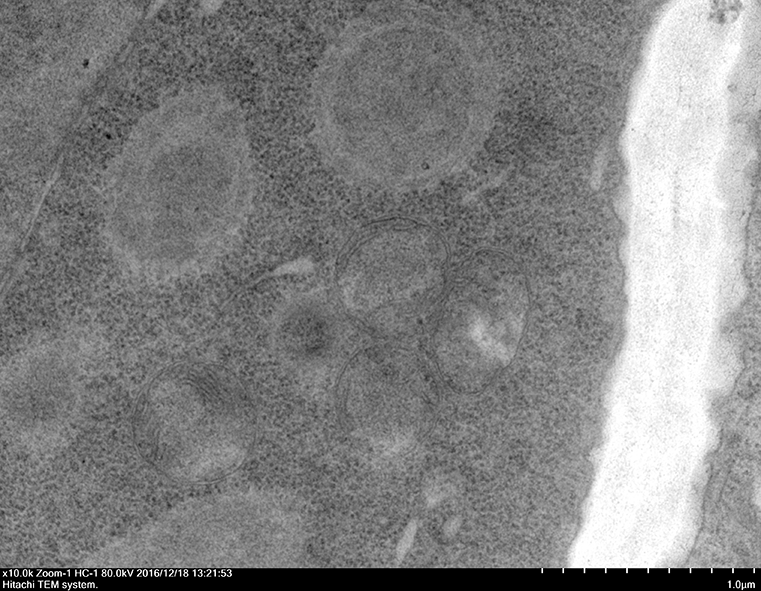

Supplement: S6 Supporting Information — (ZIP) [file pone.0218513.s010.zip › S6_Supporting Information.zip/J.tif]

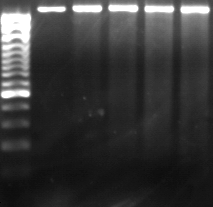

Supplement: S7 Supporting Information — (ZIP) [file pone.0218513.s011.zip › S7_Supporting Information.zip/desiccation.tif]

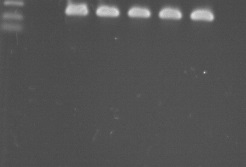

Supplement: S7 Supporting Information — (ZIP) [file pone.0218513.s011.zip › S7_Supporting Information.zip/undesiccation.jpg]

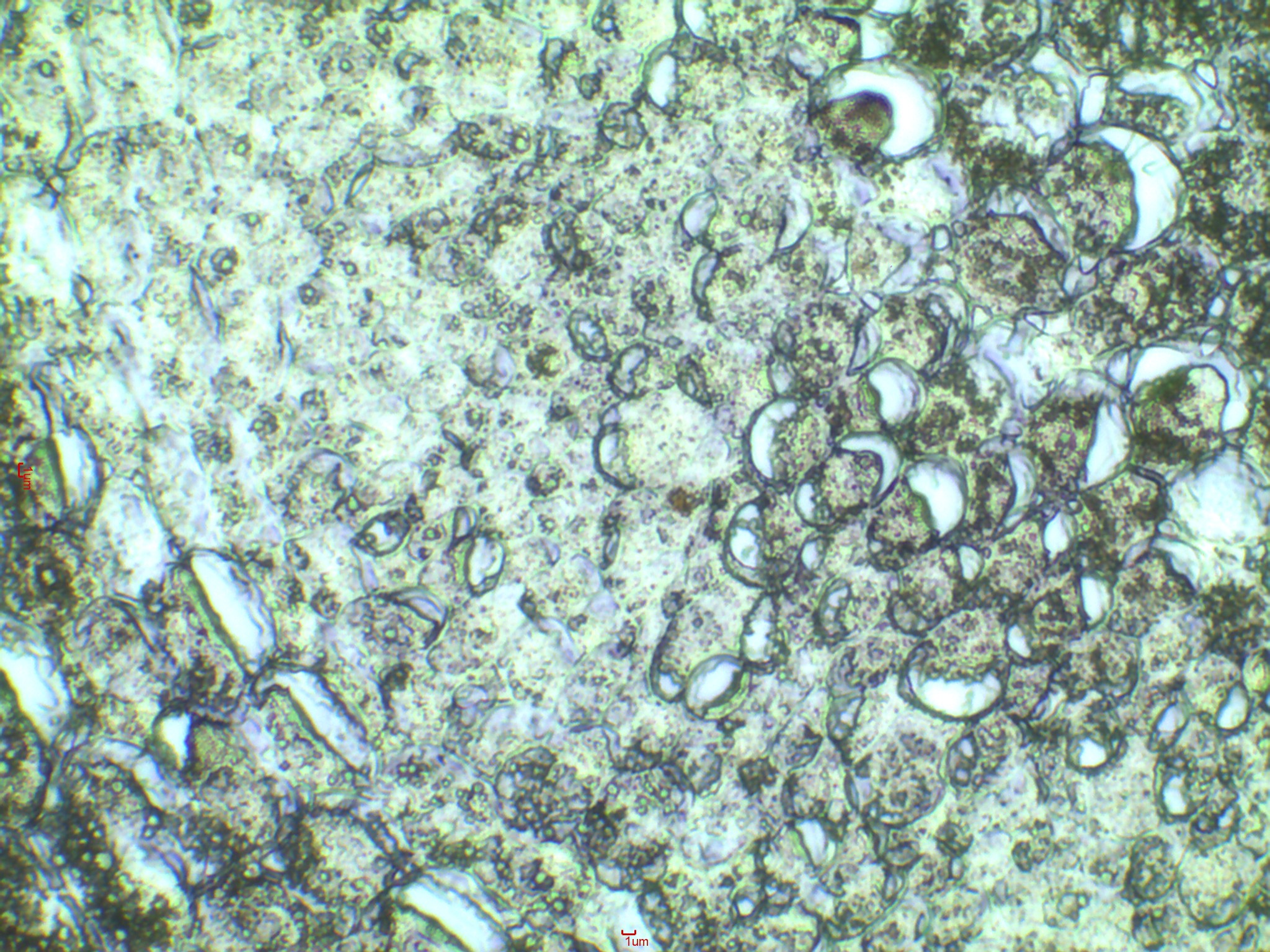

Supplement: S8 Supporting Information — (ZIP) [file pone.0218513.s012.zip › S8_Supporting Information.zip/A.jpg]

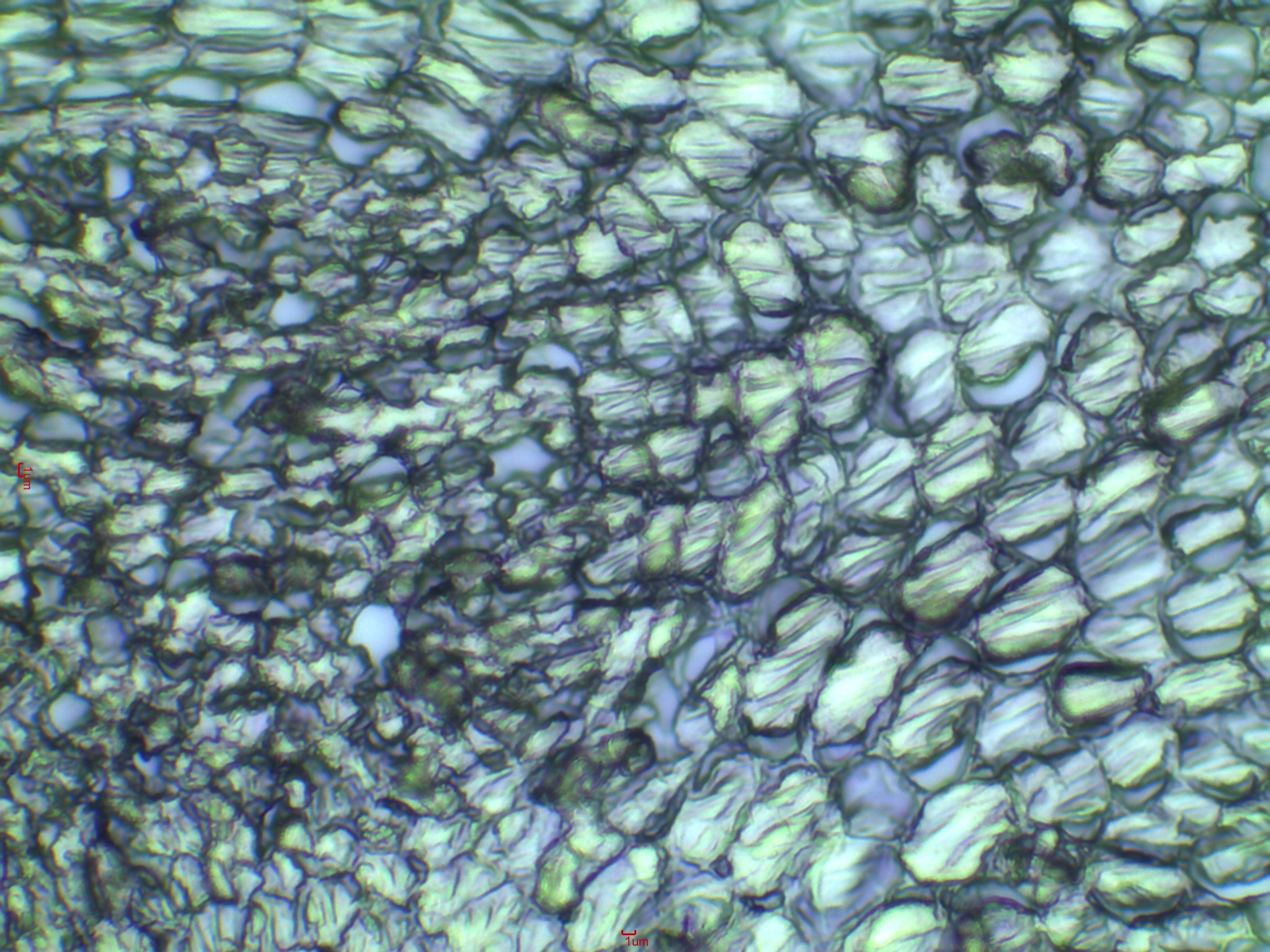

Supplement: S8 Supporting Information — (ZIP) [file pone.0218513.s012.zip › S8_Supporting Information.zip/B.jpg]

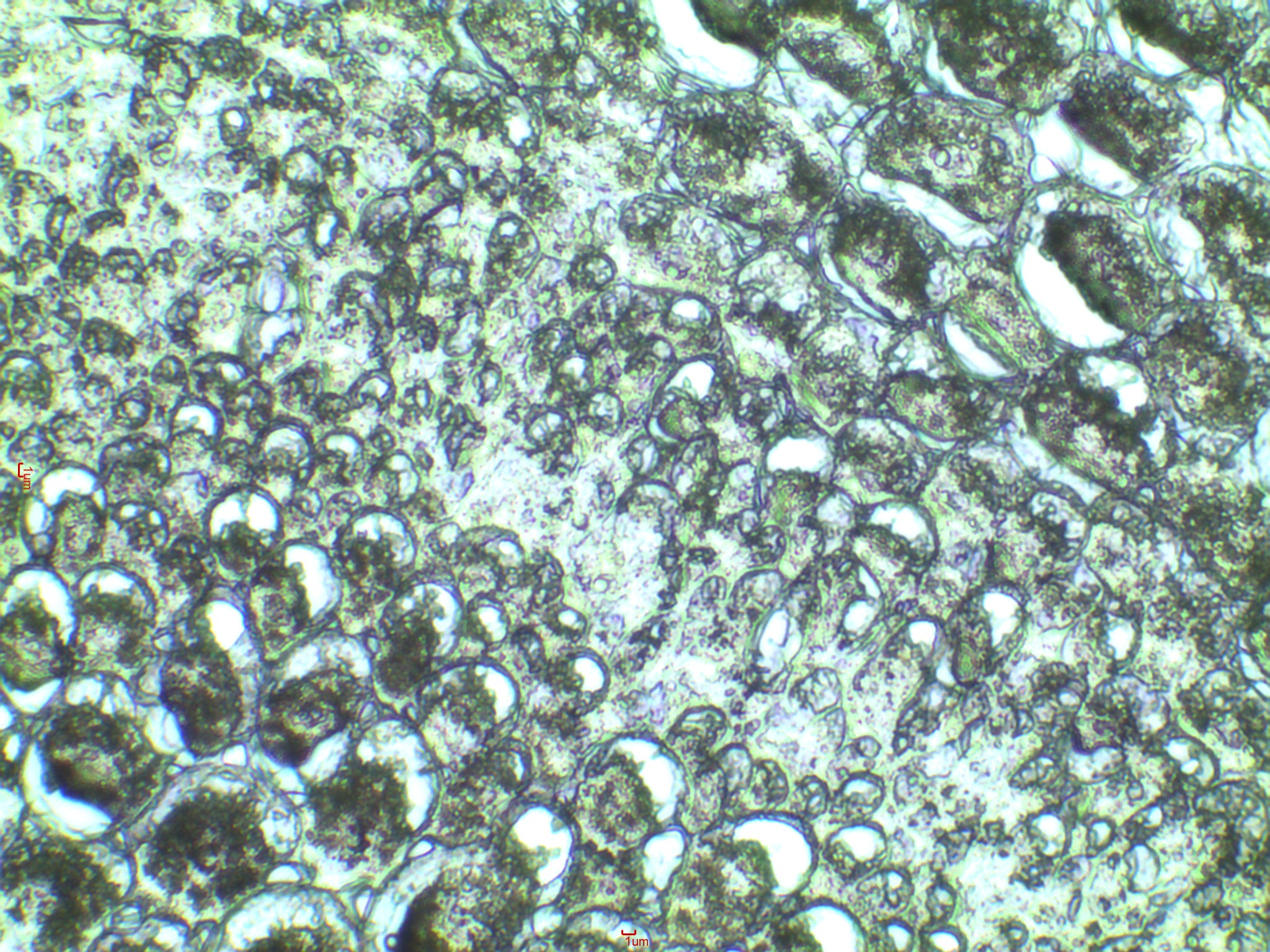

Supplement: S8 Supporting Information — (ZIP) [file pone.0218513.s012.zip › S8_Supporting Information.zip/C.jpg]

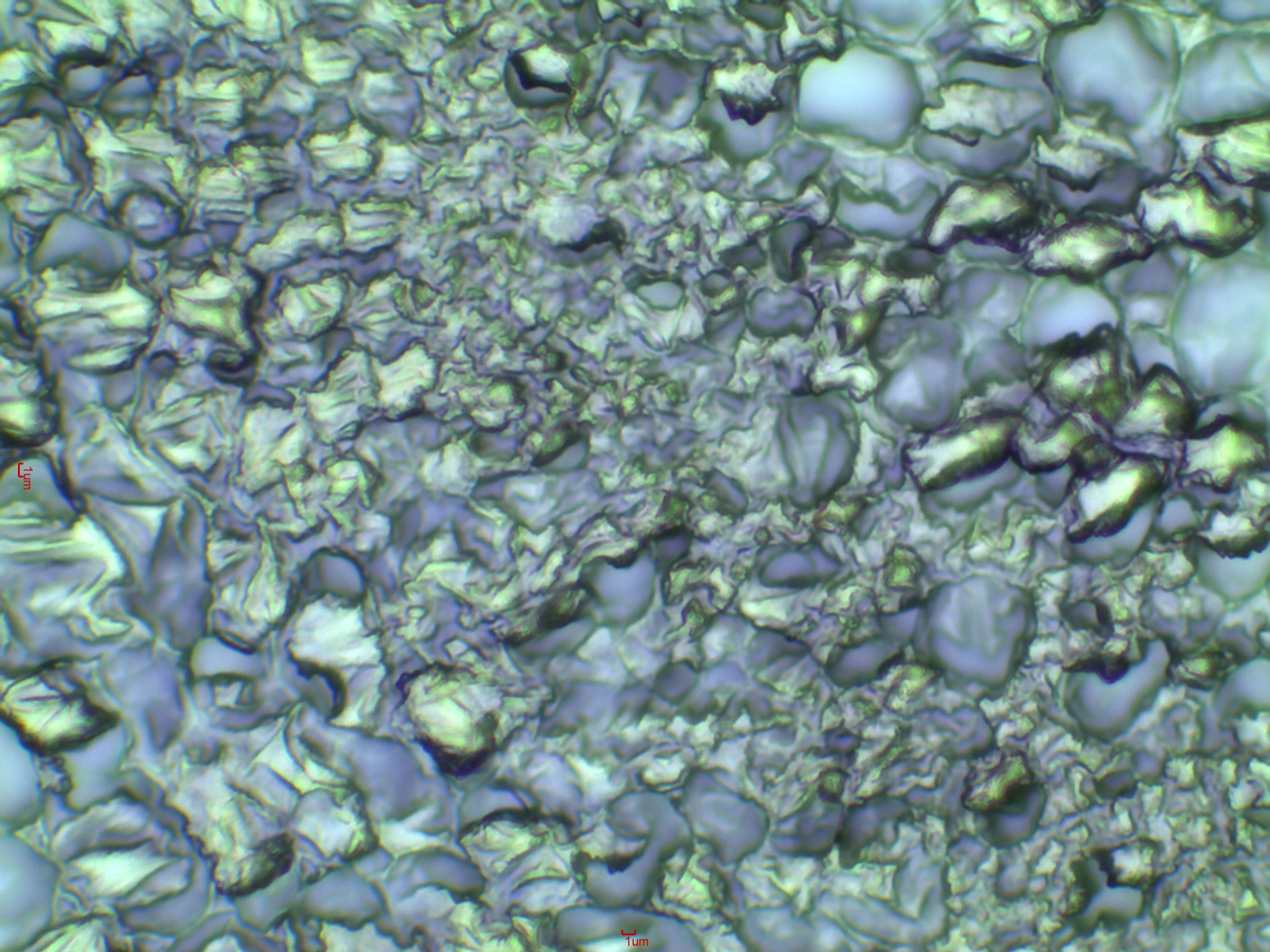

Supplement: S8 Supporting Information — (ZIP) [file pone.0218513.s012.zip › S8_Supporting Information.zip/D.jpg]

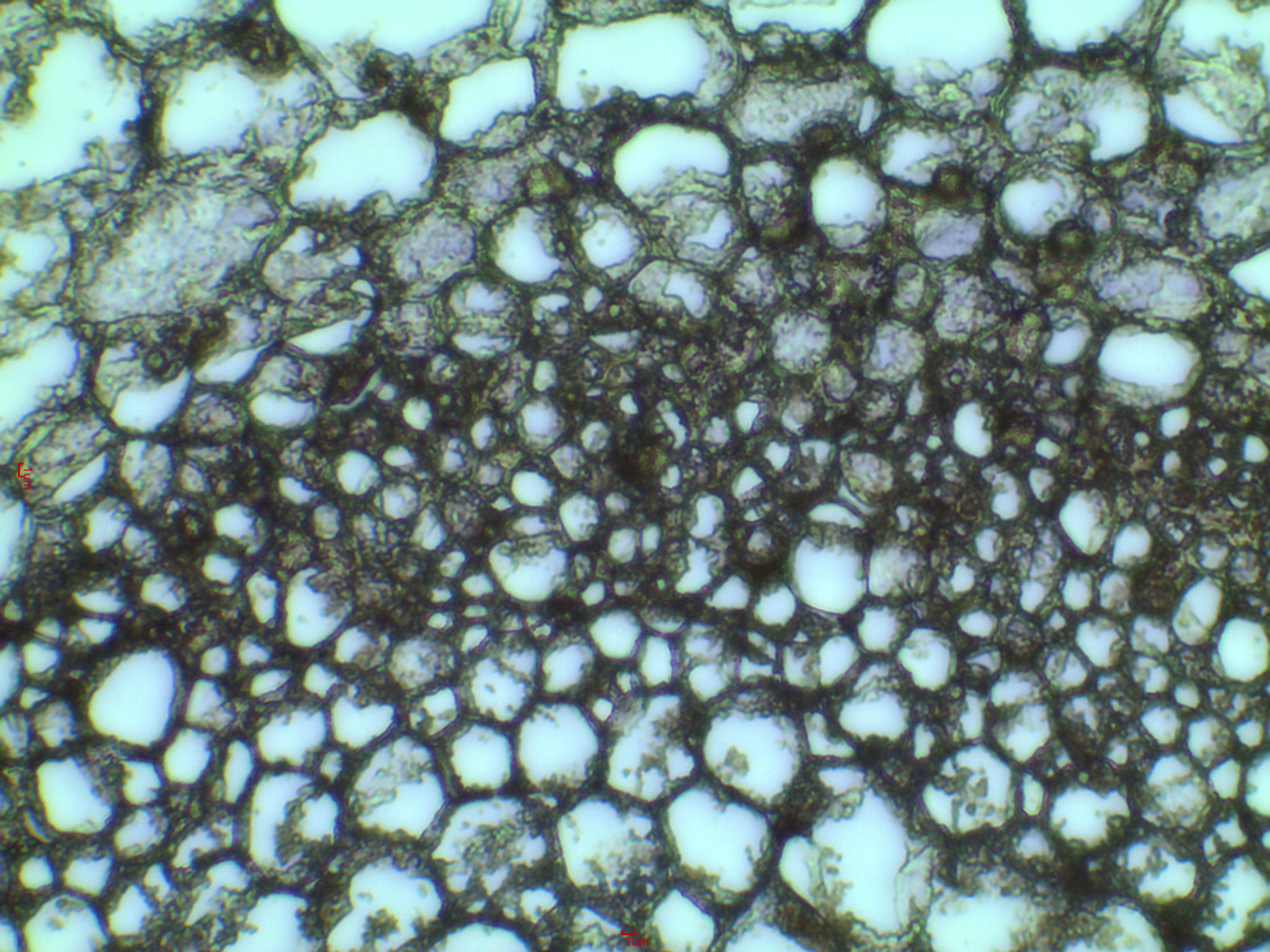

Supplement: S8 Supporting Information — (ZIP) [file pone.0218513.s012.zip › S8_Supporting Information.zip/E.jpg]

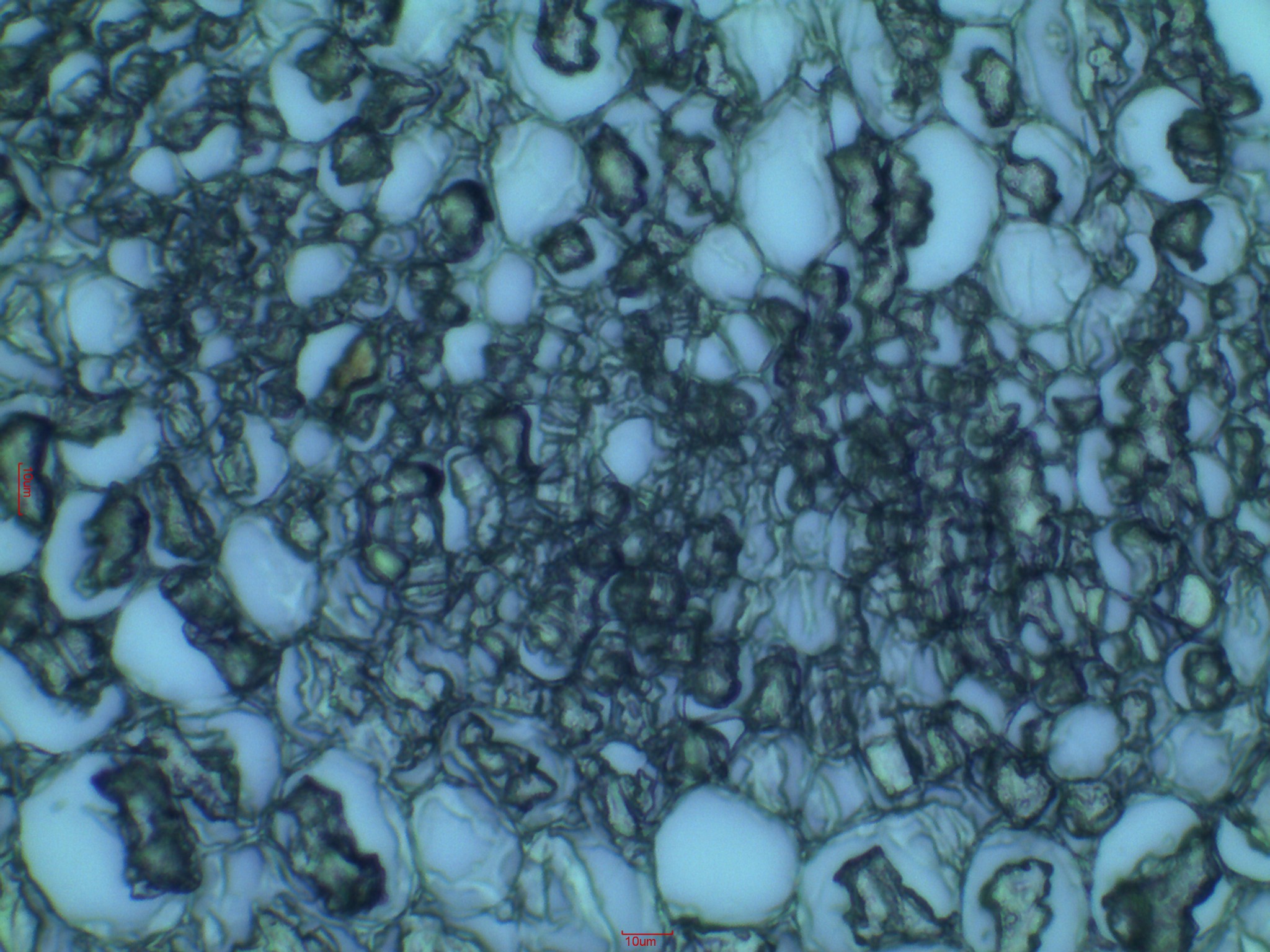

Supplement: S8 Supporting Information — (ZIP) [file pone.0218513.s012.zip › S8_Supporting Information.zip/F.jpg]

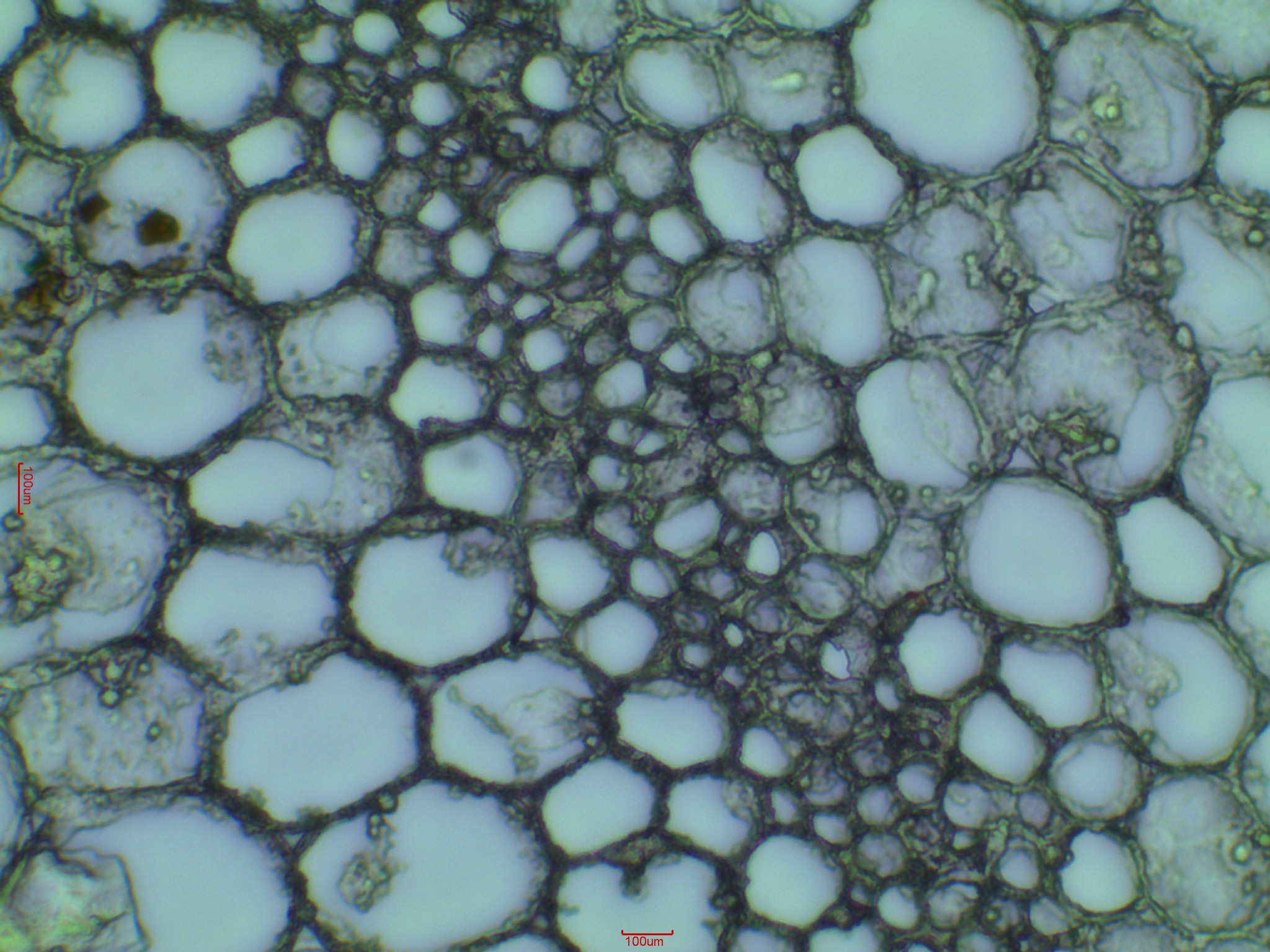

Supplement: S8 Supporting Information — (ZIP) [file pone.0218513.s012.zip › S8_Supporting Information.zip/G.jpg]

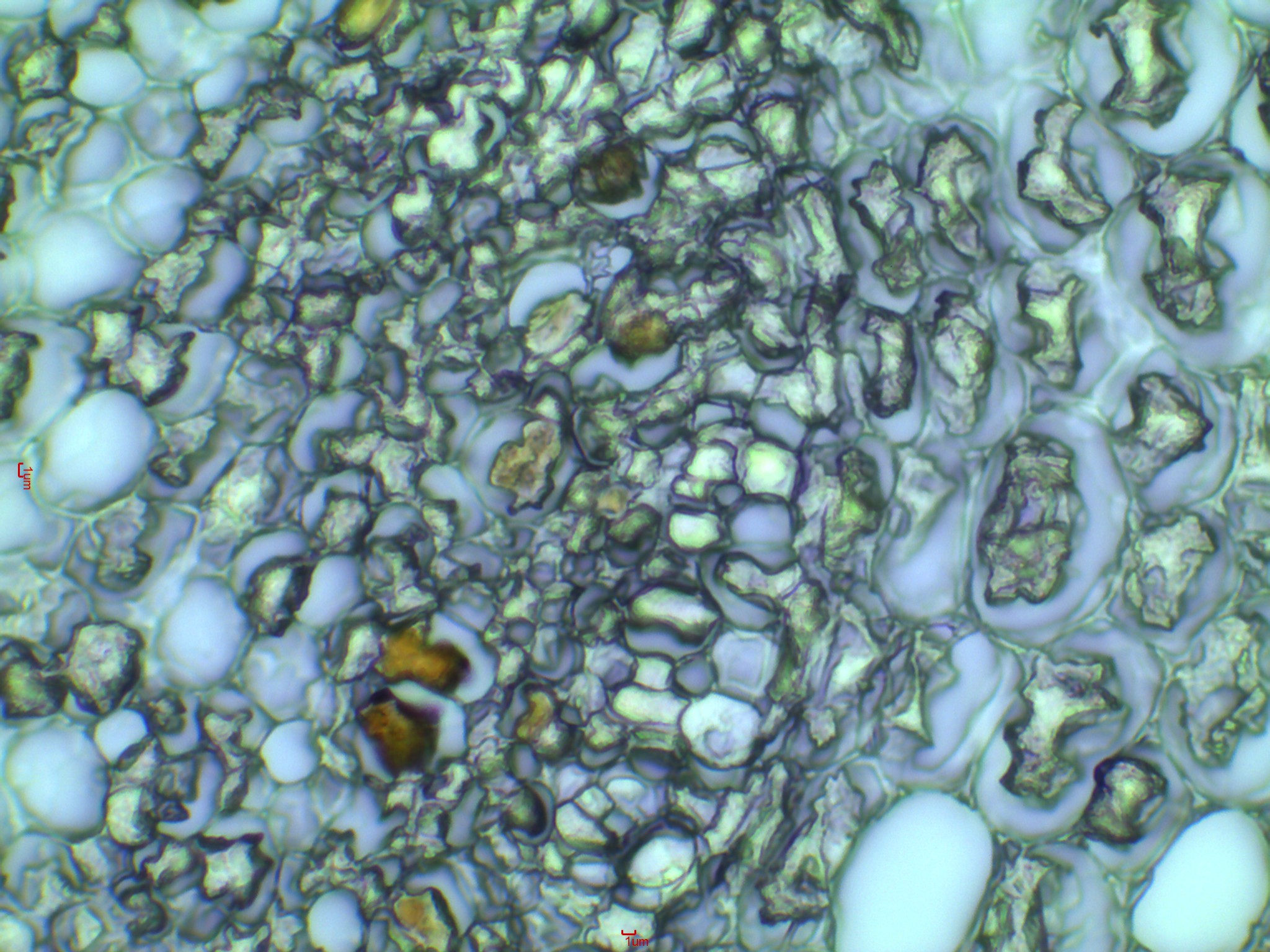

Supplement: S8 Supporting Information — (ZIP) [file pone.0218513.s012.zip › S8_Supporting Information.zip/H.jpg]

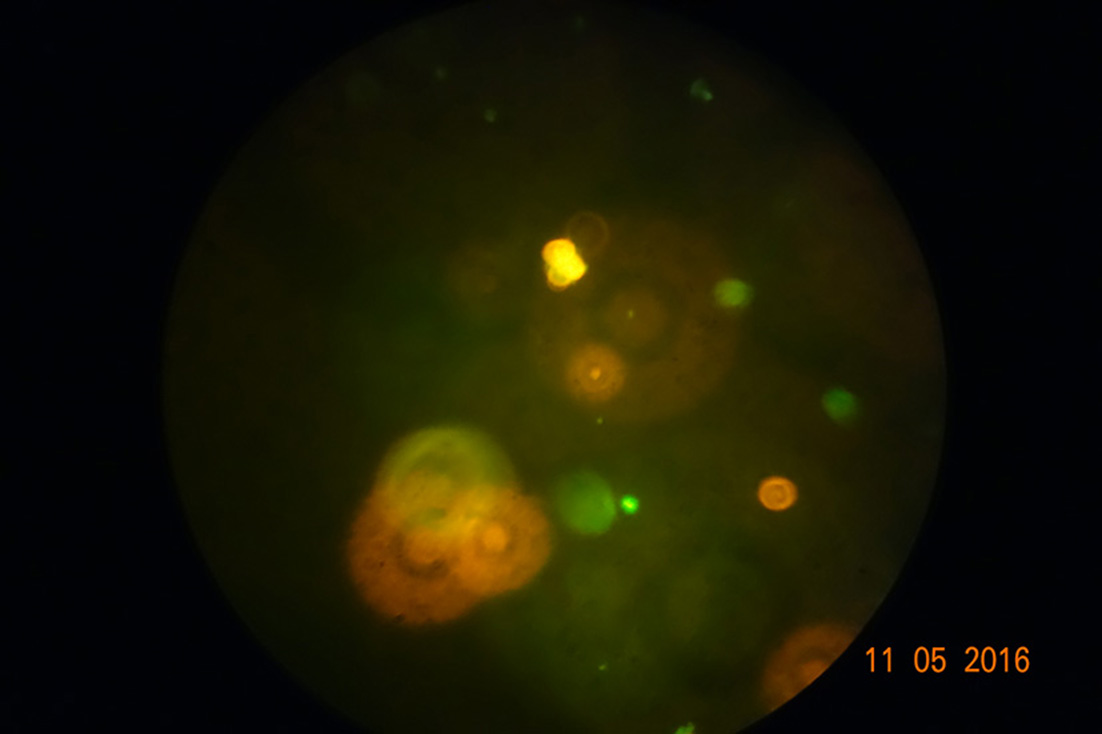

Supplement: S11 Supporting Information — (ZIP) [file pone.0218513.s015.zip › S11_Supporting Information.zip/105MSDCF/DSC05038.JPG]

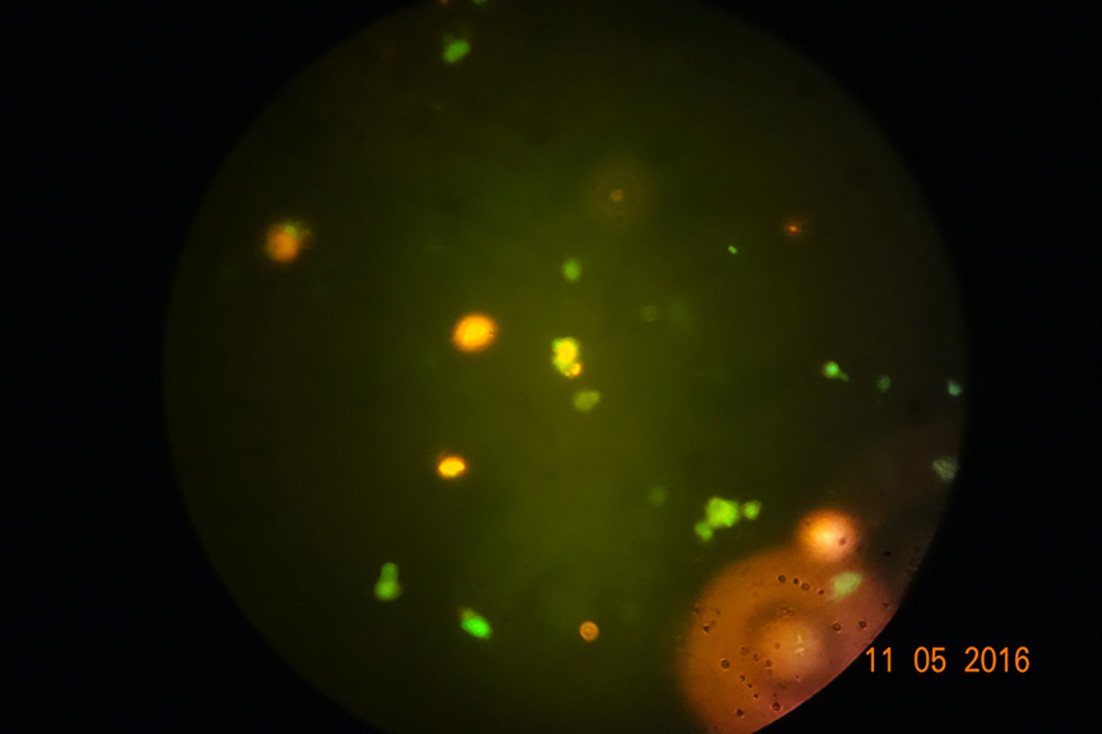

Supplement: S11 Supporting Information — (ZIP) [file pone.0218513.s015.zip › S11_Supporting Information.zip/105MSDCF/DSC05039.JPG]

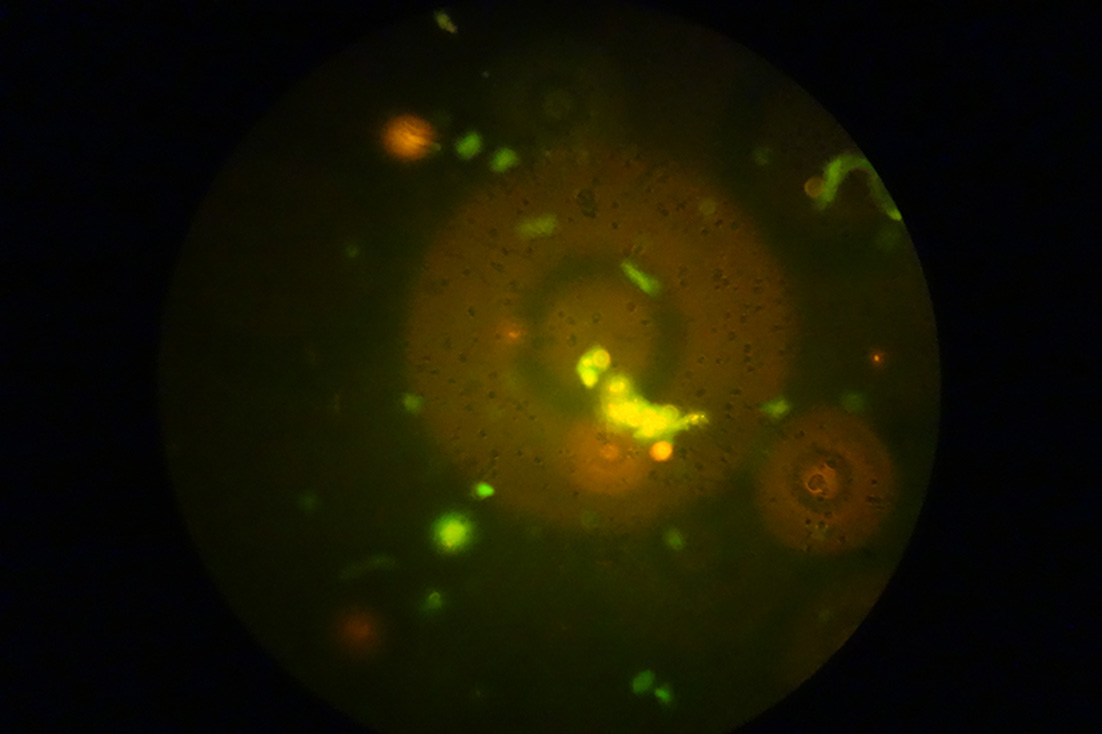

Supplement: S11 Supporting Information — (ZIP) [file pone.0218513.s015.zip › S11_Supporting Information.zip/105MSDCF/DSC05040.JPG]

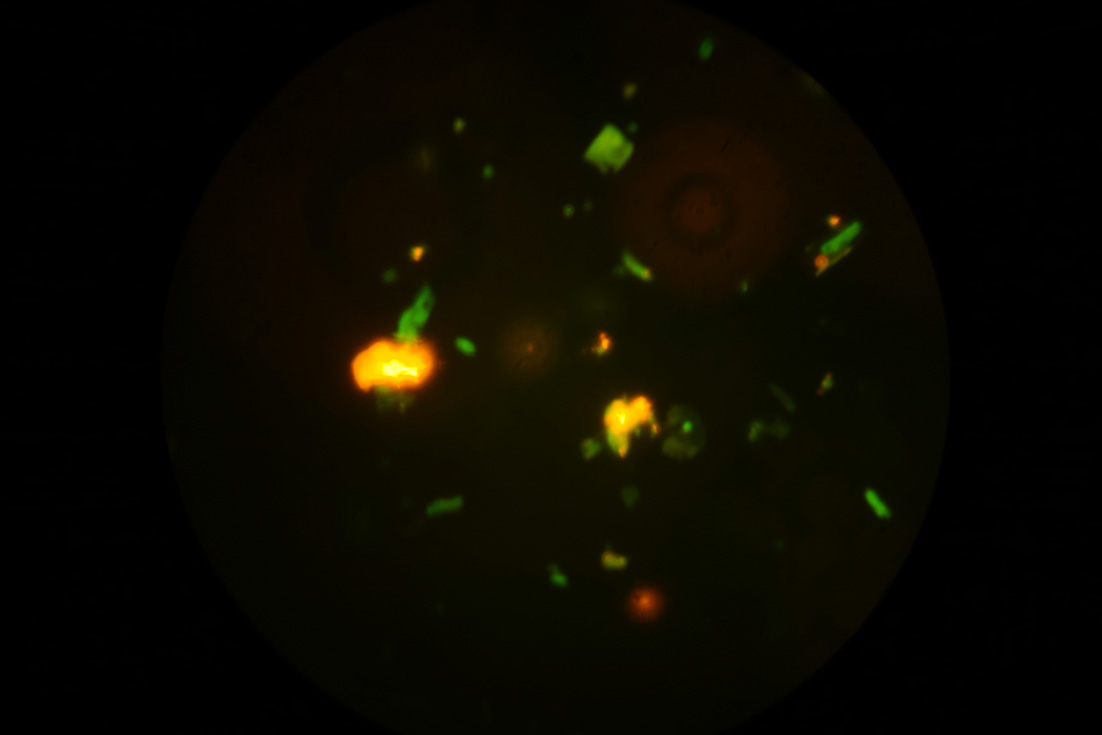

Supplement: S11 Supporting Information — (ZIP) [file pone.0218513.s015.zip › S11_Supporting Information.zip/105MSDCF/DSC05041.JPG]

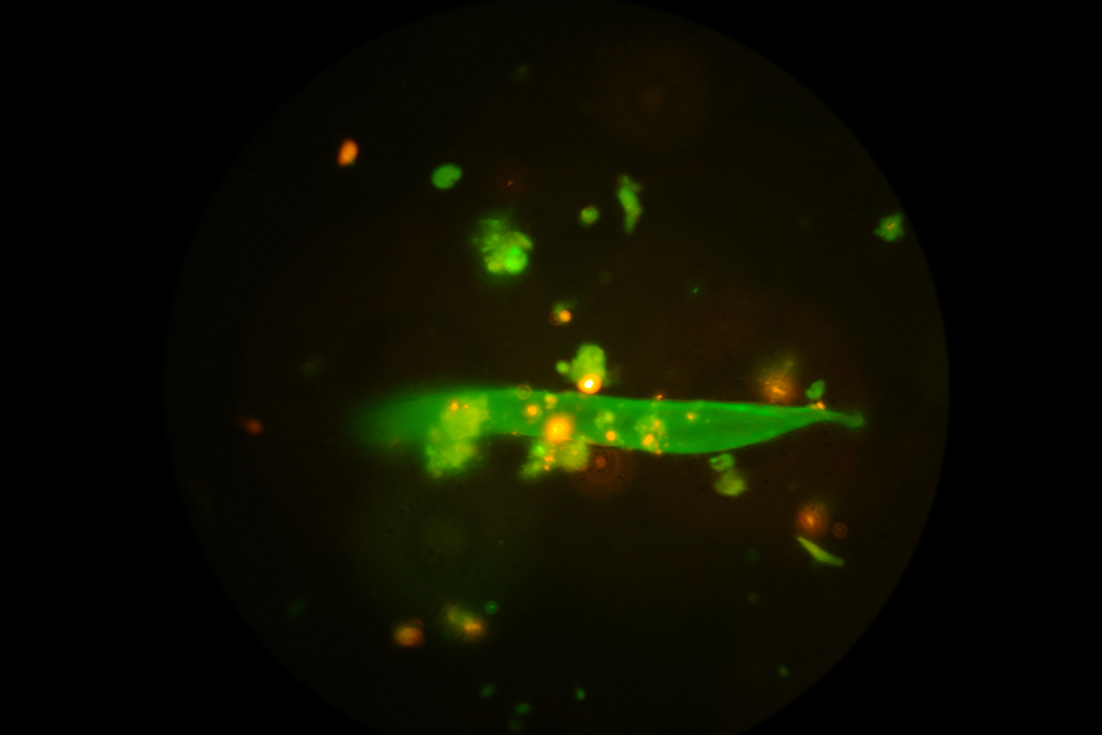

Supplement: S11 Supporting Information — (ZIP) [file pone.0218513.s015.zip › S11_Supporting Information.zip/105MSDCF/DSC05042.JPG]

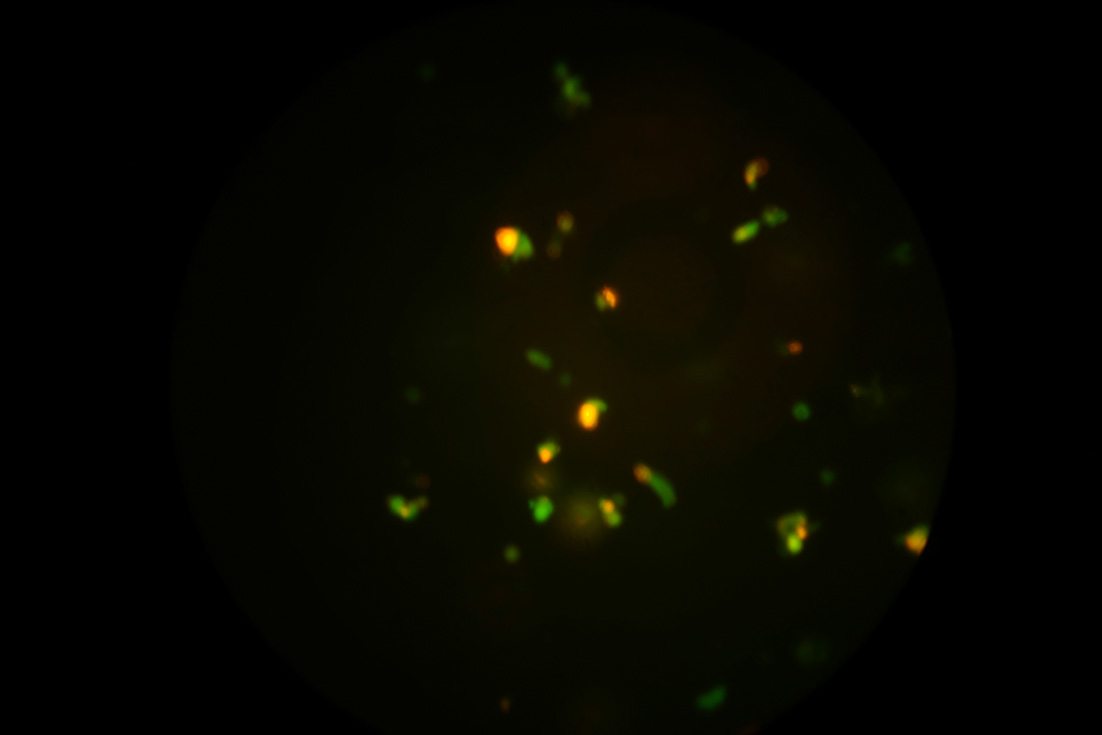

Supplement: S11 Supporting Information — (ZIP) [file pone.0218513.s015.zip › S11_Supporting Information.zip/105MSDCF/DSC05043.JPG]

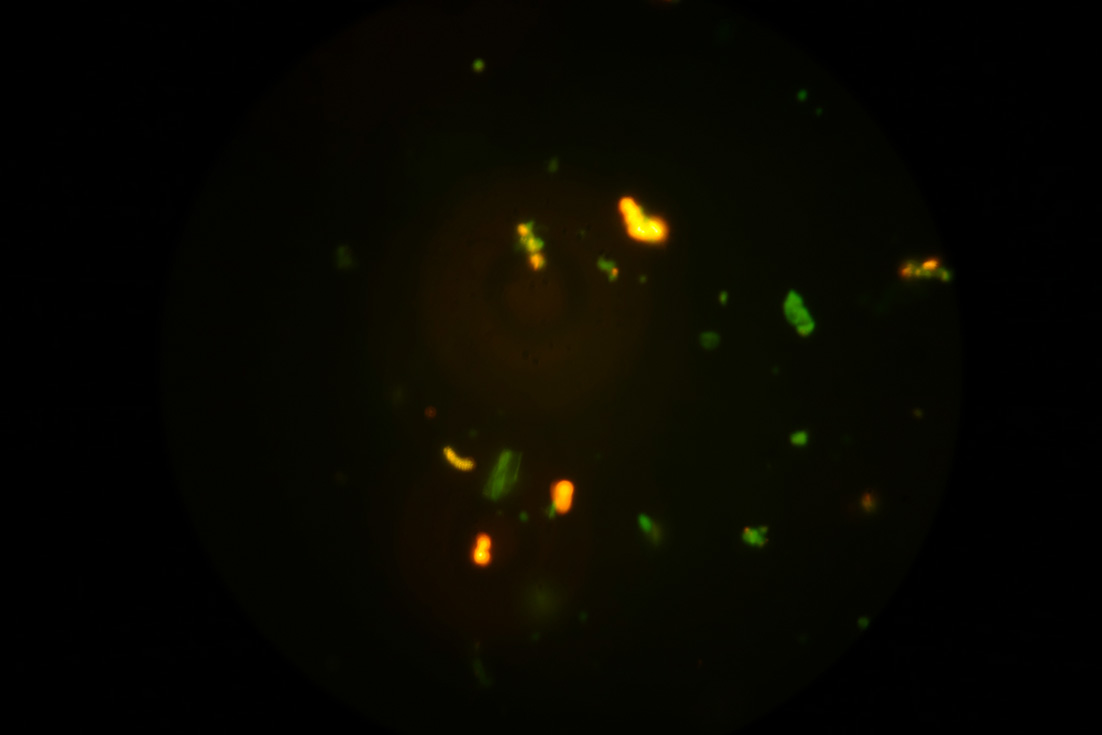

Supplement: S11 Supporting Information — (ZIP) [file pone.0218513.s015.zip › S11_Supporting Information.zip/105MSDCF/DSC05044.JPG]

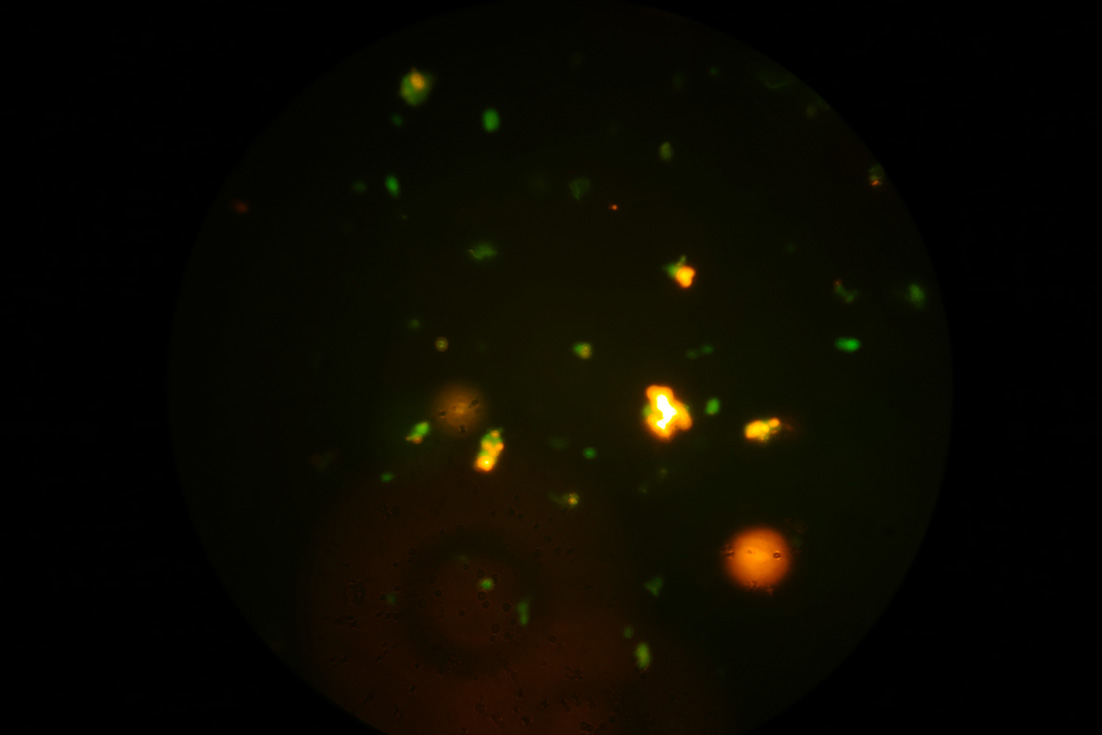

Supplement: S11 Supporting Information — (ZIP) [file pone.0218513.s015.zip › S11_Supporting Information.zip/105MSDCF/DSC05045.JPG]

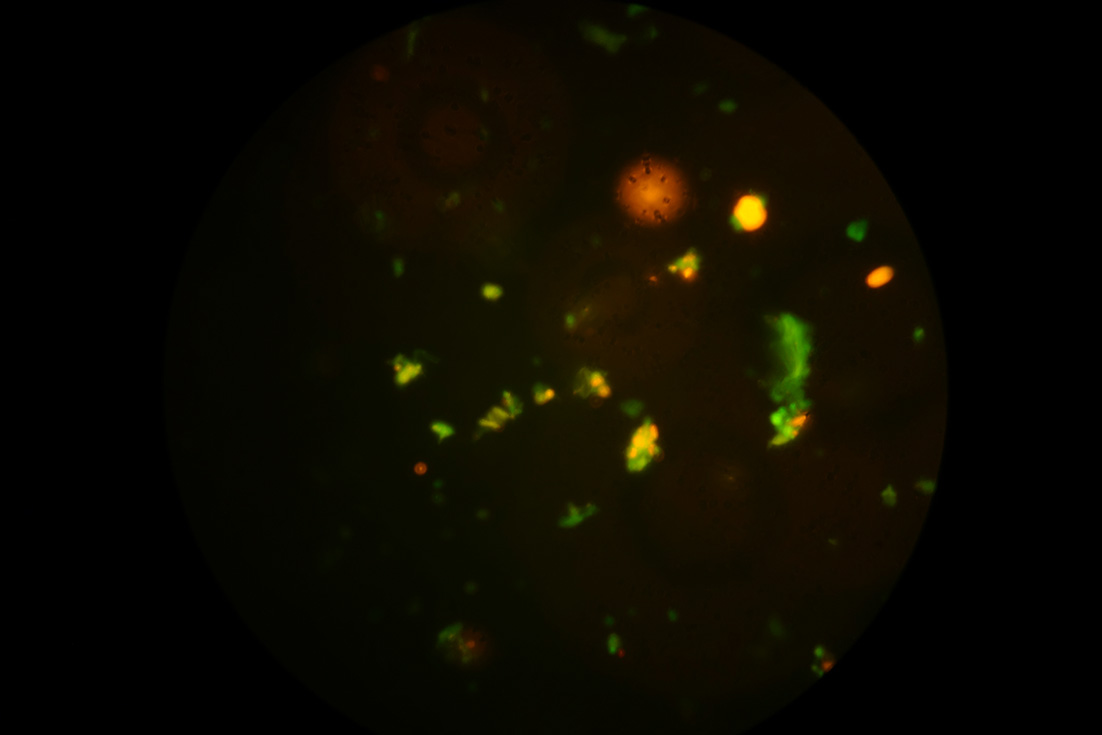

Supplement: S11 Supporting Information — (ZIP) [file pone.0218513.s015.zip › S11_Supporting Information.zip/105MSDCF/DSC05046.JPG]

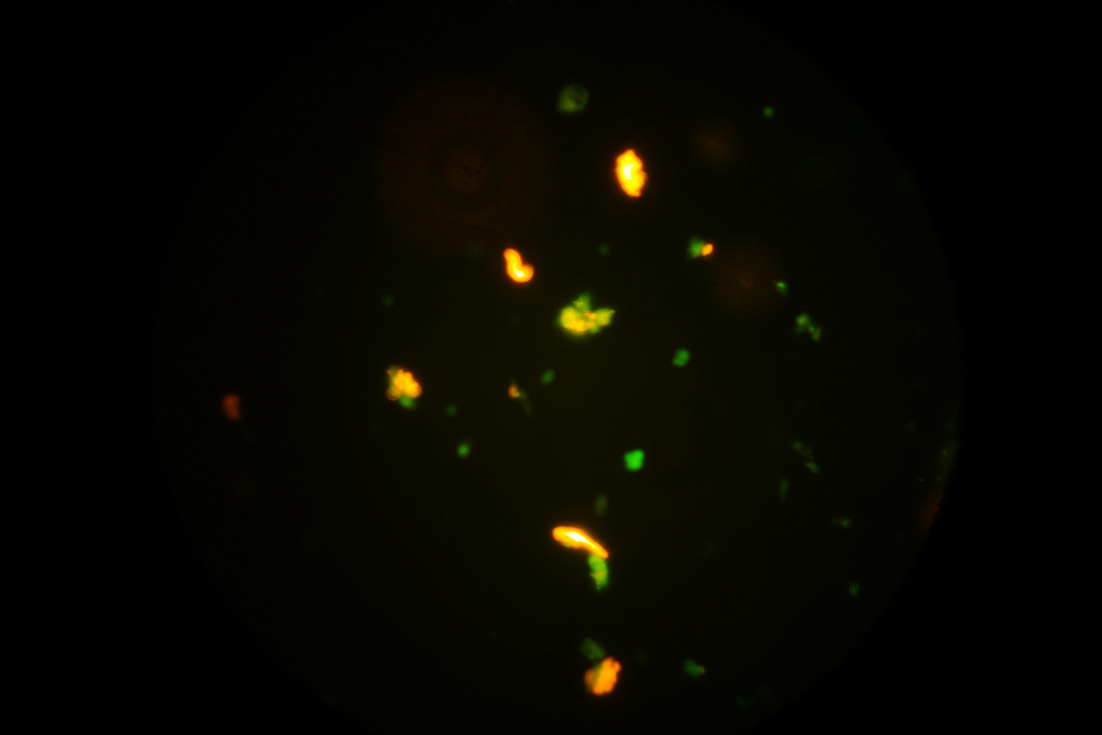

Supplement: S11 Supporting Information — (ZIP) [file pone.0218513.s015.zip › S11_Supporting Information.zip/105MSDCF/DSC05047.JPG]

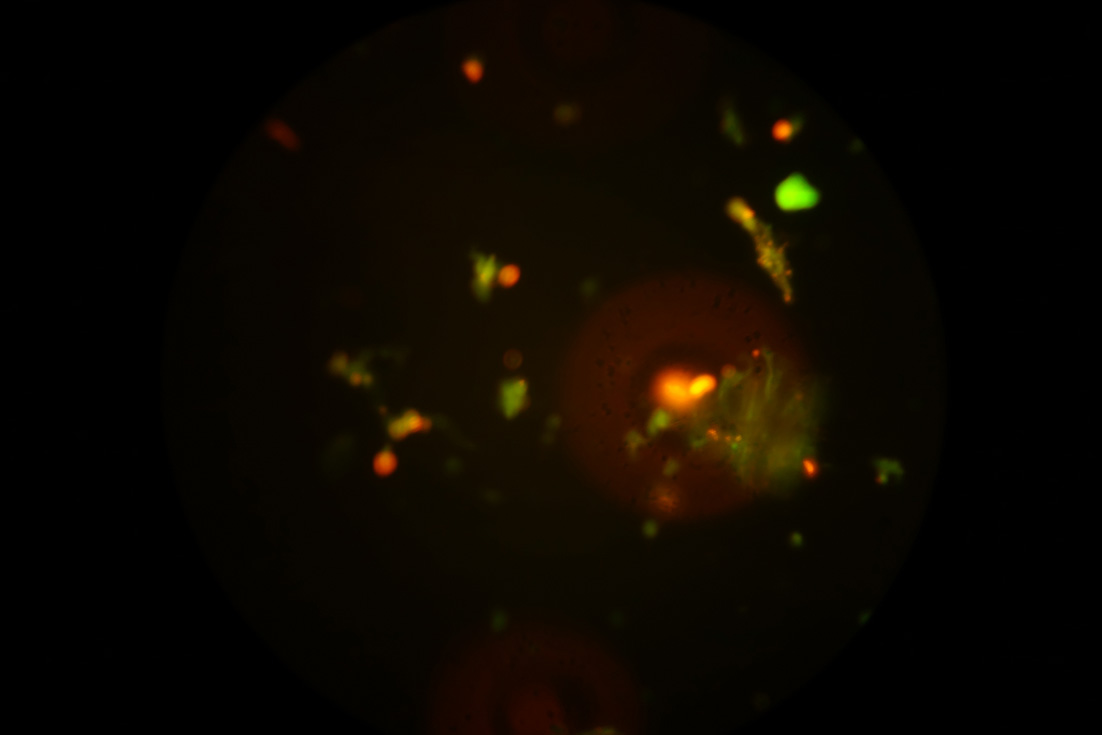

Supplement: S11 Supporting Information — (ZIP) [file pone.0218513.s015.zip › S11_Supporting Information.zip/105MSDCF/DSC05048.JPG]

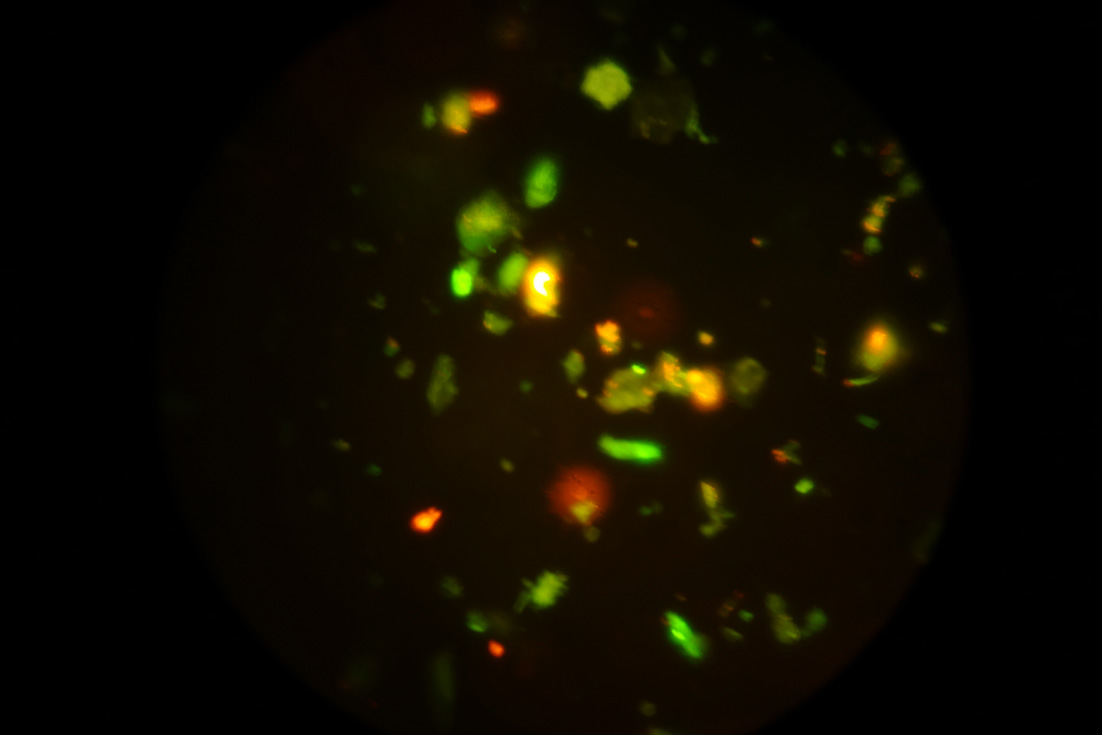

Supplement: S11 Supporting Information — (ZIP) [file pone.0218513.s015.zip › S11_Supporting Information.zip/105MSDCF/DSC05049.JPG]

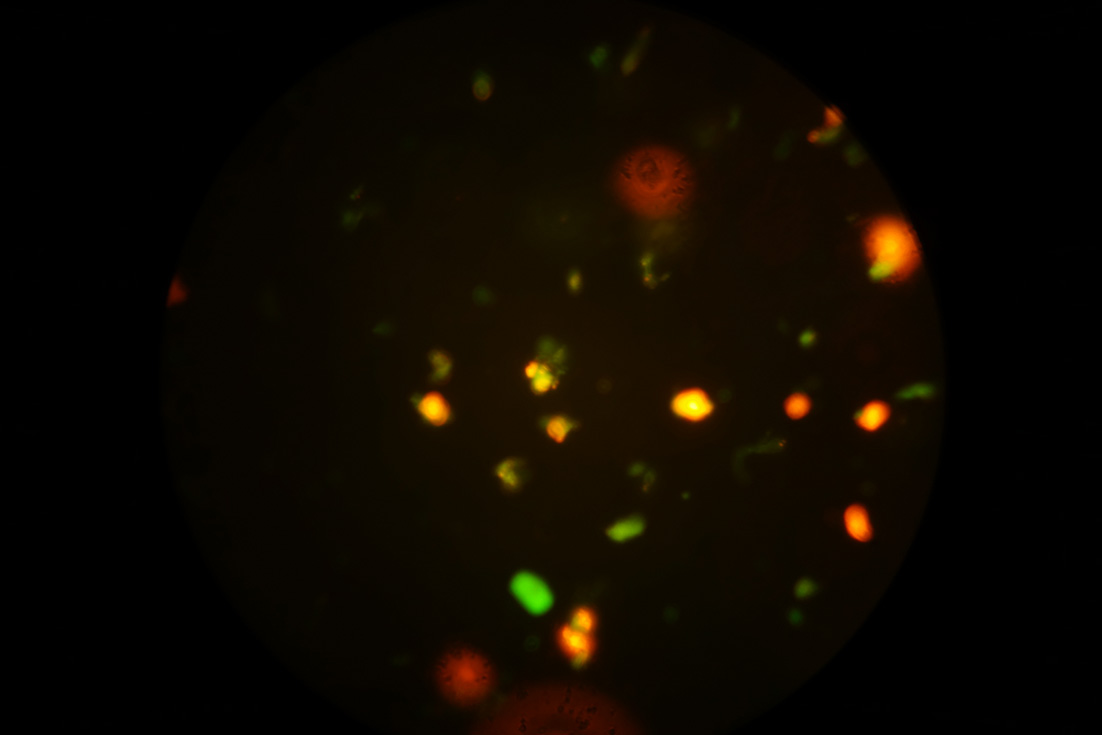

Supplement: S11 Supporting Information — (ZIP) [file pone.0218513.s015.zip › S11_Supporting Information.zip/105MSDCF/DSC05050.JPG]

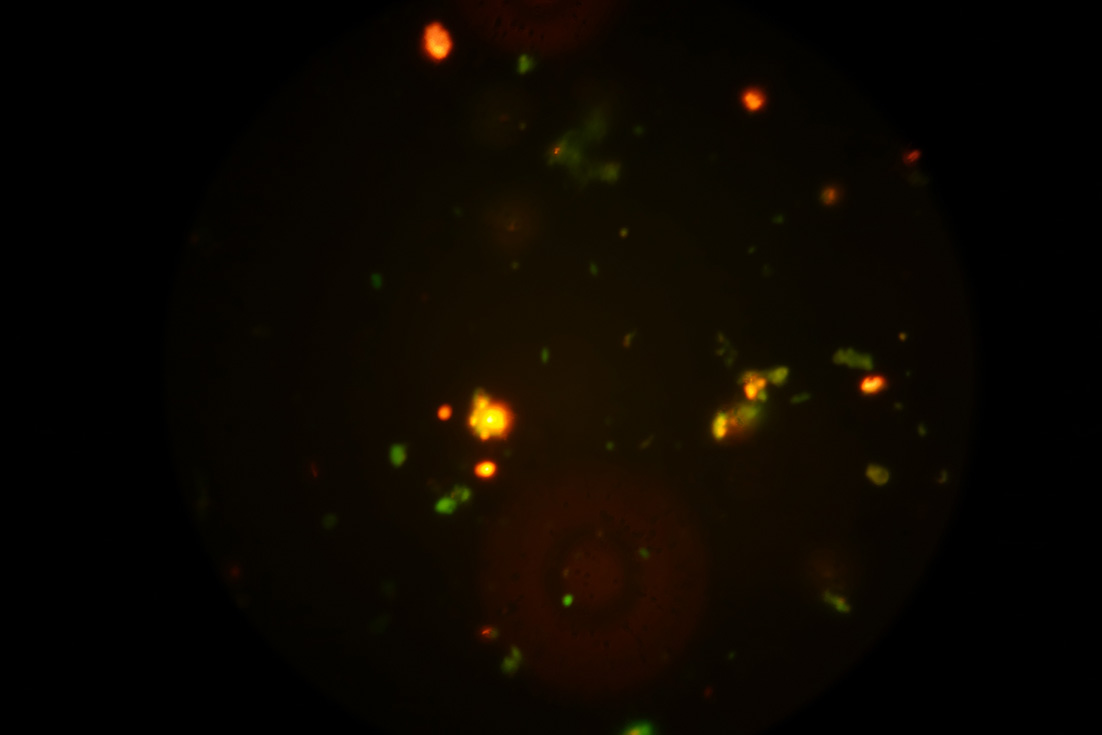

Supplement: S11 Supporting Information — (ZIP) [file pone.0218513.s015.zip › S11_Supporting Information.zip/105MSDCF/DSC05051.JPG]

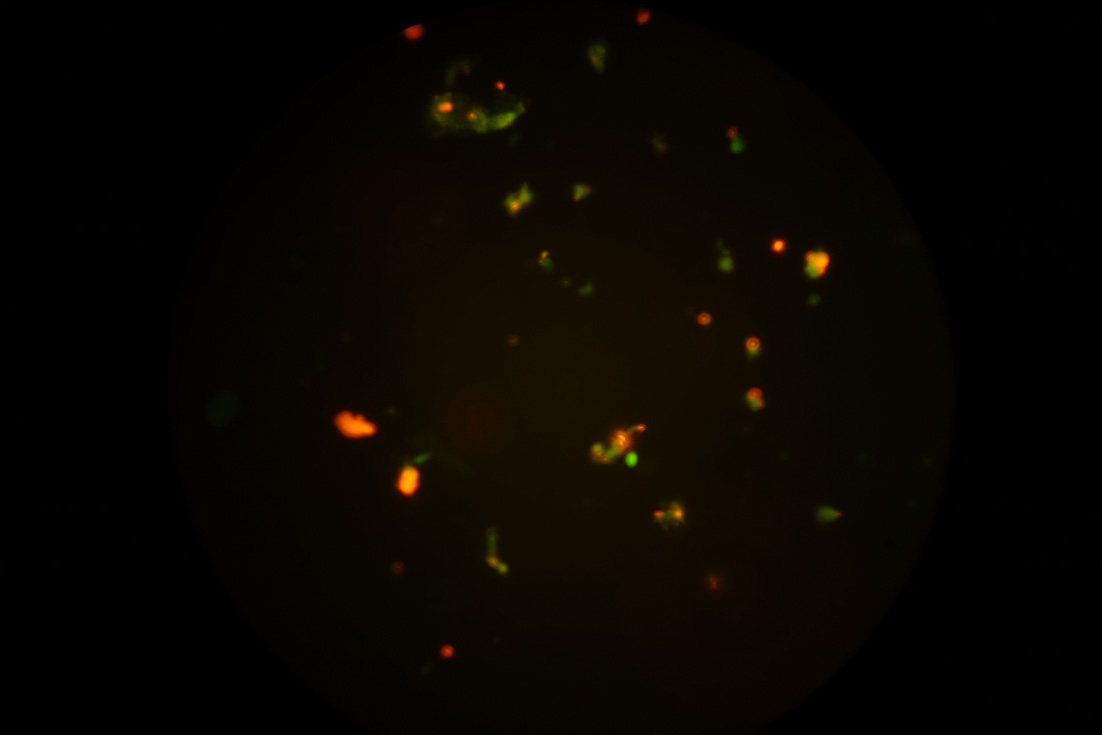

Supplement: S11 Supporting Information — (ZIP) [file pone.0218513.s015.zip › S11_Supporting Information.zip/105MSDCF/DSC05052.JPG]

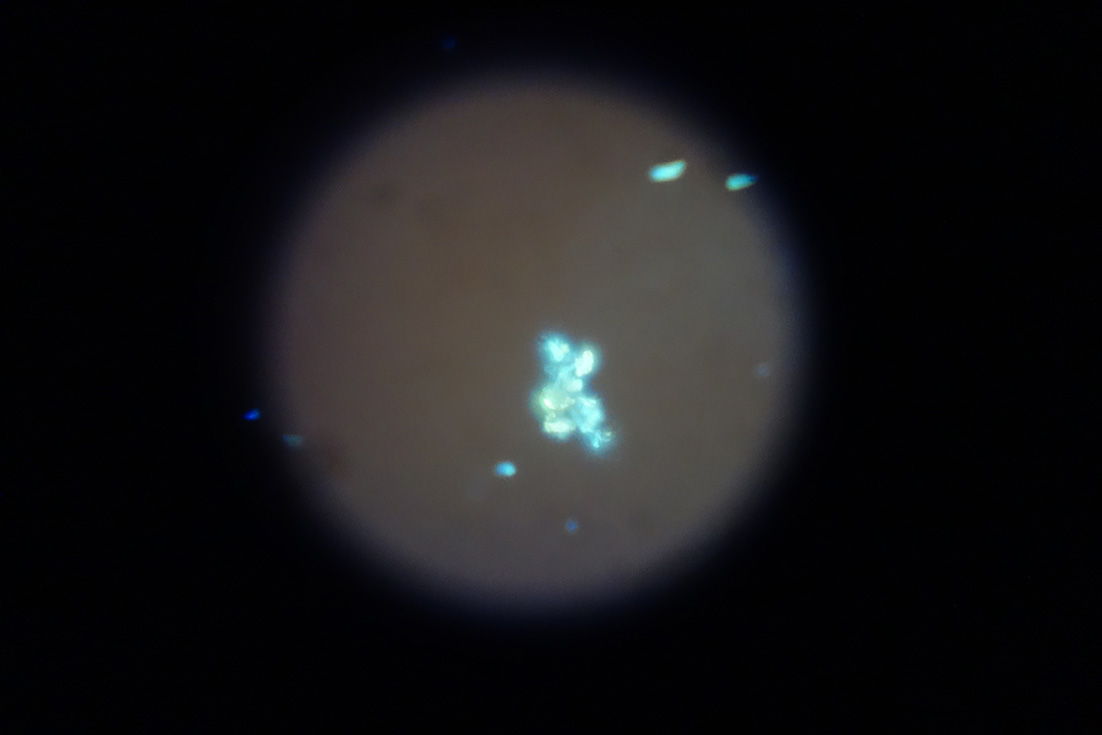

Supplement: S11 Supporting Information — (ZIP) [file pone.0218513.s015.zip › S11_Supporting Information.zip/cccp/DSC05019.JPG]

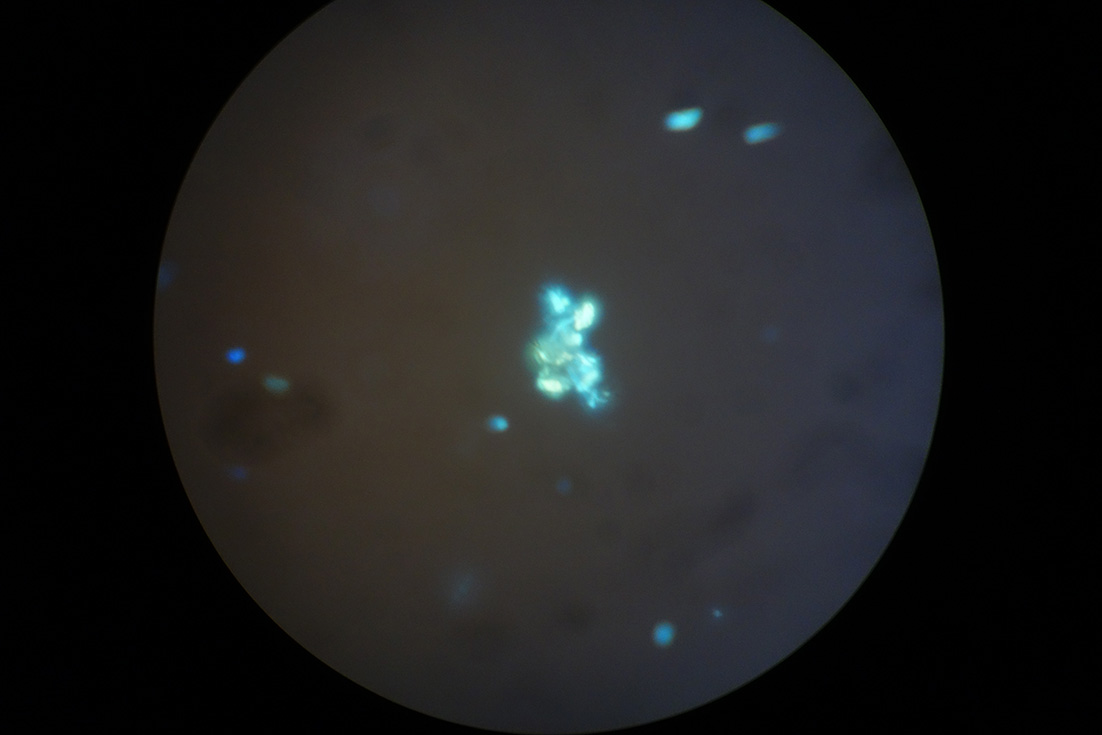

Supplement: S11 Supporting Information — (ZIP) [file pone.0218513.s015.zip › S11_Supporting Information.zip/cccp/DSC05020.JPG]

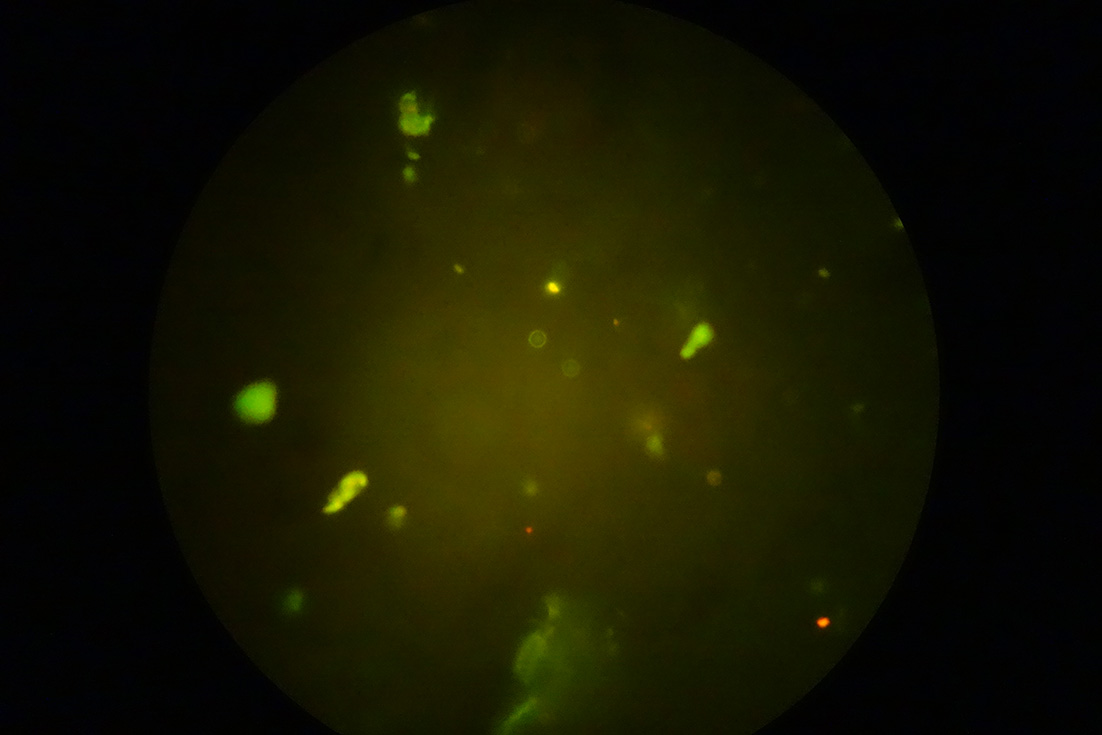

Supplement: S11 Supporting Information — (ZIP) [file pone.0218513.s015.zip › S11_Supporting Information.zip/cccp/DSC05021.JPG]

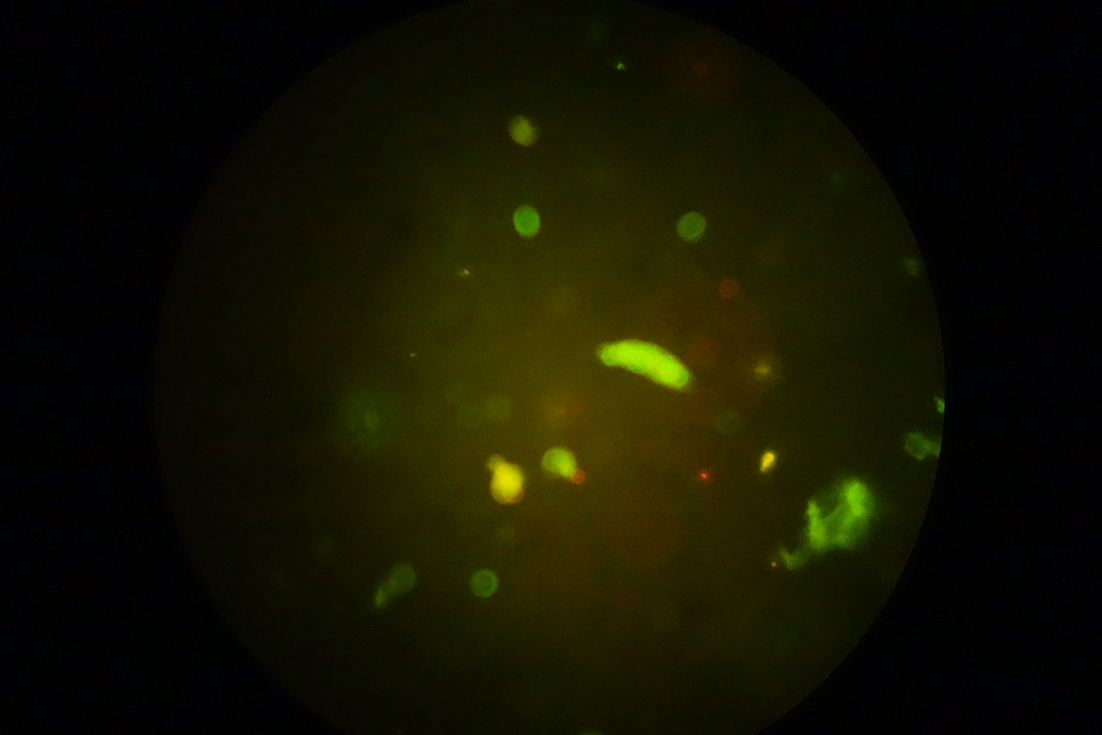

Supplement: S11 Supporting Information — (ZIP) [file pone.0218513.s015.zip › S11_Supporting Information.zip/cccp/DSC05022.JPG]

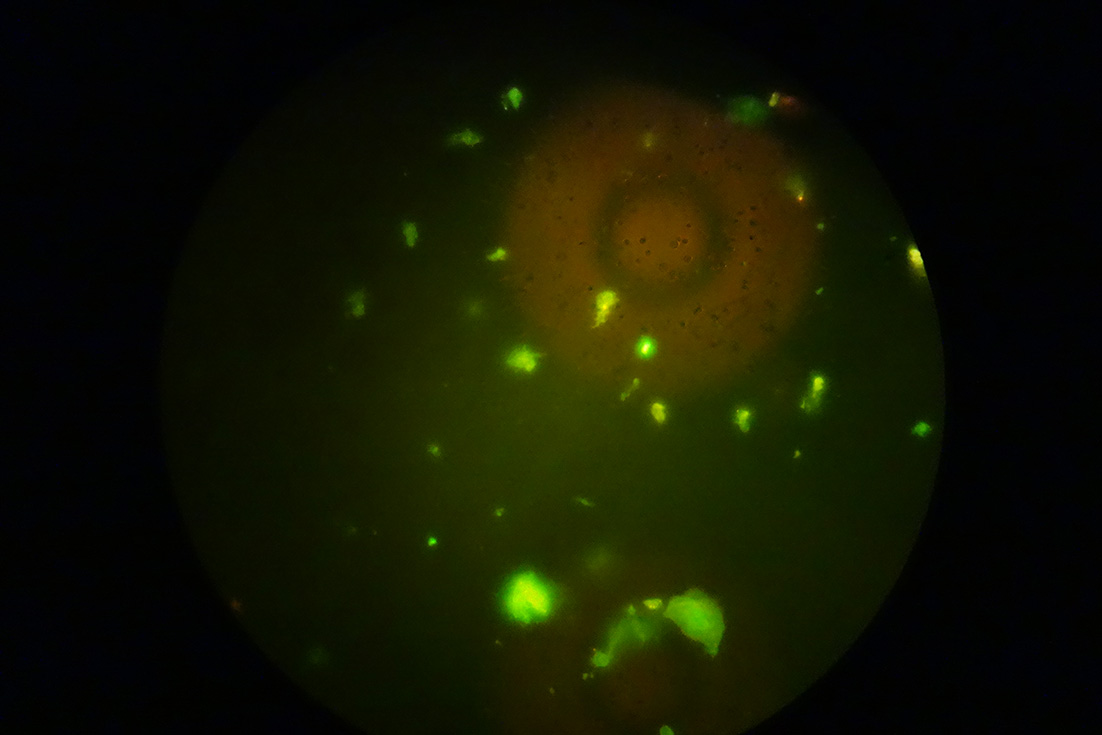

Supplement: S11 Supporting Information — (ZIP) [file pone.0218513.s015.zip › S11_Supporting Information.zip/cccp/DSC05023.JPG]

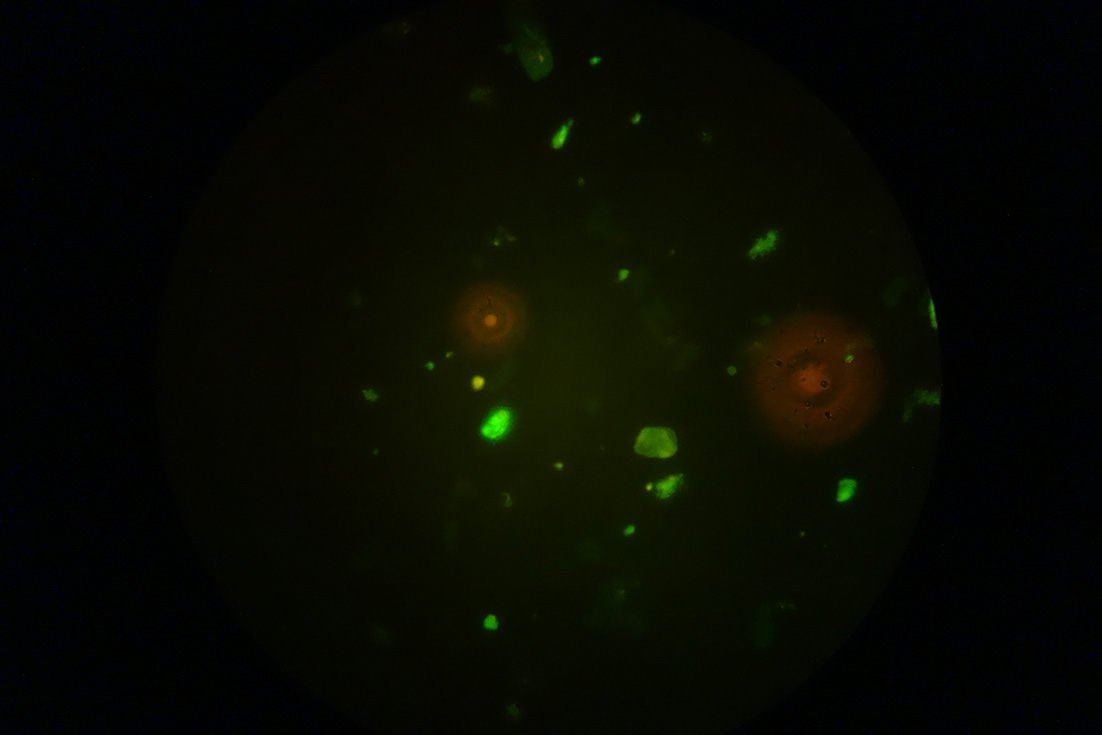

Supplement: S11 Supporting Information — (ZIP) [file pone.0218513.s015.zip › S11_Supporting Information.zip/cccp/DSC05025.JPG]

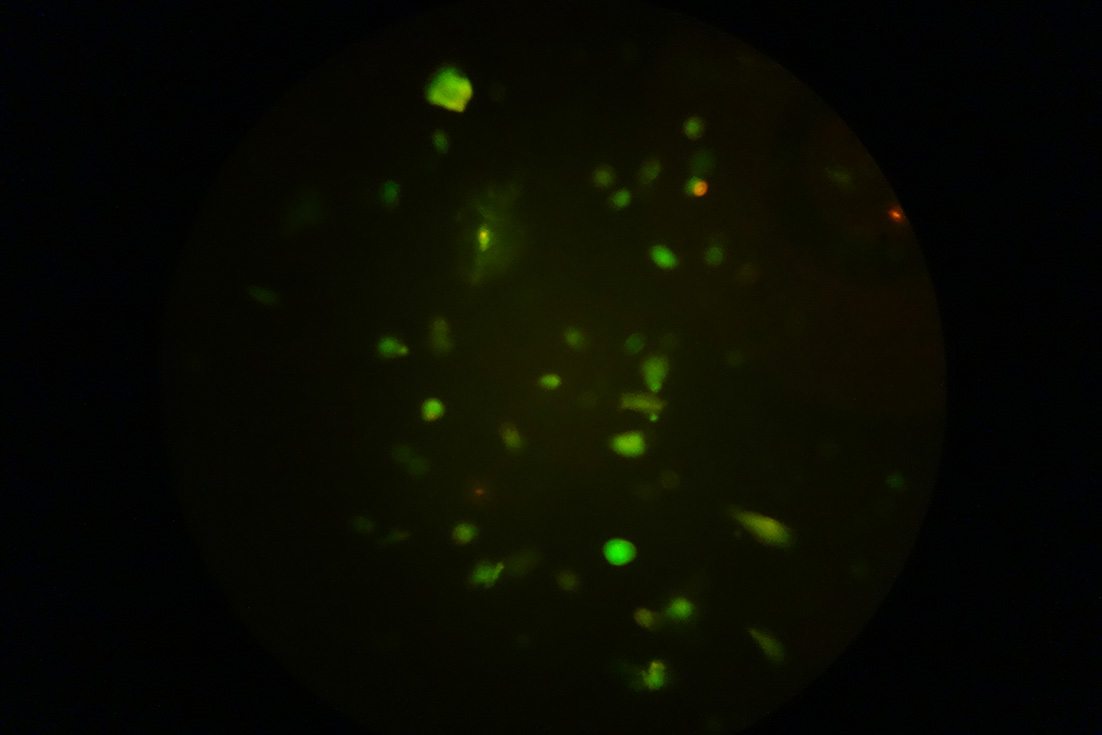

Supplement: S11 Supporting Information — (ZIP) [file pone.0218513.s015.zip › S11_Supporting Information.zip/cccp/DSC05026.JPG]

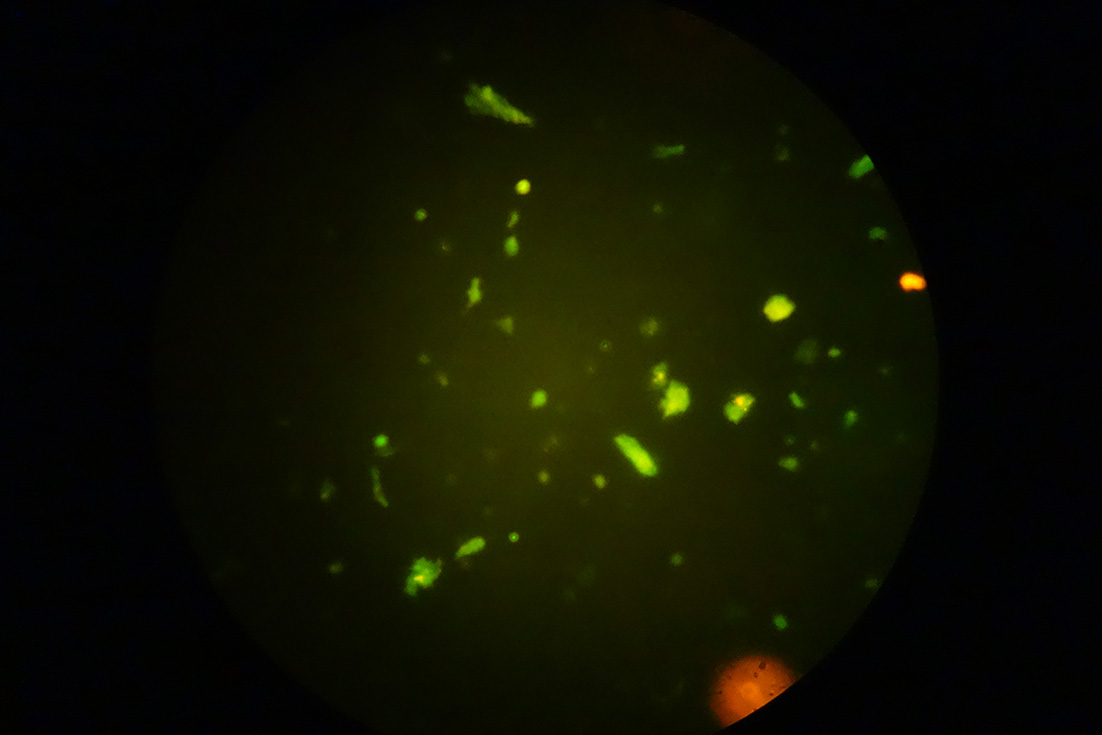

Supplement: S11 Supporting Information — (ZIP) [file pone.0218513.s015.zip › S11_Supporting Information.zip/cccp/DSC05027.JPG]

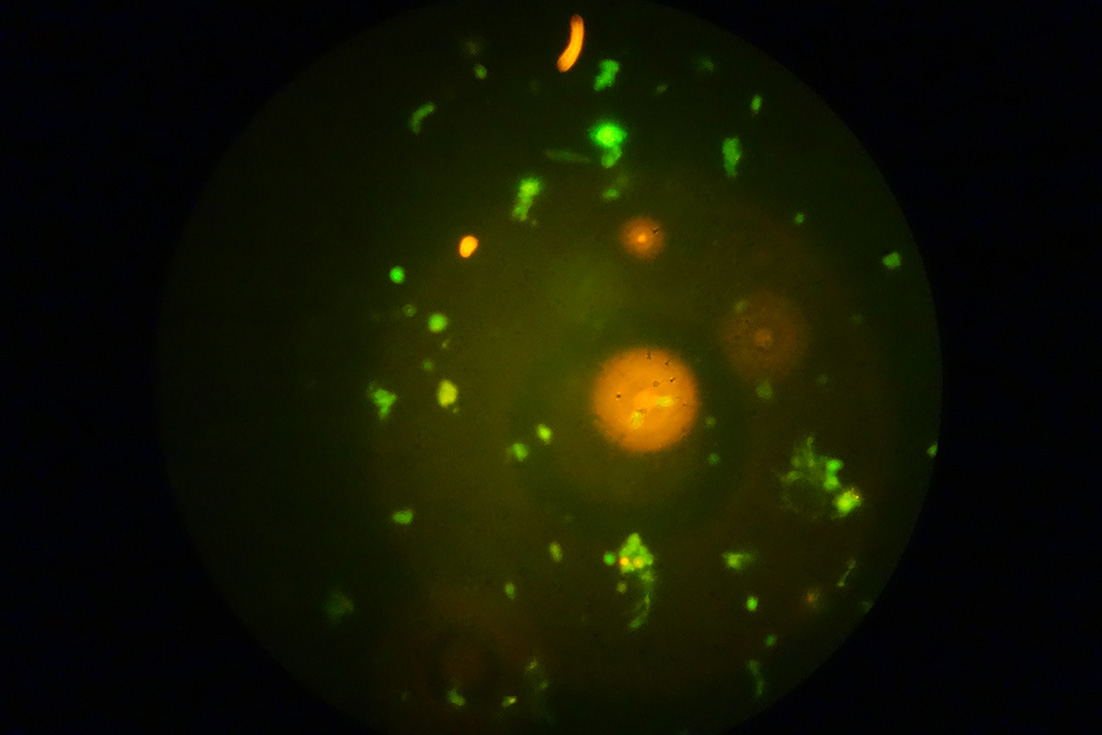

Supplement: S11 Supporting Information — (ZIP) [file pone.0218513.s015.zip › S11_Supporting Information.zip/cccp/DSC05028.JPG]

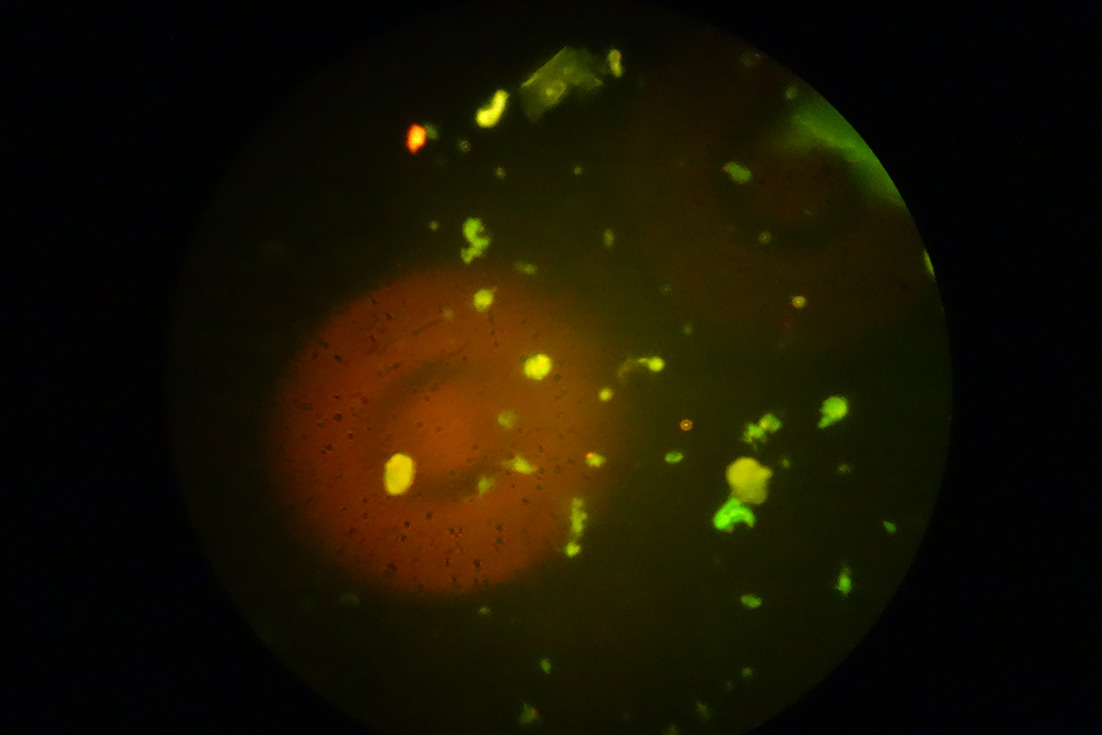

Supplement: S11 Supporting Information — (ZIP) [file pone.0218513.s015.zip › S11_Supporting Information.zip/cccp/DSC05029.JPG]

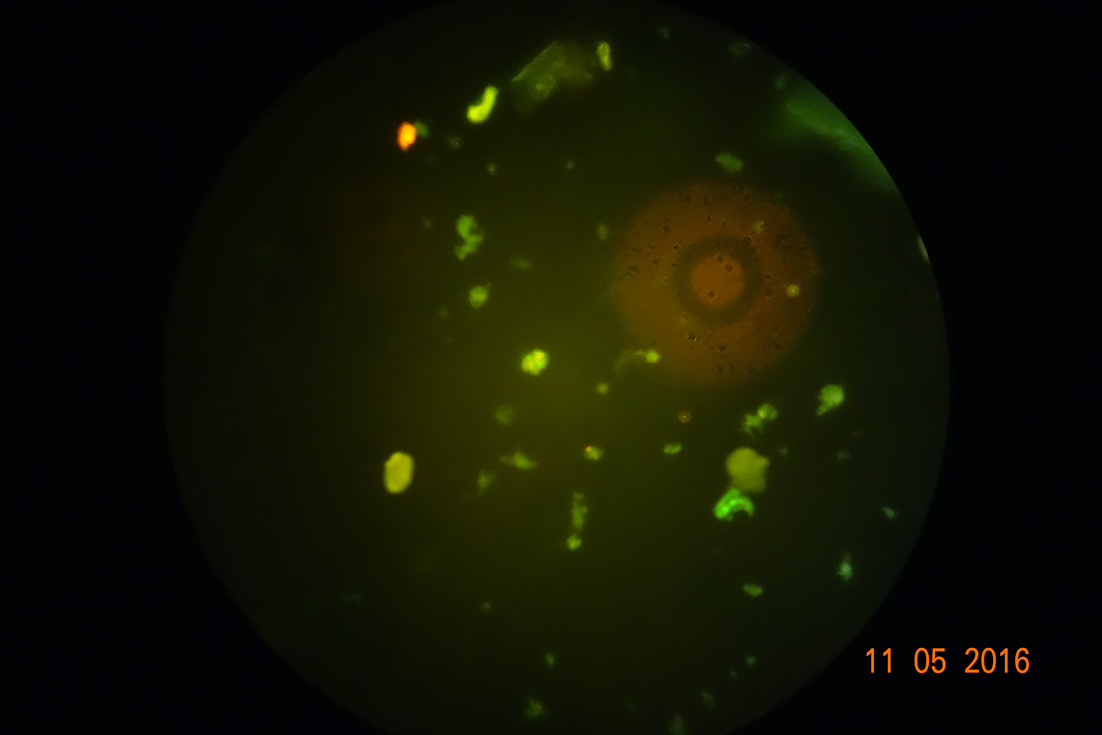

Supplement: S11 Supporting Information — (ZIP) [file pone.0218513.s015.zip › S11_Supporting Information.zip/cccp/DSC05030.JPG]

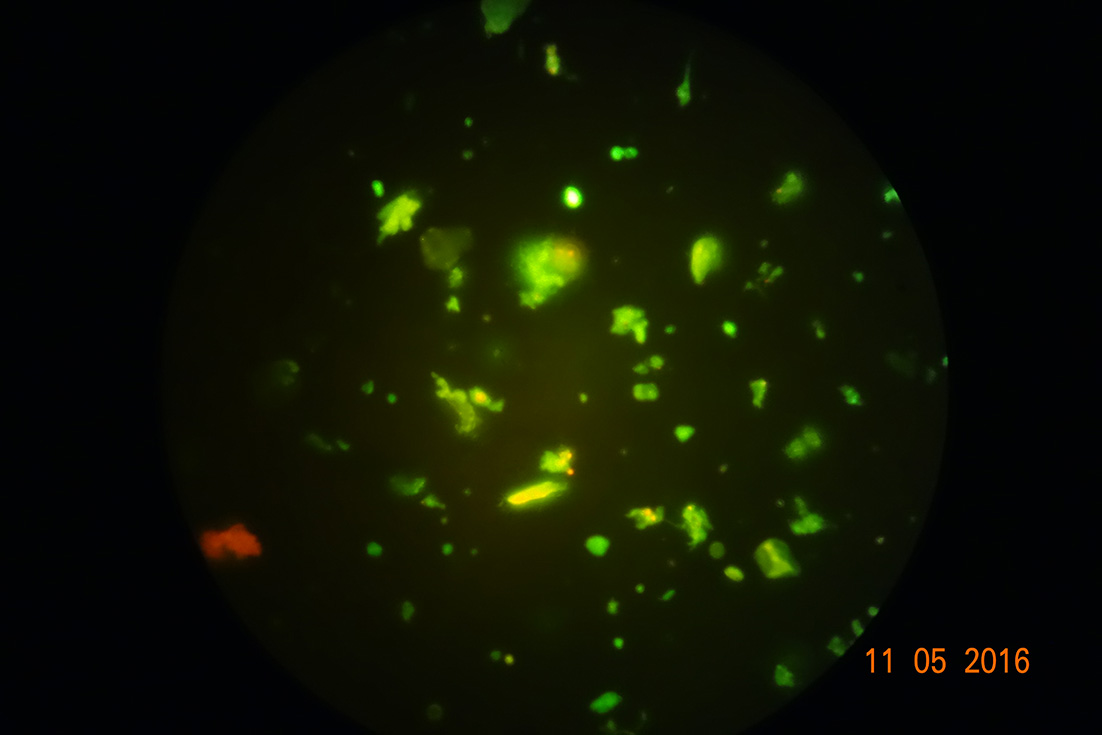

Supplement: S11 Supporting Information — (ZIP) [file pone.0218513.s015.zip › S11_Supporting Information.zip/cccp/DSC05031.JPG]

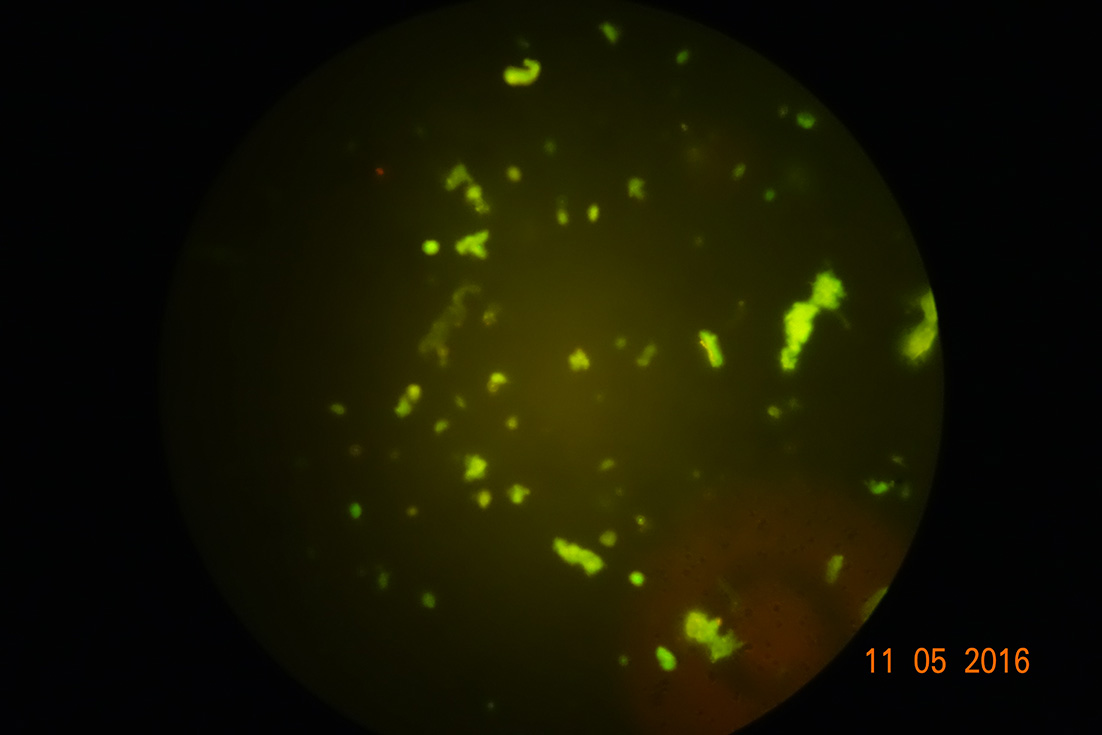

Supplement: S11 Supporting Information — (ZIP) [file pone.0218513.s015.zip › S11_Supporting Information.zip/cccp/DSC05032.JPG]

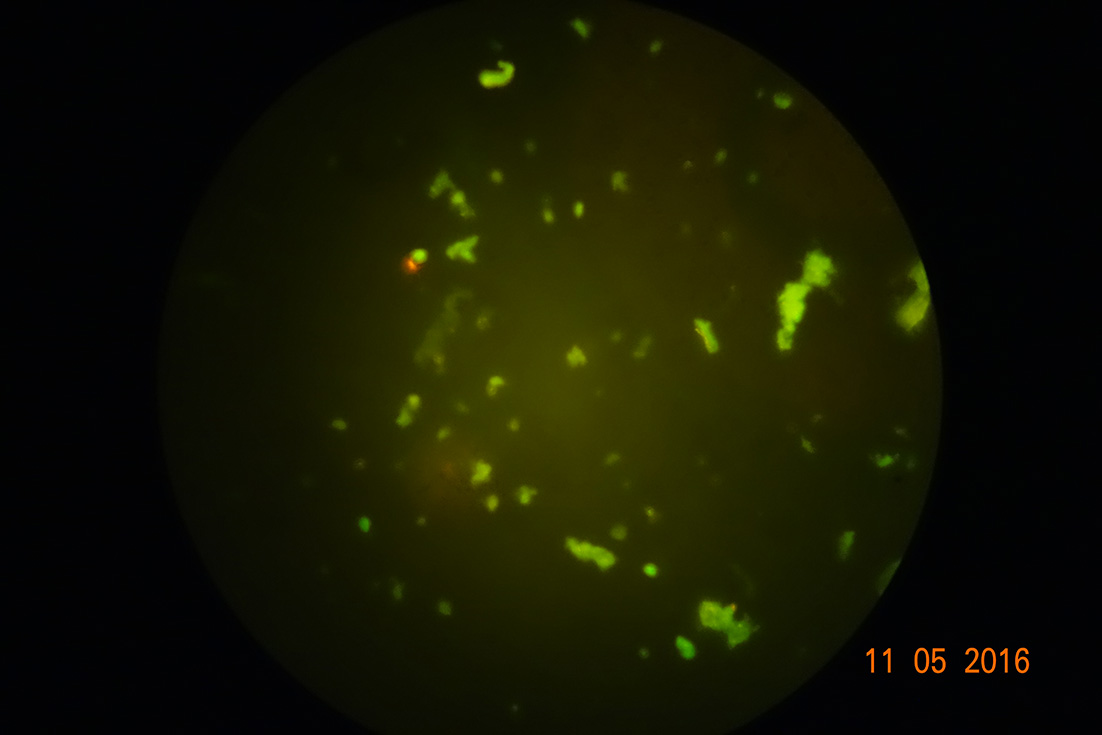

Supplement: S11 Supporting Information — (ZIP) [file pone.0218513.s015.zip › S11_Supporting Information.zip/cccp/DSC05033.JPG]
