# Supplementary figures and images for: Relationship between loss of desiccation tolerance and programmed cell death (PCD) in mung bean (Vigna radiata) seeds (part 2 of 2)
Source: PLoS One. 2019 Jul 2;14(7):e0218513. doi: 10.1371/journal.pone.0218513 (PMC6605718; doi:10.1371/journal.pone.0218513)

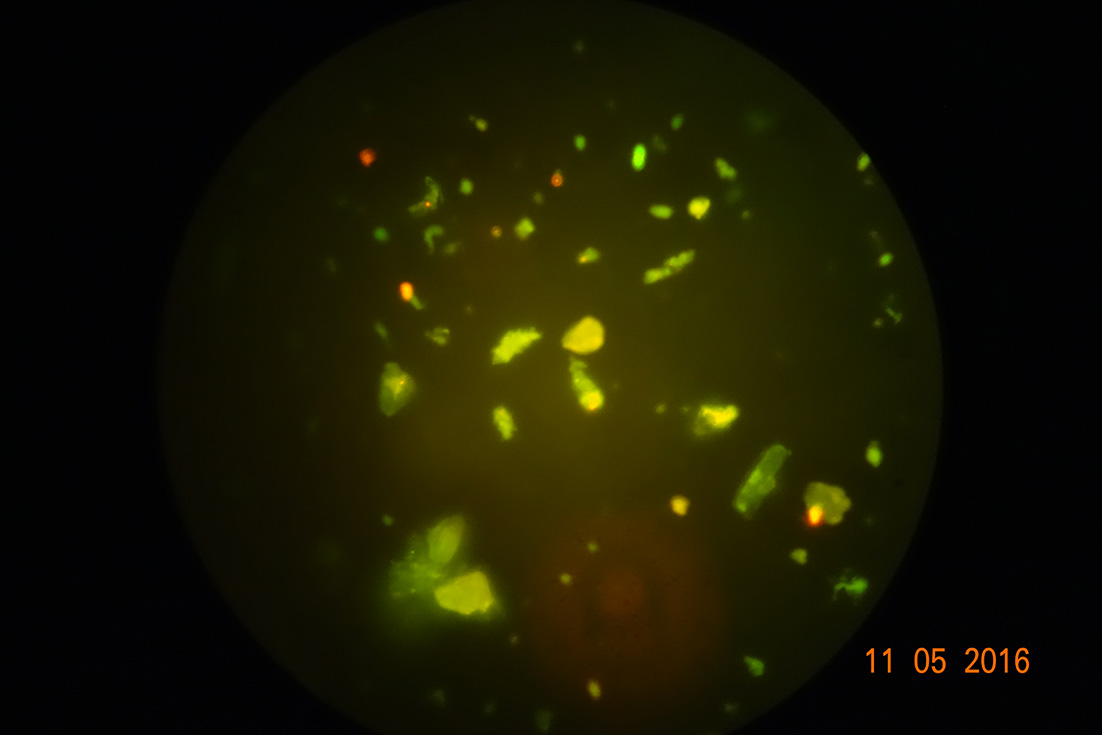

Supplement: S11 Supporting Information — (ZIP) [file pone.0218513.s015.zip › S11_Supporting Information.zip/cccp/DSC05034.JPG]

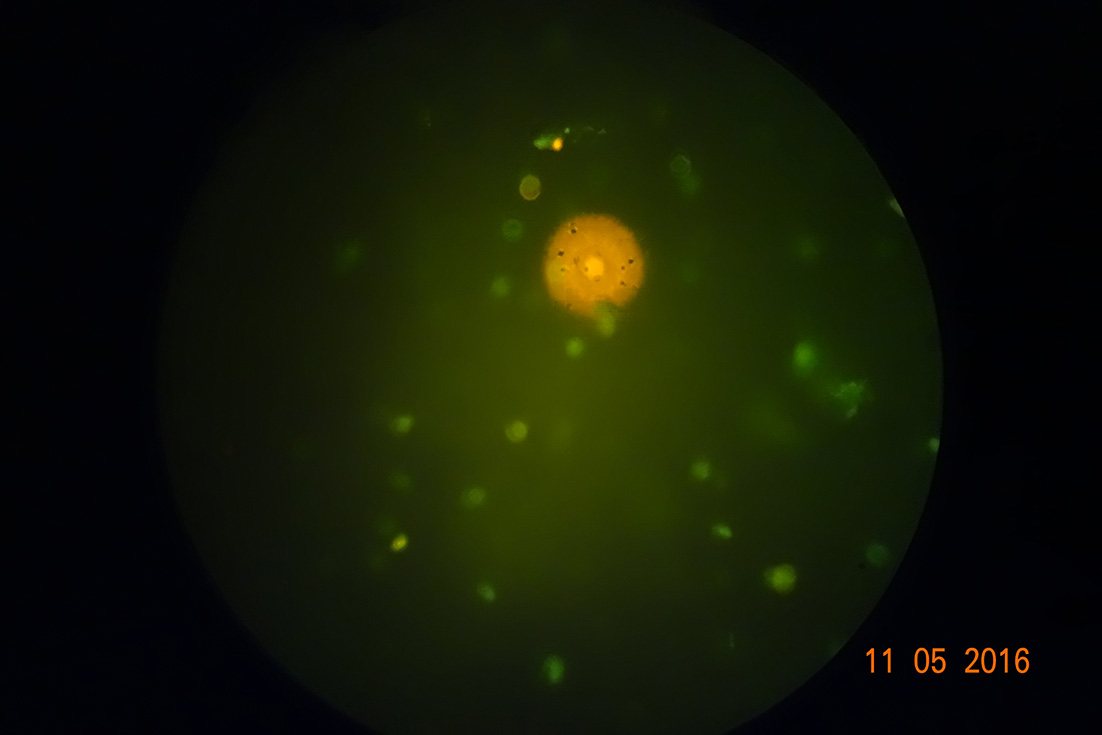

Supplement: S11 Supporting Information — (ZIP) [file pone.0218513.s015.zip › S11_Supporting Information.zip/cccp/DSC05035.JPG]

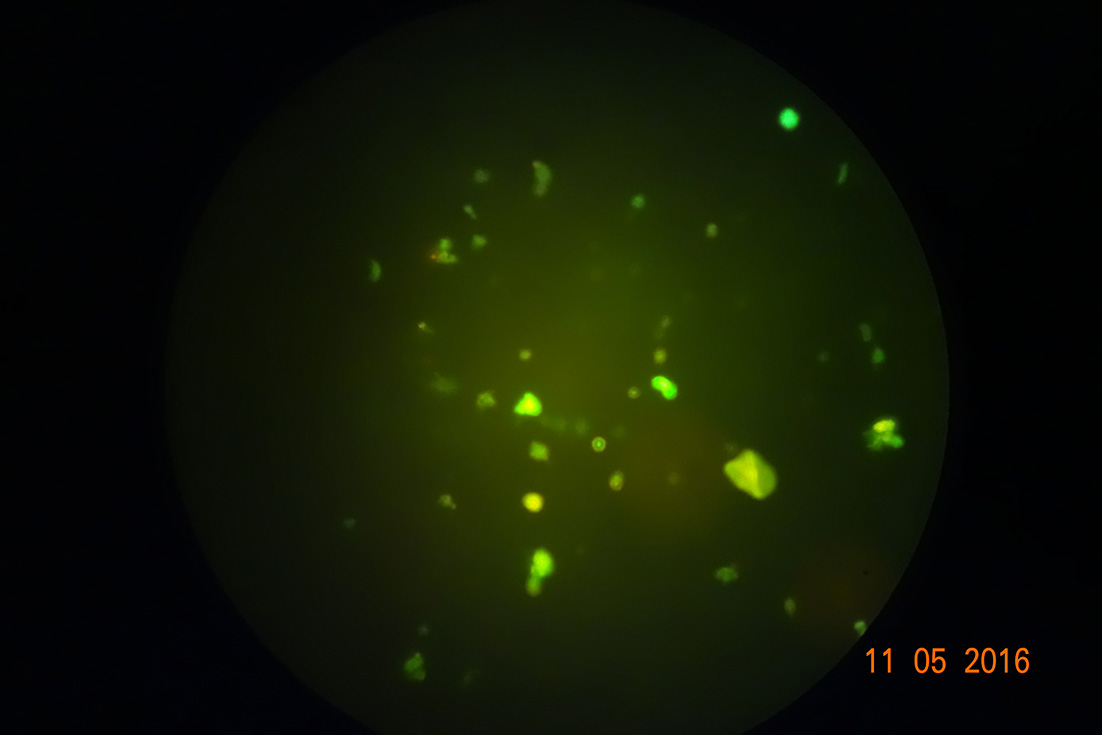

Supplement: S11 Supporting Information — (ZIP) [file pone.0218513.s015.zip › S11_Supporting Information.zip/cccp/DSC05036.JPG]

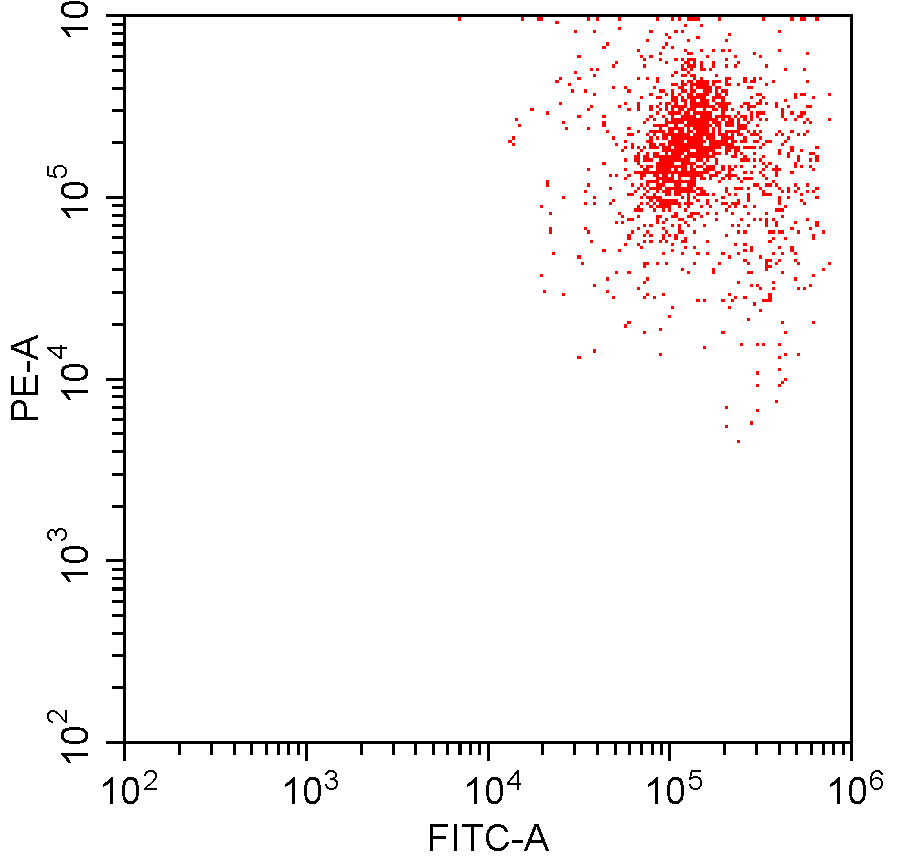

Supplement: S12 Supporting Information — (ZIP) [file pone.0218513.s016.zip › A.bmp]

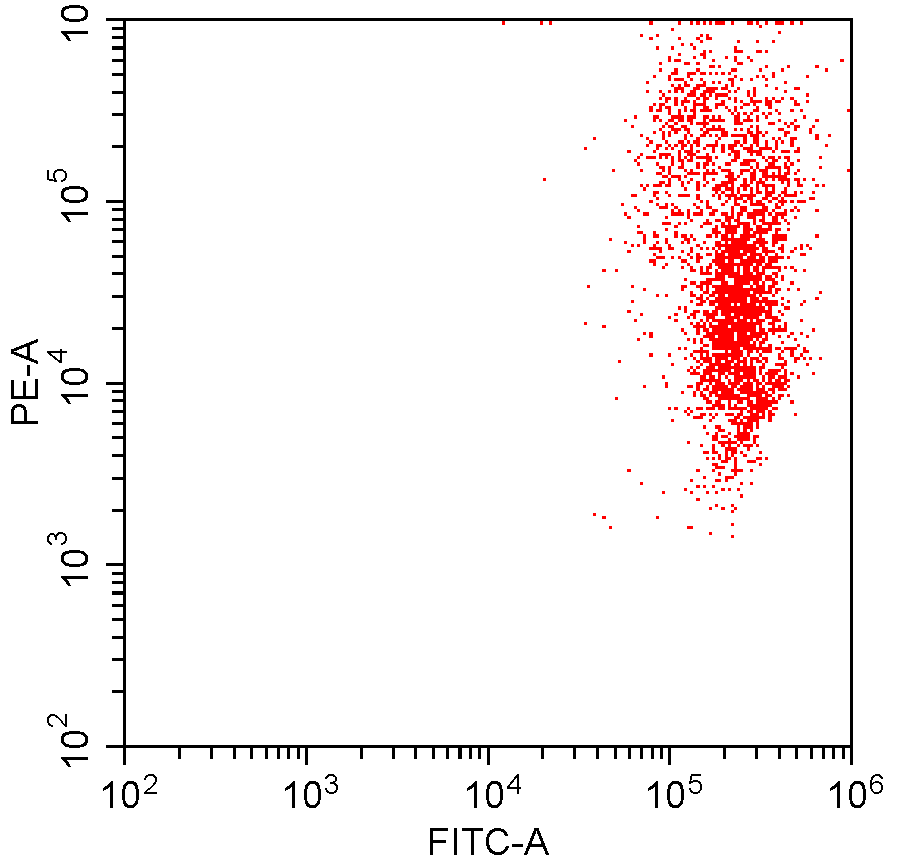

Supplement: S12 Supporting Information — (ZIP) [file pone.0218513.s016.zip › B.bmp]

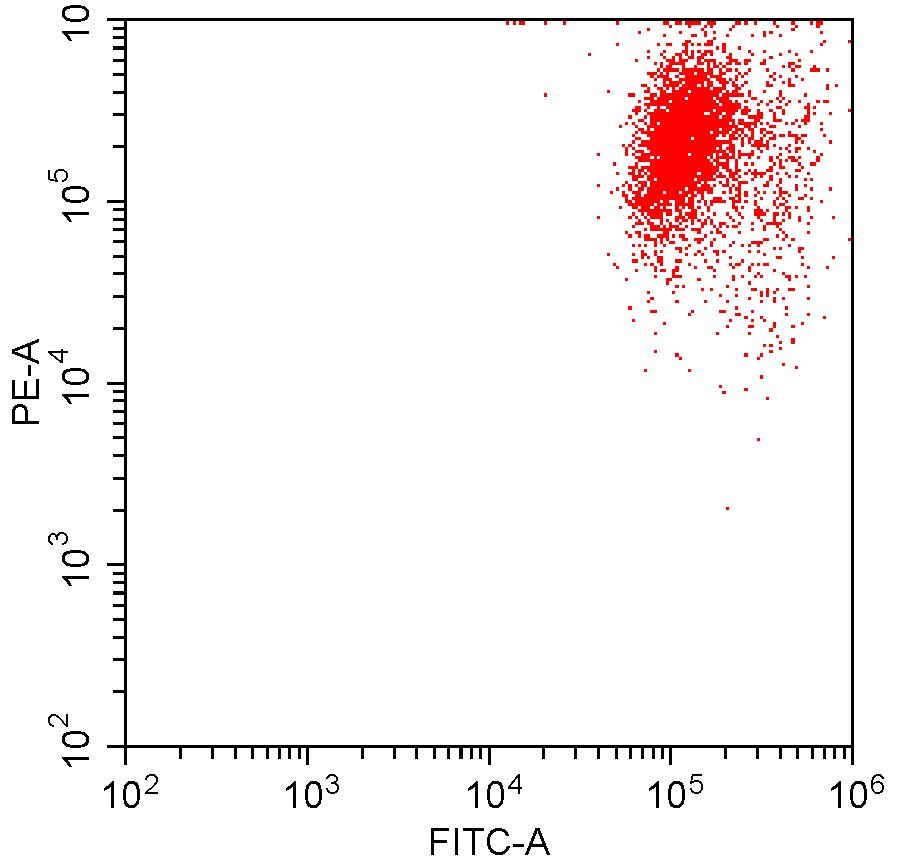

Supplement: S12 Supporting Information — (ZIP) [file pone.0218513.s016.zip › C.bmp]

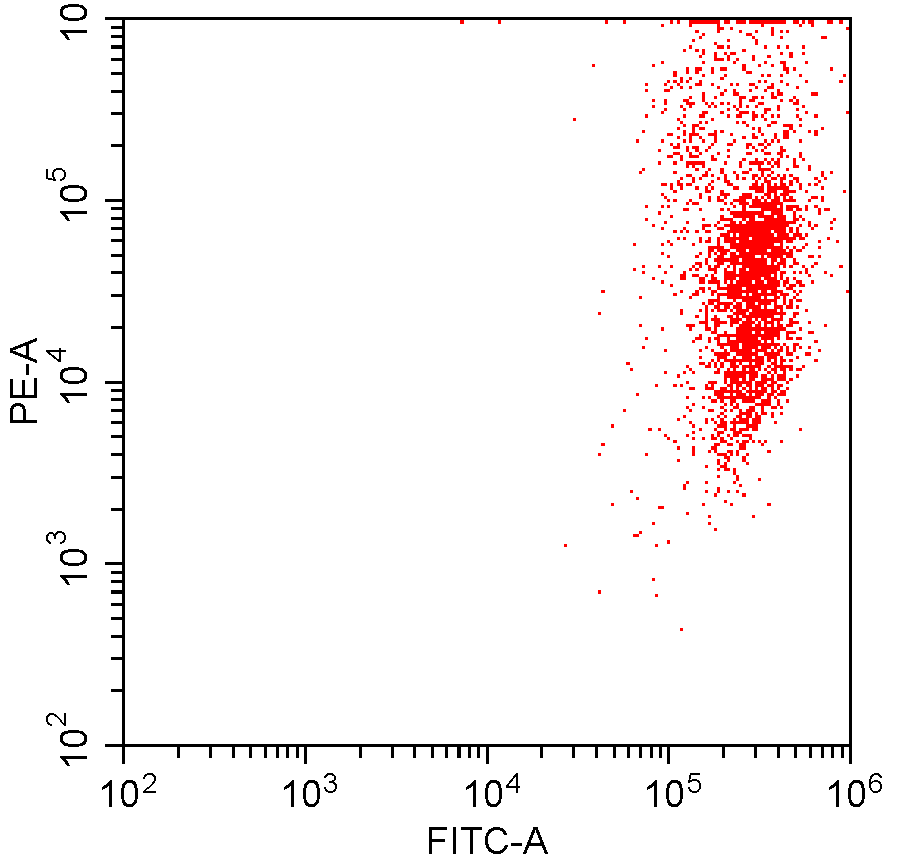

Supplement: S12 Supporting Information — (ZIP) [file pone.0218513.s016.zip › D.bmp]

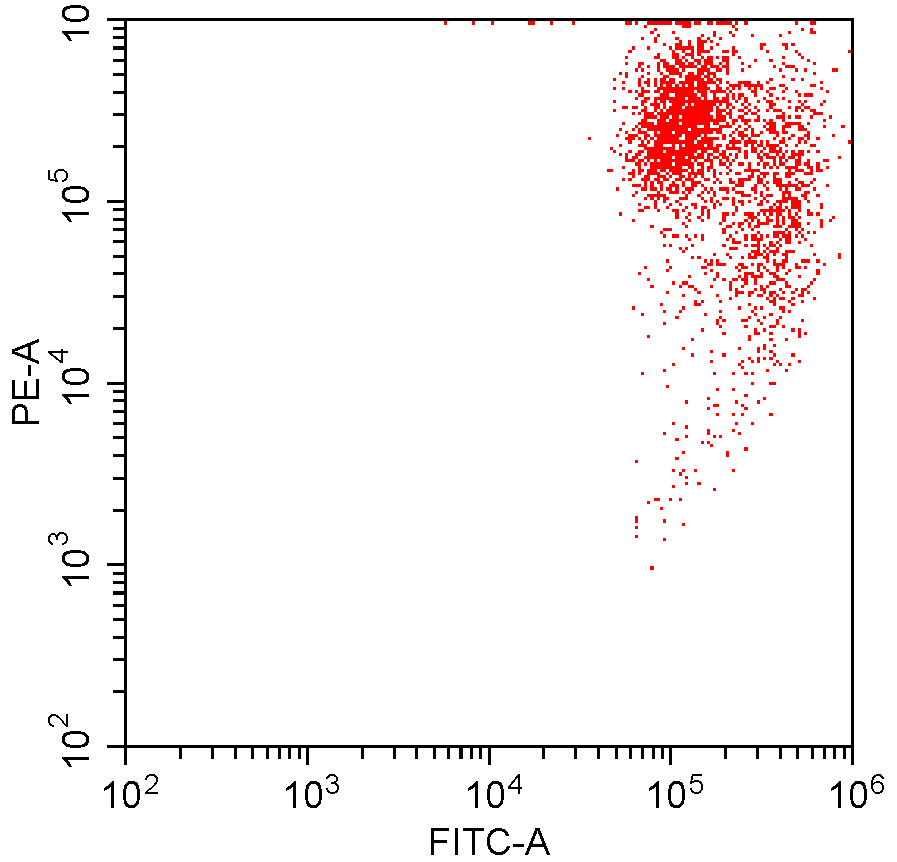

Supplement: S12 Supporting Information — (ZIP) [file pone.0218513.s016.zip › E.bmp]

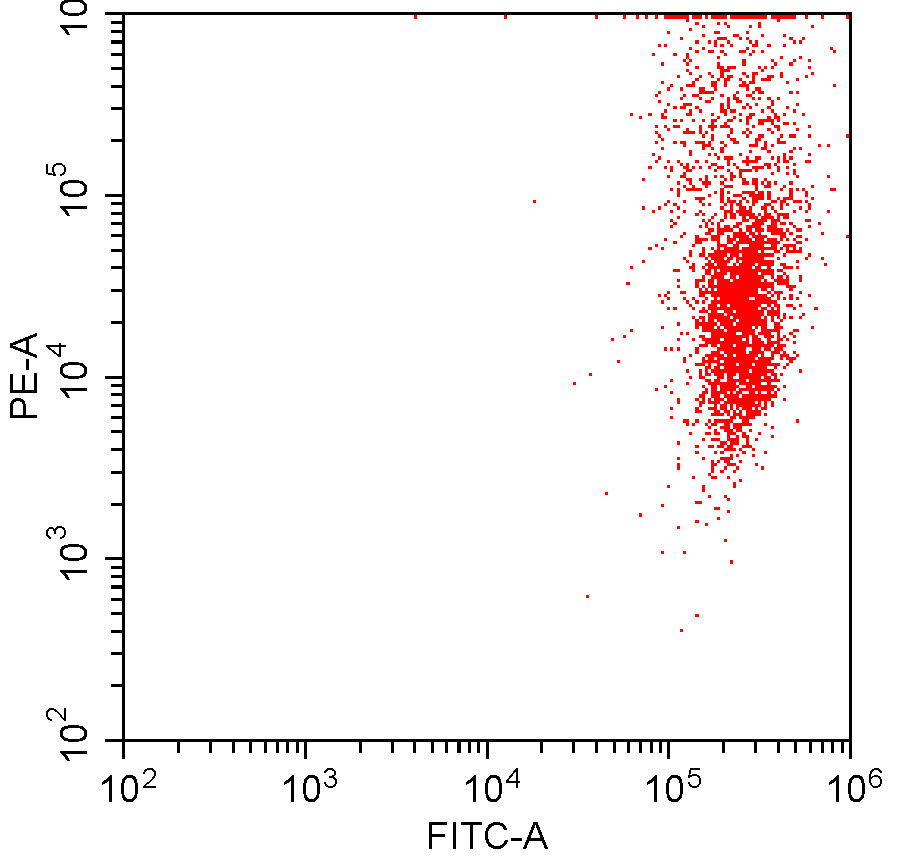

Supplement: S12 Supporting Information — (ZIP) [file pone.0218513.s016.zip › F.bmp]

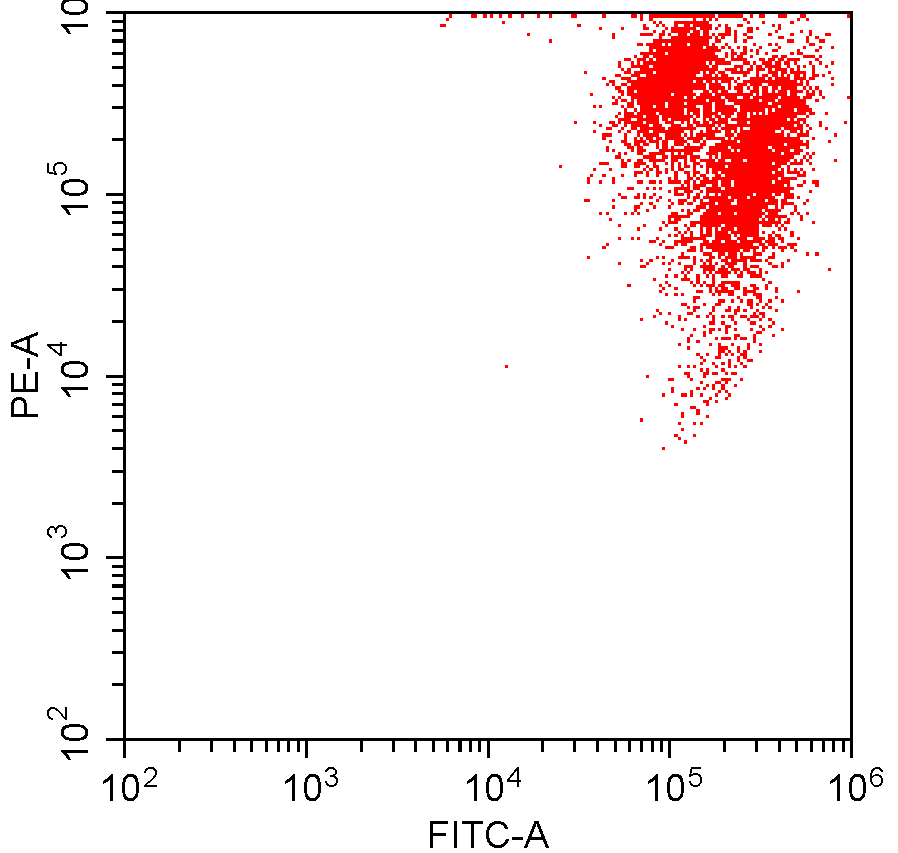

Supplement: S12 Supporting Information — (ZIP) [file pone.0218513.s016.zip › G.bmp]

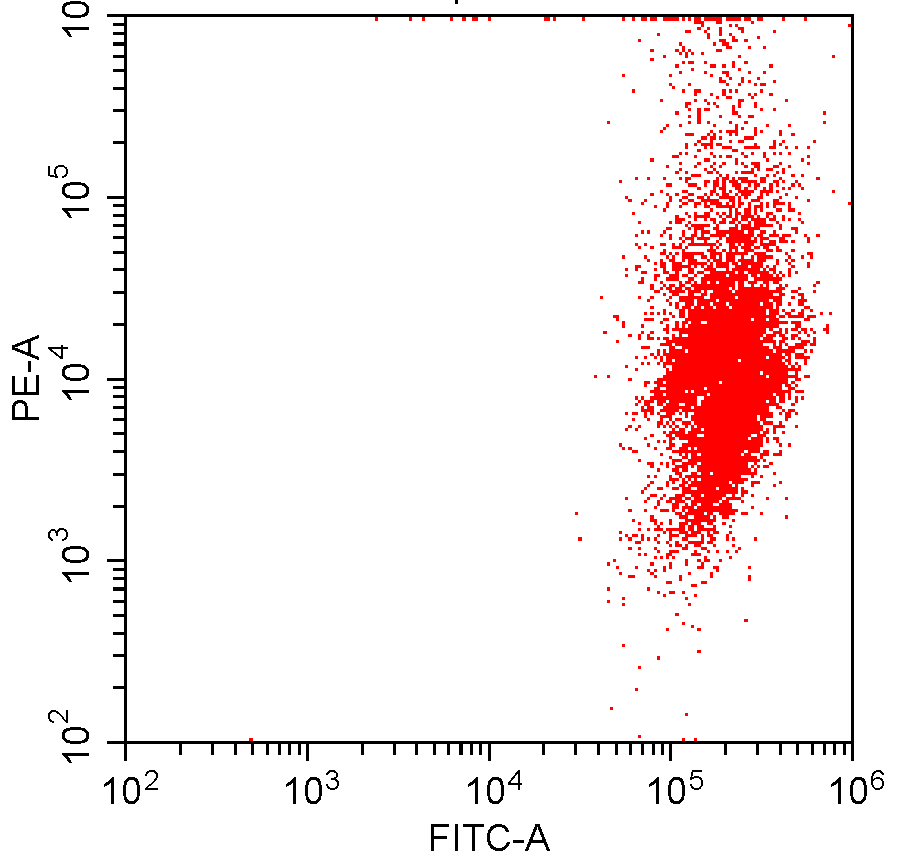

Supplement: S12 Supporting Information — (ZIP) [file pone.0218513.s016.zip › H.bmp]

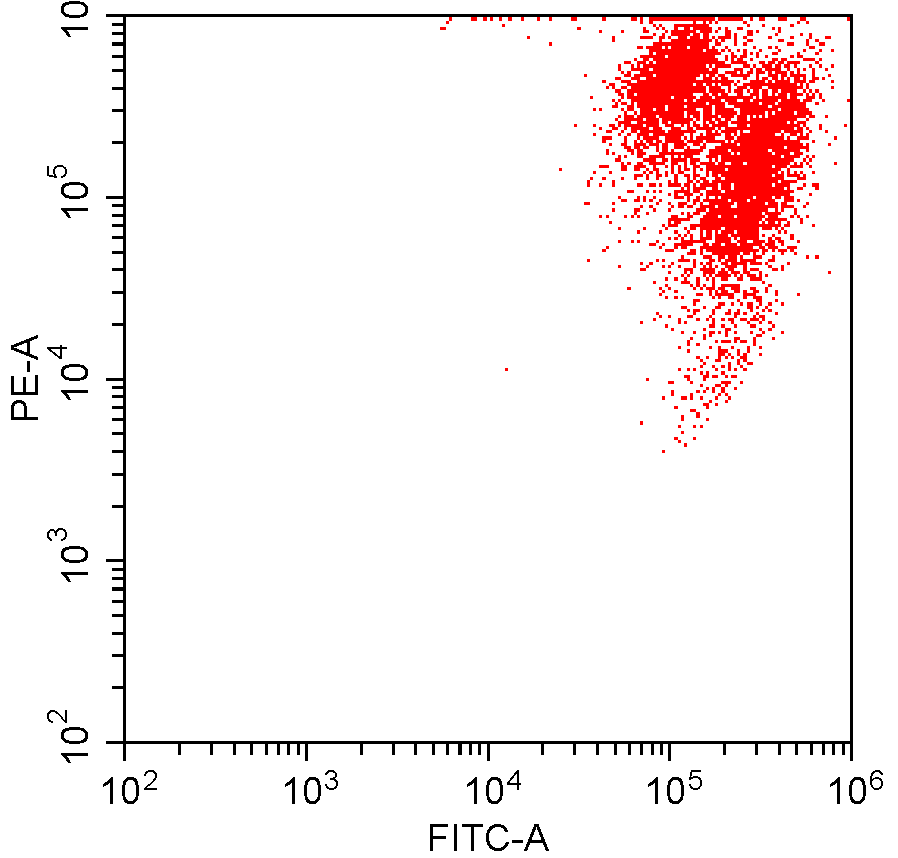

Supplement: S12 Supporting Information — (ZIP) [file pone.0218513.s016.zip › I.bmp]

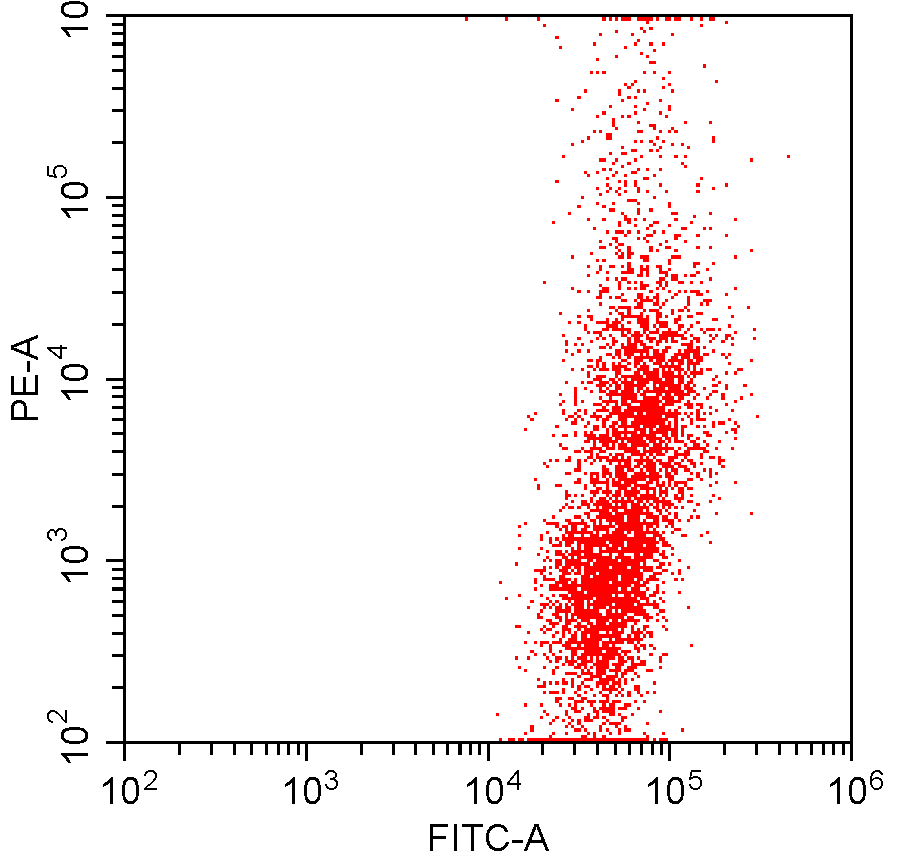

Supplement: S12 Supporting Information — (ZIP) [file pone.0218513.s016.zip › J.bmp]

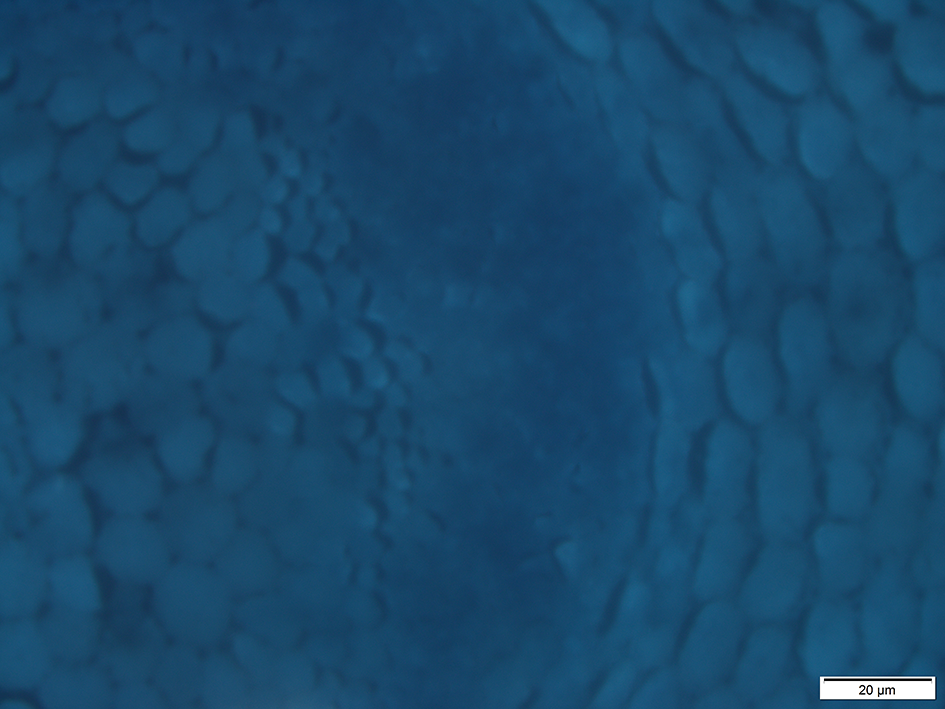

Supplement: S13 Supporting Information — (ZIP) [file pone.0218513.s017.zip › S13_Supporting Information.zip/A.tif]

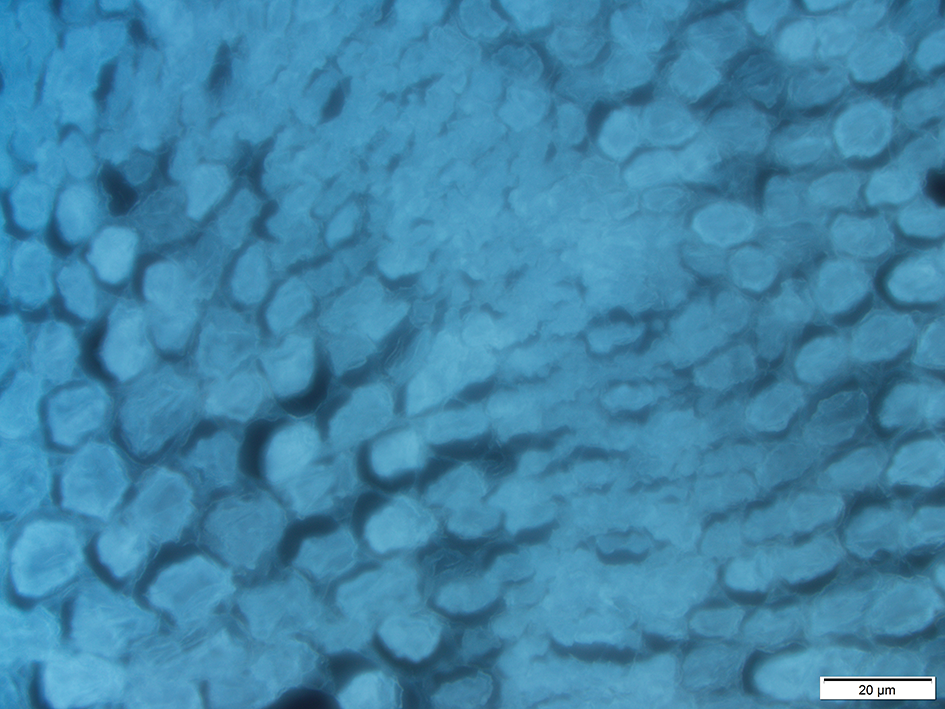

Supplement: S13 Supporting Information — (ZIP) [file pone.0218513.s017.zip › S13_Supporting Information.zip/B.tif]

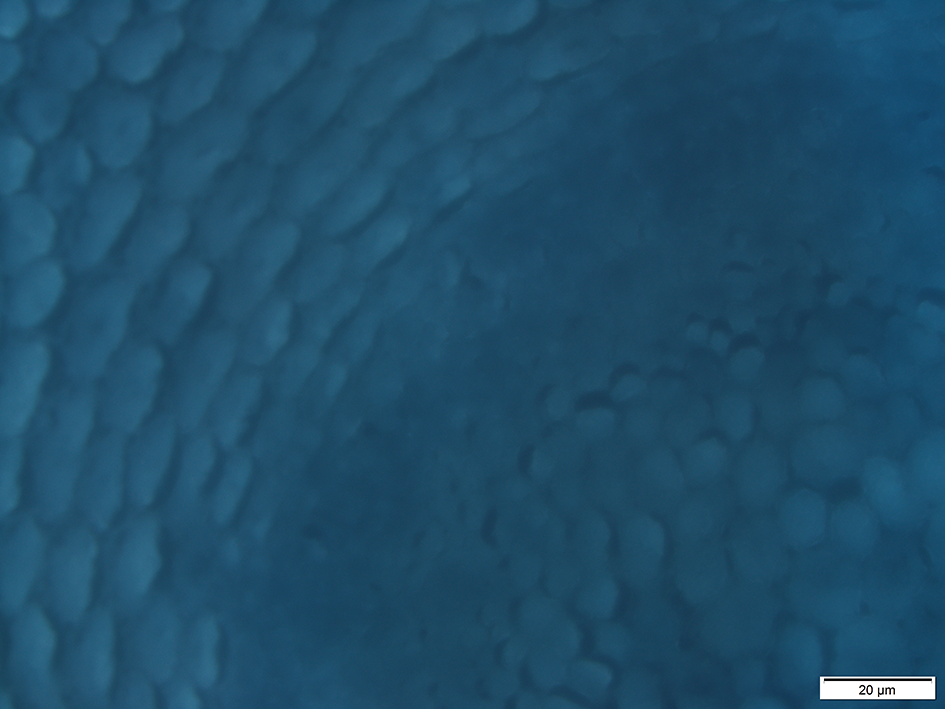

Supplement: S13 Supporting Information — (ZIP) [file pone.0218513.s017.zip › S13_Supporting Information.zip/C.tif]

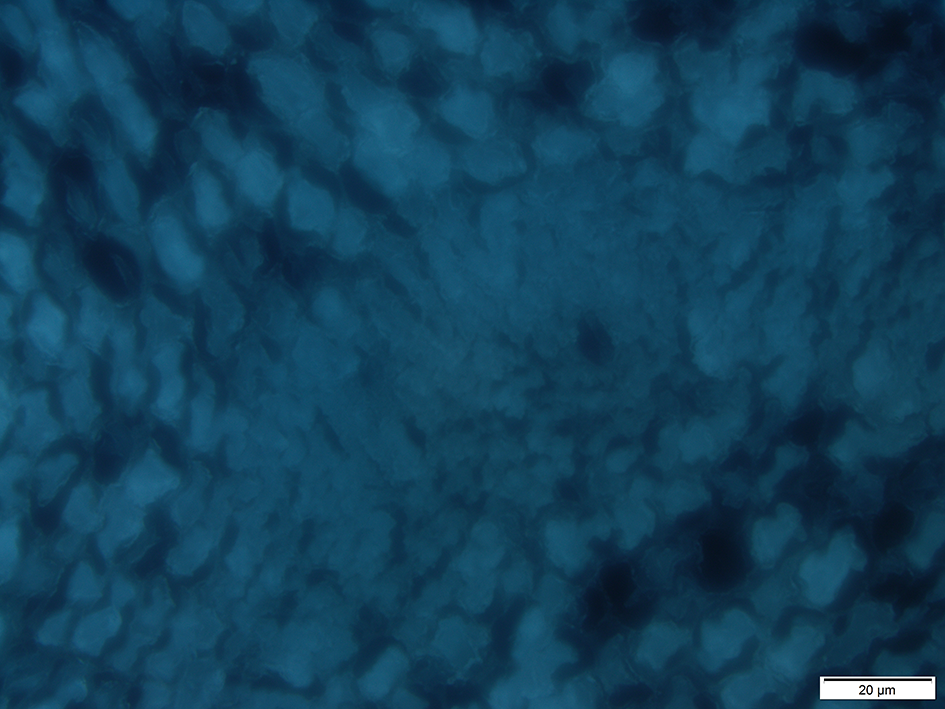

Supplement: S13 Supporting Information — (ZIP) [file pone.0218513.s017.zip › S13_Supporting Information.zip/D.tif]

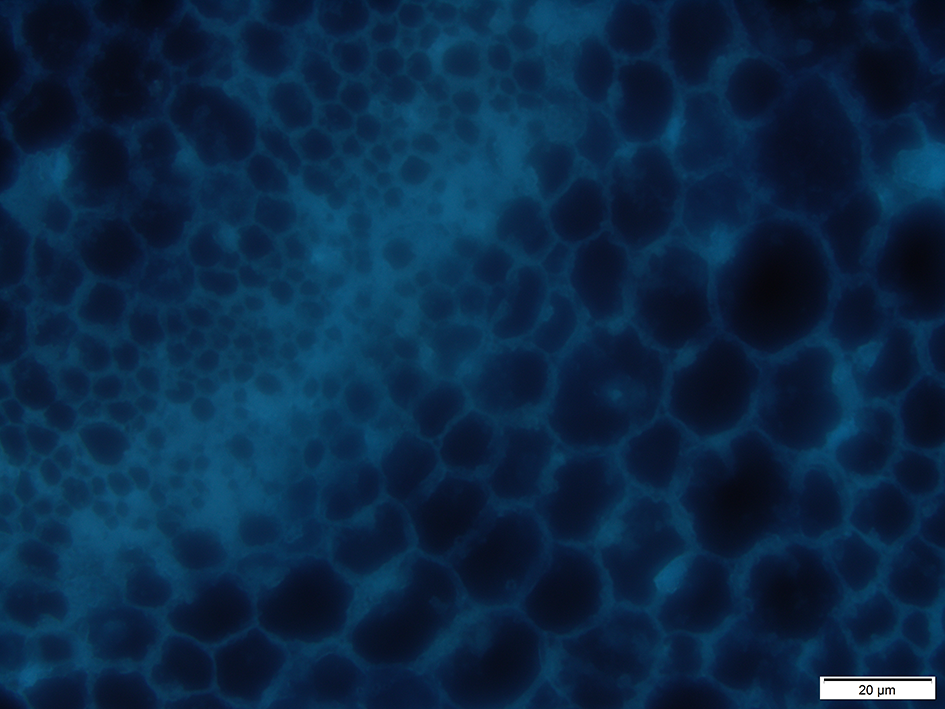

Supplement: S13 Supporting Information — (ZIP) [file pone.0218513.s017.zip › S13_Supporting Information.zip/E.tif]

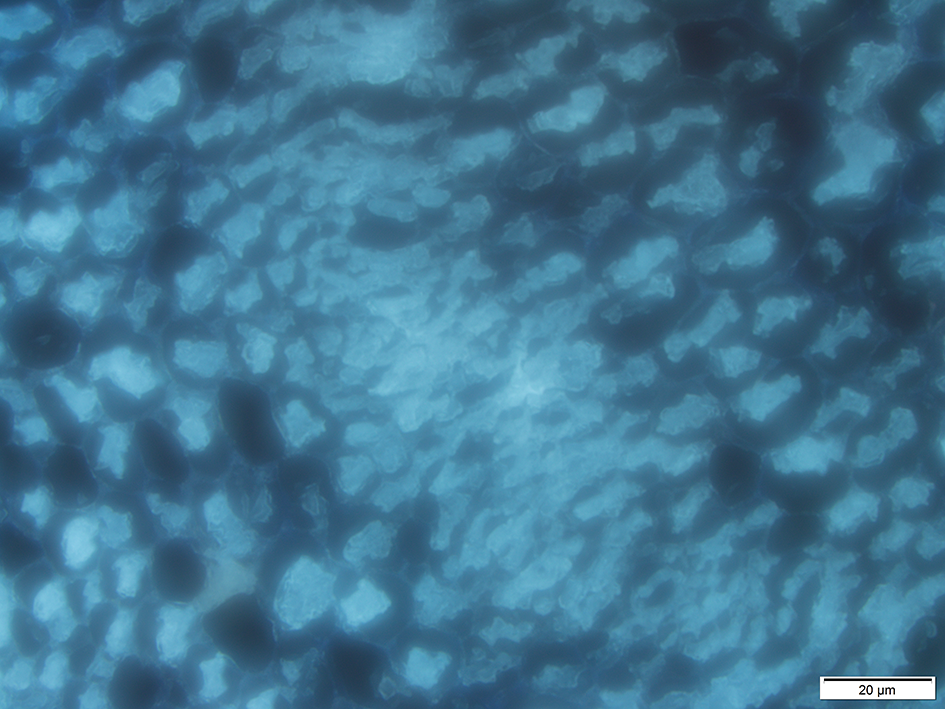

Supplement: S13 Supporting Information — (ZIP) [file pone.0218513.s017.zip › S13_Supporting Information.zip/F.tif]

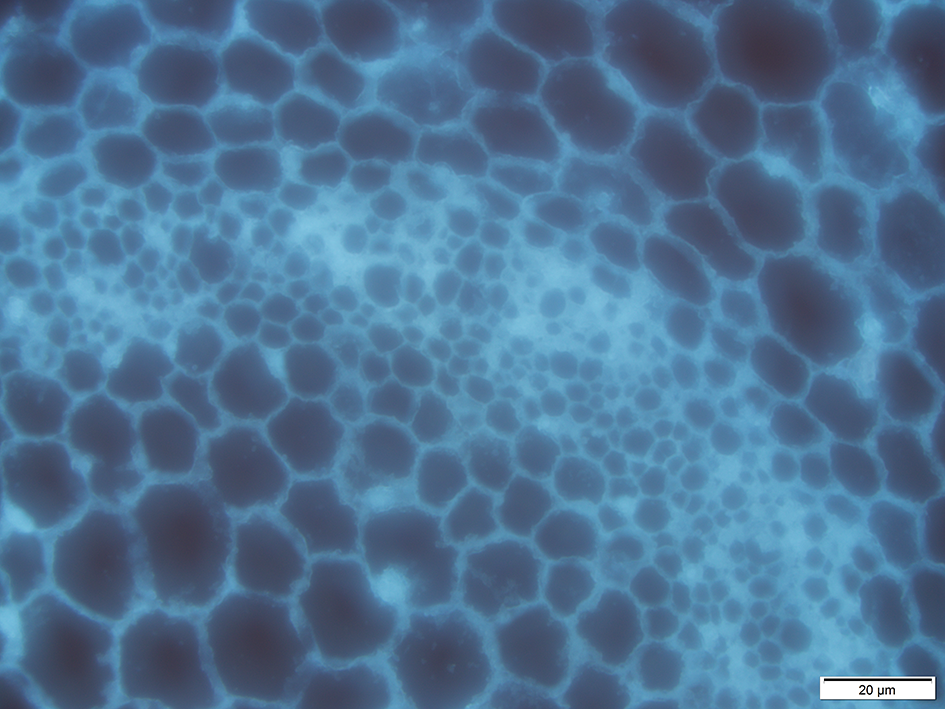

Supplement: S13 Supporting Information — (ZIP) [file pone.0218513.s017.zip › S13_Supporting Information.zip/G.tif]

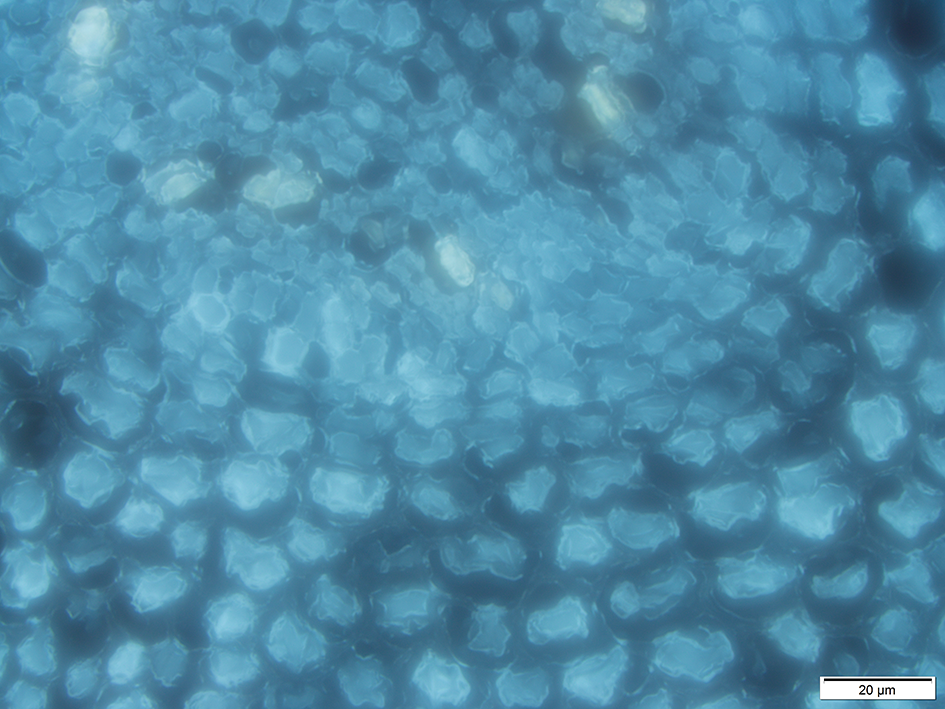

Supplement: S13 Supporting Information — (ZIP) [file pone.0218513.s017.zip › S13_Supporting Information.zip/H.tif]
